# What do we know about the effect of night-shift work on cardiovascular risk factors? An umbrella review

**DOI:** 10.3389/fpubh.2022.1034195

**Published:** 2022-11-23

**Authors:** Stephanie Boini, Eve Bourgkard, Jean Ferrières, Yolande Esquirol

**Affiliations:** ^1^Department of Occupational Epidemiology, French Research and Safety Institute for the Prevention of Occupational Accidents and Diseases (INRS), Vandœuvre-les-Nancy, France; ^2^UMR1295, Paul Sabatier III University – INSERM CERPOP: Centre for Epidemiology and Research in POPulation Health, Toulouse, France; ^3^Department of Cardiology, CHU Toulouse, Toulouse, France; ^4^Occupational Health Department, CHU Toulouse, Toulouse, France

**Keywords:** night-shift work, lipid disorders, overweight, smoking, sedentariness, psychosocial stressors at work, diabetes and hypertension, shift work

## Abstract

**Objective:**

To evaluate the existing evidence on the effect of night-shift work and its subtypes (permanent and rotating) on cardiovascular risk factors: diabetes, lipid disorders, being overweight, hypertension, smoking habits, sedentariness, and occupational psychosocial stressors.

**Method:**

A Web of Sciences and Cochrane review library search was conducted to identify systematic reviews with or without meta-analysis dealing with the quantification of the link between night-shift work and the studied cardiovascular risk factors in working populations. We used the AMSTAR 2 to evaluate the quality of each review. The main results of the included systematic reviews were compiled in a summary structured around the different cardiovascular risk factors.

**Results:**

After selection, 33 systematic reviews were included: nine for diabetes, four for lipid disorders, nine for being overweight, four for hypertension, two for smoking habits, three for occupational psychosocial stressors and two for sedentariness. The results confirmed an excess risk of diabetes of about 10% regardless of the type of night work. A stated excess risk of being overweight at around 25% was also highlighted for shift workers overall, which could reach 38% among night-shift workers. An increased risk of obesity, estimated at 5% for night-shift workers and at 18% for rotating shift workers, was observed. An excess risk of hypertension was estimated at around 30% when considering the broad definition of shift work and when night periods were included in rotating shifts. The literature provided inconsistent results for the link between lipid disorders and night-shift work. Shift workers appeared to be more likely to smoke. The link between shift work and occupational psychosocial stressors was scarcely explored in the available studies. Sedentariness was scarcely considered in systematic reviews, which prevents any firm conclusions.

**Conclusion:**

The consequences of night work in terms of diabetes, being overweight/obesity and hypertension are established. Monitoring of these cardiovascular risk factors for these night-shift workers could be implemented by practitioners. In contrast, the links with lipid disorders, sedentariness, smoking habits, and occupational psychosocial stressors warrant further investigation.

**Systematic review registration:**

https://www.crd.york.ac.uk/prospero/display_record.php?ID=CRD42021275212, PROSPERO (ID CRD42021275212)

## Graphical Abstract

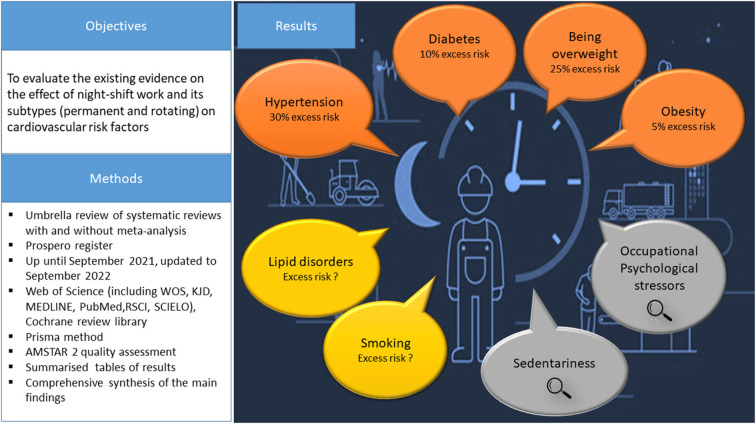

## Context

Despite advances in diagnosis and in treatment, cardiovascular disease has remained a major concern over the last few decades, having almost doubled worldwide between 1990 and 2019 (1). Although a significant decline in age-standardized mortality rates and, to a lesser extent, age-standardized prevalence rates over the past 20 years, cardiovascular disease has remained among the leading cause of mortality and morbidity worldwide (1). Diabetes, hypertension, being overweight, sedentariness, lipid disorders and tobacco consumption remained the main modifiable risk factors contributing to the global burden of cardiovascular diseases in 2019 (1). Early improvement of these modifiable well-known cardiovascular risk factors remains the challenge fixed by all the experts in acute guidelines (2).

However, the literature provides more and more articles on the potential effect of working conditions on these cardiovascular risk factors (3, 4). Some of these working conditions are modifiable or at least adaptable. Among them, night-shift work is closely scrutinized. Although regulated, the use of such working-time patterns is not marginal. Approximately 19% (24% men; 14% women) of workers in the EU carried out night work in 2016 (5). An increased risk of any cardiovascular disease has been observed in night-shift workers, up to 40% (6–8). The pathophysiological mechanisms explaining the associations between shift work and cardiovascular disease rely on several complex and interrelated pathways, including cardiovascular risk factors as mediators (9). Circadian stress due to night-shift work can indeed induce physiological, behavioral, and psychosocial stress that leads to health conditions predictive of cardiovascular diseases. Therefore, a substantial literature on the effects of shift work on cardiovascular risk factors has been provided over the last few decades, leading to the publication of numerous systematic reviews on this topic. The time has now come to say what we know and what we intend to do about it over the next few years.

This umbrella review aimed to evaluate the existing evidence on the effect of night-shift work and its subtypes (permanent and rotating) on cardiovascular risk factors—these being diabetes, lipid disorders, being overweight, hypertension, smoking habits, sedentariness, and occupational psychosocial stressors.

## Methods

### Search strategy

The search strategy was determined *a priori*, and the protocol was registered to PROSPERO (ID CRD42021275212). No deviation from the PROSPERO protocol was made.

A literature search was conducted to retrieve eligible systematic reviews with or without meta-analysis that addressed the above research objective. Flowcharts, using the PRISMA method (9, 10), summarized the different steps of the article selection for each investigated cardiovascular risk factor. The following databases were searched for eligible reviews from inception to September 2021, updated to September 2022: Web of Science (including WOS, KJD, MEDLINE, PubMed, RSCI, SCIELO) and Cochrane review library. Moreover, screening the reference lists of the selected papers completed the searches. Relevant reviews listed and not found by the main step of research strategy were integrated, as it needed.

For working hour schedules, the following MeSH terms and key words included in “title” or in “abstract” were used: shift work, shift workers, night workers, night work, night shift work, night shift workers, night-shift work, night-shift workers, shift working, rotating shift, rotating shift workers and irregular working hours.

These terms were successively combined with several term groups including the following MeSH terms or keywords in “title” or in “abstract”:

1) **diabetes:** diabetes, glycemia, glucose, diabetes mellitus, NIDDM, non-insulin-dependent, type 2 diabetes and truncated terms: diab^*^.
2) **lipid disorders:** lipids, cholesterol, triglycerides, apolipoprotein, chylomicron, very low-density lipoprotein, low-density lipoprotein, high-density lipoprotein (HDL) and truncated term: lip^*^.
3) **being overweight:** body mass index, weight, obesity, obese, waist circumference.
4) **hypertension:** blood pressure, hypertension, high blood pressure, systolic pressure and diastolic pressure.
5) **smoking habits:** tobacco, smoking.
6) **sedentariness:** sedentary, sedentariness, sedentarity, physical activity.
7) **occupational psychosocial stressors:** psychosocial risk, psychosocial factors, stress.

The detailed search strategy is provided in Supplementary Table A.

### Inclusion and exclusion criteria and articles selection

Any systematic review published in English or French, dealing with quantification of the relationship between night-shift work and studied cardiovascular risk factors in the working population was included. Narrative, comprehensive and mechanistic reviews were excluded, as well as research protocol without any result. Reviews focusing on long working hours, or atypical or irregular working hours were not considered here. The list of excluded articles with the reason of exclusion is available in Supplementary Table B.

For each investigated topic, two out of three independent experts in occupational health and cardiovascular epidemiology areas (YE, SB, EB) independently conducted the articles selection by using defined search terms in databases. The results obtained from the search were exported to EndNote X8TM (Clarivate Analytics, Philadelphia, USA), which enabled to identify the duplicates and to remove them.

The next step involved selecting articles based on the screening of titles and abstracts independently conducted by two out of three experts (SB, EB, YE), who also conducted a further selection after reading the full articles. If there was a disagreement, a discussion with a fourth expert (JF) resolved it. All steps were clearly reported.

### Data extraction

Two out of three experts (YE, SB, EB) independently extracted the following information from each included systematic review:

- citation details
- objective of the systematic review or meta-analysis
- type of databases sourced and searched and date range of database searching publication
- information about the studies included in the review: publication date range, number and type of studies, country of origin, rating of the quality of the studies, details of participants and setting/context
- types of exposure to working hour schedules and their duration
- definition of the examined outcome and its assessment
- adjustment factors
- main results including the summary of the effect size estimate [risk ratio (RR), odds ratio (OR), hazard ratio (HR), or incident risk ratio] with the 95% confidence intervals (CIs)

If there was a disagreement, a discussion with the third expert resolved it.

### Quality assessment of the selected articles—Risk of bias

To evaluate the quality of the reviews, we used AMSTAR 2 (a measurement tool for assessing systematic reviews, version 2) (11). The sixteen items assessed were: 1: complete research question and criteria (PICO); 2: registered protocol; 3: justification of study design; 4: comprehensive literature search; 5: study selection in duplicate; 6: data extraction in duplicate; 7: justification of excluded studies; 8: description of included studies; 9: assessing the risk of bias (RoB); 10: reporting on the sources of funding for the studies included; 11: meta-analysis using appropriate statistical methods combining results; 12: meta-analysis assessing the impact of RoB; 13: interpretation/discussion of results must include risk of bias of studies; 14: discussion of heterogeneity; 15: investigation of publication bias in meta-analysis; 16: reporting conflict of interest. All included systematic reviews were rated as “yes”, “partial yes” or “no” according to the AMSTAR-2 checklist.

### Strategy for data synthesis

A narrative synthesis of the main findings from the included studies described in data extraction tables was conducted and structured around the type of cardiovascular risk factors.

## Results

**Figure 1:**
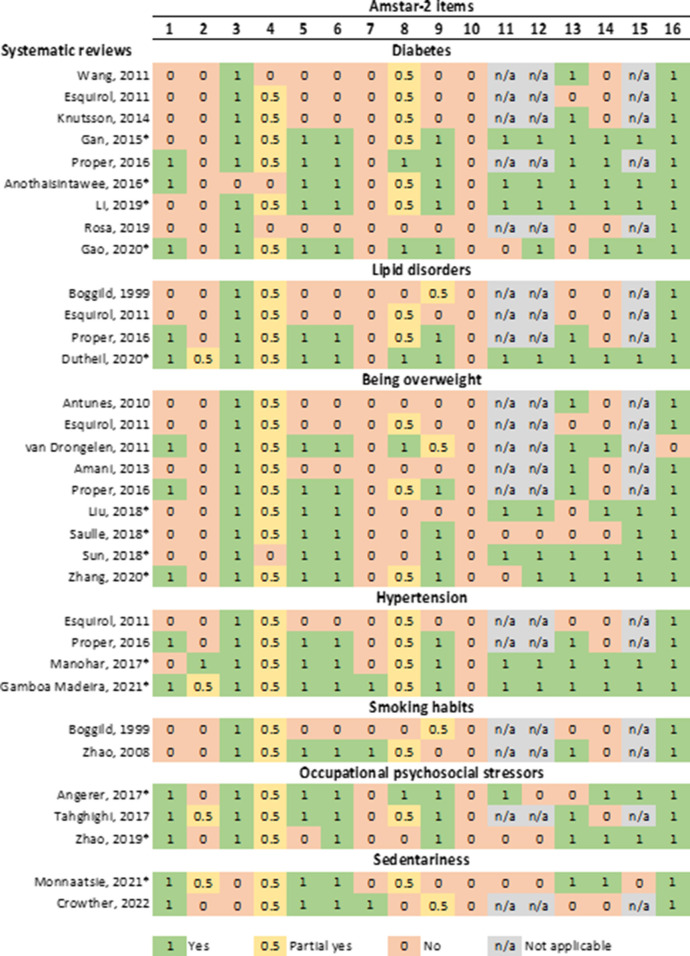
Quality assessment of included systematic reviews on the link between night-shift work and cardiovascular risk factors using AMSTAR-2 (*systematic review with meta-analysis). Footnotes AMSTAR-2 items-1, complete research question and criteria (PICO); 2, registered protocol; 3, justification of study design; 4, comprehensive literature search; 5, study selection in duplicate; 6, data extraction in duplicate; 7, justification of excluded studies; 8, description of included studies; 9, assessing the risk of bias (RoB); 10, reporting on the sources of funding for the studies included; 11, meta-analysis using appropriate statistical methods combining results; 12, meta-analysis assessing the impact of RoB; 13, interpretation/discussion of results must include risk of bias of studies; 14, discussion of heterogeneity; 15, investigation of publication bias in meta-analysis; 16, reporting conflict of interest.

One thousand and forty-five (1,045) reviews were identified according to the eligibility criteria for all studied cardiovascular risk factors, and the results were as follows: 205 for diabetes, 41 for lipid disorders, 234 for being overweight, 121 for hypertension, 60 for smoking habits, 122 for sedentariness, and 262 for occupational psychosocial stressors. After screening, 31 full texts of systematic reviews were retained. Two additional systematic reviews on sedentariness were identified during the update period. Seven separate flowcharts summarized the selection process using the PRISMA guidelines and the reasons of some full text exclusion (Supplementary Figure A). Main findings were described by cardiovascular risk factors, including the quality assessment of each included systematic review according to the AMSTAR-2 checklist (Figure 1).

### Diabetes

The Supplementary Figure A displays the different steps of the reviews selection related to the relationship between night-shift work and diabetes. After reading the full text of 26 reviews, 17 were excluded mainly because they were narrative (53%) or did not evaluate the association of interest (35%). Nine systematic reviews with or without meta-analyses were finally considered for this purpose: five systematic reviews (12–16) and four meta-analyses (17–20) (Supplementary Table C).

According to the AMSTAR-2 checklist (Figure 1), the four meta-analyses and only 1 review (14) met most of the quality criteria (68–77% of criteria completely or partially met). Item no. 4 (comprehensive literature search) and item no. 8 (description of included studies) were often partially met. The criteria related to an *a priori* protocol registration (item no. 2), to the justification of excluded studies (item no. 7) and to the reporting on the sources of funding for the studies included (item no. 10) were never provided. The oldest systematic reviews (12, 13, 16) did not report in detail the method used to perform the systematic review (AMSTAR-2 items no. 3, 4, 5, 6).

In total, fifty-one primary studies were analyzed in the different systematic reviews with and without meta-analyses, covering a period of 20 years (from 1999 to 2018, only one study was published in 1983). Among the 5 systematic reviews without meta-analyses, three systematic reviews were based on longitudinal studies (13–15). Esquirol et al. included 13 longitudinal and 11 cross-sectional studies published between 2001 and 2010 (12). Later, Proper et al. considered 9 longitudinal studies published from 2005 to 2015 (14). Rosa et al. conducted a systematic review on the global health of shift work in nurses and retained only one study on diabetes (15). The four systematic reviews with meta-analyses did not consider all studies referenced in two systematic reviews previously performed (12, 14). Two systematic reviews with meta-analyses only considered the results of longitudinal studies (17, 20). The four systematic reviews with meta-analyses were published between 2015 and 2020 (17–20). Li et al. (20) performed an updated meta-analysis undertaken by Gan et al. (18) and only included cohort studies. Anothaisintawee et al. focused on sleep disturbance and conducted a meta-analysis on a subgroup of 10 studies undertaken specifically on shift work (17).

In each systematic review with meta-analyses, the number of studies varied from 11 to 21 (prospective studies from 6 to 12), with the number of participants ranging from 226,652 (18) to 639,880 (19), mixing men and women from different occupations (industry or healthcare or heterogeneous). The quality of the included studies was systematically evaluated by specific quality tools (e.g., NOS, AHRQ checklist), with a level of satisfactory quality retained by the authors. The diabetes diagnosis was based on fasting plasma glucose (FPG), glycated hemoglobin (HbA1c), oral glucose tolerance test (OGTT), a diabetes death certificate, a self-report or a medical report.

An excess risk estimated at around 10% of developing diabetes among shift workers compared to day workers was consistently found in the meta-analyses. The statistically significant overall pooled adjusted ORs ranged from 1.08 to 1.15 for shift work, from 1.08 to 1.42 for rotating shift work and from 1.09 to 1.19 for night-shift work. This increased risk seems to be higher in men (18, 20). A dose-response effect was highlighted in female shift workers only, with an increased risk of 5-7% every 5 years (19, 20) (Table 1).

**Table 1:** Main results of systematic reviews focused on the link between shift work and diabetes.

**References, Country**	**Type-Shift work (*n*)**	**Assessment of** **outcomes (*n*)**	**Confounding factors (*n*)**	**Main results shift work vs. day work (*n*)**
Wang et al. (16), UK	Rotating (4) Unspecified (2)	Type 2 diabetes (1) Diabetes mellitus (5) Diabetes death certificate (1) FPG or patient under treatment (1) MD (4)	NA (1) Adjustments (5)	Rotating: HR = 1.67 (0.57–4.90) (♂ 1); Rotating 2-shift RR = 1.73 (0.85–3.52)/Rotating 3-shift RR = 1.33 (0.74–2.36) (♂ 1) Shift work: Age groups (♂ 1); (30–39 yo) OR = 6.75 (1.31–56.1); (40–49 yo) OR = 1.22 (0.68–2.10); (50–59 yo) OR = 0.93 (0.53–1.55) Shift work: diabetes mortality: per year β × 10^−5^ = 4.14 (2.46–5.81) (♂ 1) Exposure duration: Rotating night ≥10 years adjusted for age: RR = 1.64 (1.11–2.37); multiple adjustment including BMI: RR = 0.98 (0.66–1.45) (♀ 1)
Esquirol et al. (12), France	Permanent Night (3) Rotating (21) Unspecified (2) Evening (1)	Type 2 Diabetes (1) Diabetes mellitus (23) Glycaemia (11) HbA1c (8) FPG (2) OGTT (2) RPG (1) Self-reported (1) Unknown (2)	NA(11) Adjustments (13)	Longitudinal studies: significant effect (7): Shift work: OR = 1.56 (1.18–2.05) (1); OR = 1.35 (1.05–1.75) (1) Shift work duration ♂: age > 50 yo and 19–32 years exposure: 5% increase of risk (1) Shift work: Glycaemia>110 mg/dL = 5.1% vs. 3.8% for DW (1) Cross-Sectional studies: significant effect (4): Night shift: OR = 1.7 (0.8–3.6) (1); 2-shift: OR = 2.5 (1.1–4.3) (1) Longitudinal studies non-significant effect (13)
Knutsson and Kempe (13), Sweden	Rotating (5)	Type 2 diabetes (5) Type 1 diabetes (1) OGTT (1) Death certificate (1) Self-reported (1) HbA1c (2)	NA(1) Adjustments (4)	Rotating ♂: HR = 1.67 (0.59–4.90) (1); SRR = 1.24 (0.91–1.70)/SRR = 2.29 (0.97–5.40) (1) OR = 1.35 (1.05–1.75) (1) 2-shift ♂: RR = 1.73 (0.85–3.52) (1); 3-shift ♂: RR = 1.33 (0.74–2.36) (1) Rotating duration ♂: 10–19/20–29/≥30 years: SRR = 1.41 (0.18–11.30)/1.92 (0.5– 7.32)/2.85 (1.15–7.08) (1) Rotating duration ♀: 1–2/3–9/10–19/≥ 20 years: HR = 1.03 (0.98–1.08)/1.06 (1.01–1.11)/1.10 (1.02–1.18)/1.24 (1.13–1.37) (1) Rotating ♀: HR per 5 years = 1.05 (1.04–1.06) (1)
Gan et al. (18), China Meta-analysis	Night shift (3) Rotating (3 × 8) (4) Mixed rotating (2 × 8/3 × 8) (2) Unspecified (2)	Type 2 diabetes (4) Diabetes mellitus (7) Diabetes death certificate (1) Self-reported or medical/register report (10) FPG (2) HbA1c (2)	NA (1) Adjustments (11)	Shift work: RR = 1.08 (1.05–1.12), *I*^2^ 40.9% (corrected for publication bias) (11) Night shift: OR = 1.09 (1.04–1.14), *I*^2^ 37.6% (3) Rotating shift: OR = 1.42 (1.19–1.69), *I*^2^ 13.4% (4) Subgroup analyses Shift work: cohort studies OR = 1.12 (1.06–1.19), *I*^2^ 52.9%/cross-sectional studies OR = 1.06 (1.03–1.09), *I*^2^ 10.9% Shift work: OR = 1.09 (1.04–1.14) for ♀, *I*^2^ 54.3%/OR = 1.37 (1.20–1.56) for ♂, *I*^2^ 0.0%
Proper et al. (14), Netherlands	Permanent Night (1) Rotating (8)	Type 2 diabetes (2) Diabetes mellitus (7) Glycaemia (4) OGTT (1) RPG (1) FPG (1) HbA1c (4)	NA (2) Adjutments (7)	Shift work: High FPG: age (25–29 yo) 32.9 vs. 23.1%; NS for other age classes (1) HbA1c OR = 1.35 (1.05–1.75) (1) Shift work: ♂ Positive relation with HbA1c; ♀ NS (1) Shift work: no association with FPG (2) Rotating: OR = 1.56 (1.18–2.05) (1) Rotating 3-shift: OR = 2.62 (2.17–3.17); Rotating 2-shift: OR = 1.78 (1.49–2.14) (1) Rotating 3-shift: RR = 1.33 (0.74–2.36); Rotating 2-shift: RR = 1.73 (0.85–3.52) (1) Shift duration: OR per 10 years = 1.05 (1.01–1.09) (1)
Anothaisintawee et al. (17), Thailand Meta-analysis	Rotating (5) Unspecified (6)	Type 2 Diabetes (4) Diabetes mellitus (7) FPG (4), HbA1c (3) OGTT (2) Self-reported (4) Medical diagnosis or treatment (6)	Adjustments (8) Unspecified (2)	Shift work: RR = 1.40 (1.18–1.66), *I*^2^ 95% (11); adjusted for BMI, other covariates RR = 1.15 (1.08–1.22) (8) Rotating: RR = 1.60 (1.20–2.14), *I*^2^ 97.3% (5); adjusted for BMI, other covariates RR = 1.15 (1.06–1.25) (5)
Li et al. (20), China Meta-analysis	Permanent night (4) Evening (4) Rotating (5) Unspecified (3)	Type 2 diabetes (2) Diabetes mellitus (10) Diabetes death certificate Self-reported or medical/register report HbA1c	Adjustment (12)	Shift work: RR = 1.12 (1.07–1.17), *I*^2^ 38.9% (corrected for publication bias) (12) Night/Evening shift: RR = 1.19 (1.09–1.30), *I*^2^ 52.2% (4) Rotating shift RR = 1.11 (1.06–1.16), *I*^2^ 48.2% (5) Dose-response analyses: Shift work: RR = 1.07 (1.04–1.09), *I*^2^ 0% per 5-year exposure (2, ♀) Subgroups analyses: Shift work: RR = 1.21 (1.08–1.17) for ♀, *I*^2^ 46.9%/RR = 1.28 (1.16–1.42) for ♂ (1) Shift work: RR = 1.13 (1.07–1.19) for follow-up ≥10 years, *I*^2^ 49.5%/RR = 1.17 (1.10–1.24) for follow-up < 10 years (1)
Rosa et al. (15), Italy	Night shift (1)	Type 2 diabetes Register (1)	MD	Night shift: HR = 1.58 (1.25–1.99) (1)
Gao et al. (19), China Meta-analysis	Night shift (10) Rotating (4) Evening (2) Unspecified (5)	Type 2 Diabetes (21) FPG HbA1c OGTT Random plasma glucose	NA (4) Adjustments (17)	Shift work: RR = 1.10 (1.05–1.14), *I*^2^ 37.2% (21) Night shift: RR = 1.15 (1.08–1.24), *I*^2^ 60.7% (10) Rotating: RR = 1.08 (1.04–1.12), *I*^2^ 0% (4) Dose-response analyses (3, ♀): RR = 1.05 (1.03–1.07) per 5-year exposure of shift work; RR = 1.17 (1.11–1.24) for 15 years of shift work

(*n*), number of studies concerned; SW, shift work; FPG, Fasting plasma glucose; OGTT, Oral Glucose Tolerance Test; RPG, Random-plasma glucose; HbA1c, glycated hemoglobin MD, Missing Data; NA, non Adjusted.

### Lipid disorders

From the eleven systematic reviews assessed for eligibility, four met the criteria (Supplementary Figure A): three systematic reviews (12, 14, 21) and one meta-analysis (22). Six did not report any calculated risk and one focused on mechanistic hypotheses, which could potentially explain the link between shift work and lipid disorders.

The Proper et al. (14) and Dutheil et al. (22) papers reached a good level of quality according to AMSTAR-2 (Figure 1).

In total, eighty-five primary studies were included in the four systematic reviews. The systematic review with meta-analyses (22) encompassed about 50 and 60% of the primary studies analyzed by Esquirol et al. (12) and Proper et al. (14), respectively, and added forty new primary studies.

The four systematic reviews covered the period from 1976 to 2019 (Supplementary Table D). Around 231,500 participants mainly from the industry were included in these four reviews. Except for self-reported lipid measurement in one primary study, results of the others were based on blood measurements. Dutheil et al. conducted meta-analyses to explore the impact of night-shift work on several types of lipid disorders (22).

A high triglyceride level was the most reported change of lipid disorders in shift workers compared to day workers from the three systematic reviews (12, 14, 21), with a 12% risk excess estimated by Dutheil et al. (22). Results for total cholesterol, HDL-C and LDL-C were inconsistent, even though some authors supported an increased risk of hypercholesterolemia after exposure to 20-year shift work (12). With high heterogeneity, some mean differences of lipid levels according to different type of shift work were suggested by Dutheil et al., notably lower HDL-C among permanent night shift work and rotating night-shift work (22) (Table 2).

**Table 2:** Main results of systematic reviews focused on the link between shift work and lipid disorders.

**References, Country**	**Type - Shift work (*n*)**	**Assessment of outcomes (*n*)**	**Confounding factors (*n*)**	**Main results shift work vs. day work (*n*)**
Boggild and Knutsson (21), Nordic countries	Unspecified (16)	TC (16) TG (12) measurements HDL/LDL (3)	MD	TC (16): no difference TC level (10); higher TC level for SW or different organizations of SW (5); lower TC for male SW, but no difference for women (1) Significant changes between 3 and 20% in cholesterol (3) HDL-C and LDL-C: no difference (3) TG (12): No difference (8); higher TG for SW (4); higher values for counter clockwise than clockwise rotation (1)
Esquirol et al. (12), France	Permanent night (5) Rotating (18) Unspecified (2)	TC (13) TG (6) HDL (8) LDL (6) measurements, HighTG (6) LowHDL (6) HighTC (3) Self-reported highTC (1)	NA (8) Adjustments (15)	TC: Longitudinal studies: - no difference of Hypercholesterolemia (5); higher hypercholesterolemia in SW (2) - TC mean increase in SW (1) - TC level raised 14 years later ≥20/≥25/≥30/≥40%; OR = 1.16 (1.07–1.26)/1.16 (1.05–1.28)/1.11 (0.98–1.25)/1.30 (1.07–1.58) (2) - Exposure duration: a 5% risk of 20% increase TC for SW> = 20 years (1); increase of TC level with exposure duration for SW ♂ ≥30 y but not ♀ ≥30 y SW (1) TC: Cross-sectional studies: no difference of Hypercholesterolemia (5); higher Hypercholesterolemia in SW (1) HDL-C: no difference of hypoHDLemia (7); higher hypoHDLemia (5) LDL-C: no difference of LDLemia (5) TG: no difference of Hypertriglyceridemia (3); higher hypertriglyceridemia in SW (6)
Proper et al. (14), Netherlands	Permanent night (1) Rotating (8) Unspecified (3)	TC (7) TG (4) HDL (3) LDL (1) measurements, HighTG (3) LowHDL (3) HighTC (1) LDL/HDL (1)	NA (3) Adjustments (9)	TC: higher TC level (5); no difference of TC (5) HDL-C, LDL-C, TG: positive association (5); no difference (5)
Dutheil et al. (22), France Meta-analysis	Permanent night Rotating Unspecified	SMD or high level of TC, LDL, low level of HDL	NA for main results	TC increase: Only permanent night shift: SMD = 0.22 (0.01–0.42) *p* = 0.043, *I*^2^ 60.3% (4) Hypercholesterolemia: NS results HDL-C decrease: Permanent night shift: SMD = −0.16 (−0.32 to 0.00), *p* = 0.05, *I*^2^ 72.3%; Rotating 3 × 8 shift SMD = −0.10 (−0.17 to −0.02), *p* = 0.01, *I*^2^ 78.8%; Non-specified shift: SMD = −0.08 (−0.15 to −0.01), *p* = 0.027, *I*^2^ 81.7%; HypoHDLemia: NS results LDL-C increase: NS results TG increase: permanent night shift SMD = 0.18 (0.03–0.33), *p* = 0.017, *I*^2^ 73.8% (7); Rotating 3 × 8 shift SMD = 0.09 (0.03–0.16), *p* = 0.004, *I*^2^ 73.6% (21) Rotating 2 × 12 shift: SMD = 0.07 (0.01–0.13), *p* = 0.017, 31.6% (11); Unspecified shift SMD = 0.11 (0.03–0.18), *p* = 0.004, *I*^2^ 80.9% (12) Hypertriglyceridemia: OR = 1.12 (1.01–1.23), *p* < 0.001, *I*^2^ 55.2%

(*n*), number of studies concerned; SW, shift work; HDL-C, High-Density Lipoprotein Cholesterol; LDL-C, Low-Density Lipoprotein Cholesterol; TC, Total Cholesterol; TG, Total Triglycerides; SMD, standardized mean difference; MD, Missing Data; NA, Non Adjusted.

### Being overweight

Nine systematic reviews were identified on the consequences of night-shift work on weight gain during working life (12, 14, 23–29) (Supplementary Figure A). Among them, four proposed results of meta-analyses (25–27, 29).

The quality of the systematic reviews was highest for most recent systematic reviews with meta-analysis (27, 29) (Figure 1).

Two meta-analyses published in 2018 included 27 and 28 primary studies respectively, with an overlap of 65% (25, 27). For nurses, four out of seven primary studies considered in Saulle's meta-analysis (26) were also included in one of Zhang's (29).

The total number of participants for these systematic reviews varied from 11,537 (28) to 311,334 (25). Primary studies included were published between 1986 and 2017 (Supplementary Table E). The weight increase was dealt with by using either the classic threshold of BMI (<25/25–30/>30 kg/m^2^ to distinguish a normal weight, being overweight or being obese, respectively) or the threshold of waist circumference adapted to gender (≥80/≥94 or 88 cm) to determine abdominal adiposity, or weight changes during a defined period. Some authors distinguished being obese and being overweight, some of them considered both in analyses. Moreover, some authors reported results concerning overall shift work and other authors reported more specific results for rotating shift work and night shift work.

From meta-analyses, when overall shift workers were considered, an increased significant risk of being overweight ranging from 1.25 (25) to 1.32 (27) was observed, while it was assessed as 0.95 (0.24–1.14) among nurses (29). For obesity, Liu et al. and Sun et al. confirmed a significant excess risk ranging from 17% (25) and 25% (27) for shift work. Among nurses, based on four studies, Saulle et al. did not highlight any significant risk of obesity (26) whilst 2 years later, the results based on eight studies confirmed an excess risk of 12% (29). When subgroup analyses were undertaken, rotating shift work increased the risk of being overweight to 21% and the risk raised to 38% for night-shift work (25). A statistically significant increase of obesity risk was observed for rotating shift work (18%) and for night work (5%) (25). A dose-response effect was highlighted in primary studies included in Sun et al. (27) and Esquirol et al.'s (12) reviews. The density (number of nights per month) and duration of exposure increased the risk of being overweight/obesity (27): the threshold was not exactly determined but a duration of exposure of over 6 years has been put forward and a BMI gain of 0.24 kg/m^2^ was estimated for each year of night work (Table 3).

**Table 3:** Main results of systematic reviews focused on the link between shift work and being overweight.

**References, Country**	**Type -Shift work (*n*)**	**Assessment of outcomes (*n*)**	**Confounding factors (*n*)**	**Main results shift work vs. day work (** ***n*** **)**	
Antunes et al. (24), Brazil	Night shift (1) Rotating (3) Unspecified (5)	BMI (8) BMI ≥25 kg/m^2^ (1) WHR (5)	Adjustments (4) MD (5)	**Significant higher weight (9):** Shift work: higher BMI (♂, 1); increase BMI and WHR (♂, 1) Night shift: higher BMI (♂, 1); Rotating: increase BMI and WHR (♂, 1) 3-shift: higher WHR (♂, 1)	**Shift duration:** correlation with BMI (1); ♀ ≥ 30 yo, higher WHR (1); increase BMI and WHR (1) Rotating duration: correlation *r* = 0.19, *p* < 0.05 with BMI (♂, 1) No significant weight difference (1): 3-shift: no difference BMI (♂, 1)
Esquirol et al. (12), France	Permanent night (3) Rotating (18) Unspecified (1)	Weight change (2) BMI (15); BMI ≥ 25 or ≥ 30 kg/m^2^ (4) WC (3); WC ≥ 80 or ≥ 94 cm (2) WHR (6); WHR > 0.9 (1)	NA (12) Adjustments (10)	**Threshold values of weight (5):** Shift work: prevalence obesity, 9.6% vs. 8.5%, *p* < 0.004 (♀, 1) Shift work: BMI ≥30 kg/m^2^, ♀ OR = 1.39 (1.25–1.55); ♂ OR = 1.44 (1.27–1.64) (1) Rotating: OR = 1.12 (0.88–1.42) for WC ≥ 94 cm (♂, 1) Rotating: prevalence of obesity NS; WHR > 0.9 OR = 1.19 (0.92–1.56) (♂, 1) 12 h-permanent night: BMI ≥25 kg/m^2^, OR = 2.7 (1.6–4.5); WC ≥80 cm OR = 2.9 (1.7–5.1) (♀, 1) **Continuous variables (1):** Shift work: higher WC or BMI (9); Rotating: lower BMI (♂, 2) Shift work: no significant difference (♂, 2)	**Duration (5):** Rotating: BMI increase, 0.89 vs. 0.62 kg/m^2^, *p* < 0.05 after 10-year exposure (♂, 1) Rotating: correlation *r* = 0.19, *p* < 0.05 with BMI (♂, 1) Night: weight gain since starting the job on current shift, + 4.3 vs. 0.9 kg, *p* < 0.02 (♀, 1) Shift work: significant 1-year follow-up decrease of BMI (1) Rotating: BMI no difference (1)
van Drongelen et al. (28), The Netherlands	Permanent night (2) Rotating (5) Unspecified (2)	BMI change (4) Weight change (4) WC change (2)	NA (2) Adjustments (6)	**BMI change:** significant effect (3) Shift work: 10-year FU ΔBMI, mean: 0.89 vs. 0.62, *p* = 0.001 (1) Rotating: 1-year FU ΔBMI, %: 0.63 vs. 0.40, *p* = 0.002 (1) Rotating: 1-year FU ΔBMI, mean: −0.33 vs. 0.07, *p* = 0.01 (1) **BMI change**: NS effect (1): Shift work: 5-year FU ΔBMI: −0.05 (95% CI −0.024 to 0.15), *p* = 0.63 (1) **Weight change:** significant effect (2): Shift work: 1-year FU Δweight, mean: −1.02 vs. 0.28 kg, *p* = 0.007 (1) Permanent night: FU unknown: Δweight, mean: 4.4 vs. 0.7 kg, *p* = 0.008 (1)	**Weight change:** NS effect (2): Shift work: 5-year FU, Correlation coefficient, *p* = NS (1) Rotating: 1-year FU Δ weight mean: 3-day rotating 0.73, 5-day rotating shift 0.89 kg vs. 1.02 kg, *p* = NS (1) **WC change:** NS effect (2): Rotating: 1-year FU ΔWHR mean:−0.0102 vs. −0.0053, *p* = 0.25 (1) Rotating: 1.5-year FU ΔWC−0.1 vs. +0,2 cm, *p* = NS (1)
Amani and Gill (23), Iran	Night shift (1) Rotating (4) Unspecified (4)	BMI ≥ 30 or ≥ 25 or ≥ 27 kg/m^2^ (5); Weight change (1); BMI (4)	NA (4) Adjustments (5)	Significant higher weight (7): Shift work: higher BMI (♂, 2) Shift work: overweight OR = 1.60 (1.28–2.06) (♀ 1); OR = 1.54 (1.06–2.25) (♀, 1) Shift work: higher obesity prevalence (♂, 1) Shift work: obesity significant OR = 1.4 (1)	Rotating duration: significant correlation *r* = 0.19, *p* < 0.05 with BMI (♂, 1) Night shift: overweight OR = 3.3 (1.3–8.2); 5-year weight gain > 7 kg: OR = 2.9 (1.2–6.9) (♀, 1) 3-shift: higher WHR (♂, 1) Shift work: significant 1-year follow-up decrease of BMI (1) No significant weight difference (2)
Proper et al. (14), Netherlands	Night shift (5) Rotating (12) Unspecified (5)	Weight change (3) BMI (10); BMI ≥25 or ≥ 30 kg/m^2^ (5) WC (2); WC ≥ 80 or ≥ 94 cm (2) WHR (2)	NA(2) Adjustments (17)	Shift work: BMI or weight: positive relation (10); negative relation (1); no relation (4) WC: positive relation (2); negative relation (0); no relation (2) Obesity: positive relation (7); negative relation (0); no relation (3)	
Liu et al. (25), China Meta-analysis	Night shift (5) Rotating shift (18) Unspecified (4)	BMI ≥ 25 or ≥ 23 kg/m^2^ (11); WC ≥ 94 cm (1) BMI ≥ 25 or ≥ 30 kg/m^2^ (23); Total fat % (1); ICD-10 (1)	Adjustments (27)	**Overweight:** Shift work: RR = 1.25 (1.08–1.44), *I*^2^ 80.7% (12) Rotating shift: RR = 1.21 (1.02–1.43), *I*^2^ 73.2% (8) Night shift: RR = 1.38 (1.06–1.80), *I*^2^ 28.5% (5) Shift work: RR = 1.14 (0.97–1.35), *I*^2^ 84.3% (♀, 6); RR = 1.46 (0.98–2.15), *I*^2^ 51.2% (♂, 5)	**Obesity:** Shift work: RR = 1.17 (1.12–1.22), *I*^2^ 92.2% (23) Rotating shift: RR = 1.18 (1.08–1.29), *I*^2^ 91.7% (17) Night shift: RR = 1.05 (1.00–1.10), *I*^2^ 81.0% (7) Shift work: RR = 1.19 (1.06–1.34), *I*^2^ 90.8% (♀, 13)/RR = 1.27 (1.10–1.46), *I*^2^ 81.9% (♂, 9)
Saulle et al. (26), Italy Meta-analysis	Unspecified (7)	BMI> 25 or > 30 kg/m^2^ (4) BMI (2) WC (1)	MD	Shift Work: BMI >30 kg/m^2^: OR = 1.00 (0.66–1.50), *I*^2^ 74.5% (nurses) (4)	
Sun et al. (27), China Meta-analysis	Night shift (15) Rotating (16) Unspecified (4)	Weight/BMI gain (2) BMI ≥ 25 or 25–29.9 or ≥30 kg/m^2^ (28) WC or WHR (9)	NA (3) Adjustments (25)	**For obesity/overweight** Overall shift: OR = 1.23 (1.17–1.29), *I*^2^ 90.7% (28) Rotating: OR 1.14 (1.05–1.23), *I*^2^ 67.5% (15) Permanent night: OR = 1.43 (1.19–1.71), *I*^2^ 70.8% (7) Shift work: BMI ≥25 kg/m^2^: OR = 1.32 (1.15–1.51), *I*^2^ 72.9% (14) BMI ≥30 kg/m^2^: OR = 1.25 (1.11–1.45), *I*^2^ 95.9% (11)	Dose-response (frequency and duration) (4) Trend toward obesity risk with the increase of night shifts per month (2) Night shift: BMI ≥30 kg/m^2^: ≥21 nights/month OR = 3.42 (1.95–6.03) (1); ≥8 nights/month: OR = 3.9 (1.5–9.9) (1) Night shift: WHR ≥0.85, ≥8 nights/month OR = 2.4 (1.2–4.9) (1) Increase of BMI per year of night: 0.24 (0.12–0.37) kg/m^2^ (1)
Zhang et al. (29), China Meta-analysis	Night shift (5) Shift work (6)	BMI ≥25 or ≥30 or >30 kg/m^2^ (10) WC ≥80 cm or WC ≥88 cm (2)	MD	**For obesity/overweight** Overall shift: OR = 1.05 (0.97–1.14), *I*^2^ 0.% (11) Shift work: OR = 0.99 (0.59–1.38), *I*^2^ 52.9% (6) Night shift: OR = 1.12 (1.03–1.21), *I*^2^ 97.2% (5)	Shift work: BMI ≥25 kg/m^2^ OR = 0.95 (0.24–1.14), *I*^2^ 81.8% (2) Shift work: BMI ≥30 kg/m^2^ OR = 1.12 (1.03–1.20), *I*^2^ 95.1% (8) Shift work: WC ≥80 cm OR = 3.21 (1.29–7.98) (1) Shift work, ♀: OR = 1.09 (0.84–1.35), *I*^2^ 96.1% (7)

(*n*), number of studies concerned; SW, shift work; BMI, Body Mass Index; WHR, Waist Hip Ratio; WC, Waist circumference; NS, Non Significant MD, Missing Data; FU, Follow-up; NA, Non Adjusted.

### Hypertension

After the initial research identifying 121 articles, 108 were excluded based on title and abstract. Of the remaining 13 reviews, nine were excluded in full-text screening. Therefore, four reviews were eligible papers: two systematic reviews with meta-analysis (30, 31), two without meta-analysis (12, 14) (Supplementary Figure A).

The quality of the included systematic reviews, assessed using AMSTAR-2 (Figure 1), was high for the latest reviews with meta-analysis (30, 31), with the highest level for Gamboa Madeira et al. in 2021 (30). In most reviews, the inclusion and exclusion criteria (12, 14, 30, 31), the study selection and the data extraction (14, 30, 31) were clearly stated. The search strategies were incomplete in the four systematic reviews and the included primary studies were partially described. The risk of bias was assessed in the primary studies of most reviews (14, 30, 31).

Only two primary studies overlapped in Manohar and Gamboa Madeira's systematic reviews with meta-analyses.

Supplementary Table F provides an overview of the characteristics of the included systematic reviews. The four systematic reviews were published from 2011 to 2021, gathering primary studies published between 1986 and 2015. These primary studies were conducted in Africa, Americas, Asia, and Europe. The number of studies included in each systematic review ranged from 19 to 45. Finally, 81 unique primary studies addressed the question about shift work and hypertension. The risk of hypertension was examined by using either referenced threshold values or change in systolic and diastolic blood pressure.

An elevated risk of hypertension for rotating shift work with or without night shift was observed and estimated at 1.34 (1.08–1.67) (31) and 1.26 (0.94–1.68) (30). Gamboa Madeira et al. estimated a significantly positive magnitude of blood pressure (BP) change for: (1) permanent night shifts (increased systolic BP (SBP) of 2.52/diastolic BP (BP) of 1.76 mmHg); (2) rotating shifts with nights (increased SBP of 0.65 mmHg); (3) rotating shifts without nights (increased SBP of 1.28 mmHg) in comparison to day workers (30) (Table 4).

**Table 4:** Main results of systematic reviews focused on the link between shift work and hypertension.

**References, Country**	**Type - Shift work (*n*)**	**Assessment of outcomes (*n*)**	**Confounding** ** factors (*n*)**	**Main results shift work vs. day work (*n*)**
Esquirol et al. (12), France	Permanent night (5) Rotating (33) Evening (2)	HTN (13) BP measures (14) 24-h Ambulatory BP (5) HTN history (2)	NA (15) Adjustments (19)	**Longitudinal studies**: Shift work: HTN OR = 1.10 (1.01–1.20) (1); progression from mild to severe HTN OR = 1.23 (1.05–1.44) (1) Shift work: raised systolic or diastolic BP: significantly (3), no difference (7) Sub-group analyses: - Age and Shift work: 30–39 yo (NS); 40–49 yo OR = 1.62 (1.17–2.24); 50–59 yo (NS) (1) - Duration and shift work: ♂ ≥30 yo: SW duration positively associated with SBP (*p* < 0.05); ♀ <30 yo: SW duration inversely associated with DBP (*p* < 0.05) (1); SBP and DBP associated with duration of SW (*p* < 0.05); BP fell morning to afternoon to night (*p* = 0.03) (1) - Shift work after 1-year follow-up: NS change in BP (1)
Proper et al. (14), Netherlands	Permanent night (1) Rotating (13) Unspecified (5)	HTN (11) BP mesures (7) Self-reported (3) Register (1)	NA (2) Adjustments (15) Unspecified (2)	Shift work: significant increased risk of HTN (9); NS (6) Permanent night: HTN OR = 0.9 (0.6–1.2) (1) Shift work: significant elevated risk of increased BP (1); NS (6)
Manohar et al. (31), USA Meta-analysis	Rotating (18) Permanent night (4) Irregular (2) Unspecified (4)	HTN (18) Self-reported BP (7) BP measures (1) MD (1)	NA (2) Adjustments (25)	Shift work: cohort studies: HTN pooled OR = 1.31 (1.07–1.60), *I*^2^ 90%; cross-sectional studies: HTN pooled OR = 1.10 (1.00–1.20), *I*^2^ 85% Rotating: cohort studies: HTN pooled OR = 1.34 (1.08–1.67), *I*^2^ 91% Permanent night: cross sectional studies: HTN pooled OR = 1.07 (0.85–1.35), *I*^2^ 83% Sub-group analyses: Rotating: cohort studies HTN ♂ pooled OR = 1.21 (1.04–1.40), *I*^2^ 63%; HTN ♀ pooled OR = 1.01 (0.70–1.44), *I*^2^ 14% Permanent night: cross-sectional studies ♀ pooled OR = 1.07 (0.88–1.30), *I*^2^ 66%
Gamboa Madeira et al. (31), Portugal Meta-analysis	Permanent night (14) Rotating with night (30) without night (4) Unspecified (8)	HTN (14) BP measures (41)	NA (32) Adjustements (13)	Rotating with night: HTN pooled OR = 1.26 (0.94–1.68), *I*^2^ 90% (8) Rotating without night: HTN OR = 1.00 (0.88–1.15) (1) Permanent night: HTN pooled OR = 1.00 (0.80–1.27), *I*^2^ 35% (6) Permanent night: increase mean difference SBP = 2.52 mmHg (0.75–4.29), *I*^2^ 91% (12); DBP = 1.76 mmHg (0.41–3.12), *I*^2^ 93% (12) Rotating with night: increase mean difference SBP = 0.65 mmHg (0.07–1.22), *I*^2^ 69% (28); DBP = 0.12 mmHg (−0.31 to 0.54), *I*^2^ 65% (25) Rotating without night: increase mean difference SBP = 1.28 mmHg (0.18–2.39), *I*^2^ 93% (4); DBP = 0.60 mHg (−0.24 to 1.43), *I*^2^ 92% (4)

(*n*), number of studies concerned; HTN, Hypertension; SBP and DBP, Systolic and Diastolic Blood Pressure; MD, Missing Data; NA, Non adjusted; NS, Non significant.

### Smoking habits

Out of the 60 articles identified in the initial research, 52 were excluded based on title and abstract (Supplementary Figure A). Of the eight reviews potentially eligible for inclusion, six were excluded in full-text screening mainly due to the absence of estimated risk between shift work and smoking habits. Therefore, two systematic reviews without meta-analysis were included (21, 32).

Zhao's systematic review fulfilled most of the AMSTAR-2 criteria (Figure 1).

The two systematic reviews covered 23 primary studies (17 cross-sectional and six prospective) published from 1976 to 2004, without overlapped studies (Supplementary Table G).

Participant details (sex, age, and occupation), countries, and type of shift work were missing in Boggild and Knutsson's (21). The seven primary studies included in Zhao's were conducted in Europe (3) and Asia (1), among different types of shift workers in various occupational sectors (32).

Fifty percentage of the 23 primary studies reported a significantly higher tobacco consumption in shift workers in comparison to day workers (21, 32), with a potential effect during the first year of shift work (32) (Table 5).

**Table 5:** Main results of systematic reviews focused on the link between shift work and smoking habits.

**References, Country**	**Type - Shift** ** work (*n*)**	**Assessment of outcomes (*n*)**	**Confounding factors (*n*)**	**Main results shift work vs. day work (*n*)**
Boggild and Knutsson (21), Nordic countries	Unspecified (16)	Smokers, % (14) Cigarettes/day (2)	NA	Tobacco consumption: - Cross sectional studies: Significantly higher (6), lower (1), no difference (5) - Prospective studies: at baseline of studies: higher (2); After 6-months follow-up, no difference of number of new smokers and no change habits (1)
Zhao and Turner (32), Australia	Permanent night (2) Rotating (2) Evening (1) Unspecified (3)	Smokers, % (5) Cigarettes/day (2)	Adjustments (1) MD (6)	- Shift work: current smokers OR = 1.3 (1.1–1.6) (1) - Rotating shift: current smokers: 40 vs. 34.3%, *p* = 0.058 (1); % of every day smokers: NS (1) - Permanent night: more likely to smoke and smoked significantly (*p* < 0.01) more cigarettes/day (1) - Shift work: significantly higher tobacco consumption, *p* = 0.027 (1) - Shift work: after 1-year follow-up, significantly higher number of cigarettes/day (1)

(*n*), Number of studies concerned; MD, Missing Data; NA, Non Adjusted; NS, Non Significant.

### Occupational psychosocial stressors

After reading the full text of 20 reviews, three were finally considered for this purpose (Supplementry Figure A): one systematic review (33) and two systematic reviews with meta-analyses (34, 35).

The quality criteria were met for all the considered reviews (56–77% of criteria met), especially the one of Taghighi (Figure 1). Item no. 4 (comprehensive literature search) was partially met. As previously underlined, the criteria related to an *a priori* registered protocol (item no. 2), to the justification of excluded studies (item no. 7) and to the reporting on the sources of funding for the studies included (item no. 10) were almost never provided.

Supplementary Table H summarized the main characteristics of the three selected reviews (33–35).

Firstly, Taghighi et al. focused on the psychological functioning and resilience of nurses who carry out shift work. The authors selected 37 primary qualitative and quantitative studies, with comparison to day workers (17 studies) and between different types of shift work (20 studies) (33). Most were quantitative and cross-sectional studies. Psychological functioning was measured using different outcomes: (a) general psychological wellbeing or quality of life, (b) depression, anxiety or stress and (c) job satisfaction or burnout. The synthesis of the different results revealed that shift work seemed to limit social life and to be associated with work/family conflict, low levels of wellbeing, poor mental health and high levels of burnout. However, the authors could not definitively come to a conclusion, because these significantly negative effects of shift work were only observed in several studies and contrasted based on the different types of night-shift work.

Secondly, based on workers from different job sectors, the two systematic reviews with meta-analyses examined the impact of night-shift work on mental health defined as depression or psychological distress using standardized questionnaires or psychiatric diagnoses (34, 35). Angerer et al. considered 11 prospective studies published between 1989 and 2015 (34). Zhao et al. mixed cross-sectional (*n* = 22) and longitudinal (*n* = 11) primary studies published during 2002–2017 and included mainly shift workers using surveys from the general working population (35).

From the five longitudinal studies, Angerer et al. reported a non-statistically significant elevated meta RR for depression of 1.42 (0.92–2.19) for shift workers vs. day workers. The results differentiated according to the type of working populations: two out of the three reports from the same study in nurses did not confirm an increased risk of depression in those who work shifts, whereas four out of the six studies conducted in the general working population suggested such relationship (34). This conclusion was in line with results of Zhao et al. (35): with shift work defined as a broad binary indicator and based on four longitudinal studies, the authors revealed an excess risk of mental health problems in shift workers compared to non-shift workers [meta OR = 1.32 (1.01–1.73)]. The authors reported inconclusive results about gender differences, even if some studies provided evidence of more vulnerability to shift work in females. Finally, when considering shift work as night or evening work, only two out of six cross-sectional and three out of six longitudinal studies showed a significant association between shift work and poor mental health (35) (Table 6).

**Table 6:** Main results of systematic reviews focused on the link between shift work and occupational psychosocial stressors.

**References, Country**	**Type - Shift work (*n*)**	**Assessment of outcomes (*n*)**	**Confounding factors (*n*)**	**Main results shift work vs. day work (*n*)**
Angerer et al. (34), Germany Meta-analysis	Permanent night (5) Rotating (11) Irregular (2)	**Depression:** GHQ-12; HADS; COPSOQ; Prescriptions of antidepressants; Psychiatric interview; ICD	Adjustments (11)	**Depression**: Shift work: pooled OR = 1.42 (0.92–2.19), *I*^2^ 74.4% (5); Shift work with autonomy in their schedule planning: lower risk of depressive symptoms (1)
Tahghighi et al. (33), Australia	Shift work (5) Rotating (28) Permanent night (17)	**Wellbeing/Quality of Life**: 1 item measure of wellbeing; Scale of the negative effects of work time; Conflict between work and family rating scale; Chinese health questionnaire 12-item; WHOQOL-BREF **Job satisfaction:** Job satisfaction scales; Standard shift work index questionnaire; Job, family and life satisfaction scale **Burnout:** MBI; CBI; Job stress questionnaire from the Korean occupational stress scale **Depression, Anxiety and Stress:** NSS; BDI-II; CES-D; PHQ-9; HAD-S; Taiwan nurse stress checklist; STAI-Y; Profile of mood states; GHQ-12 **Resilience and Coping**: Coping questionnaire; Hardiness and resilience Scales	MD	**Wellbeing/Quality of Life (8)**: Association between Shift work and poor quality of life and low psychological wellbeing, dependent on the type of shifts **Job satisfaction/Burnout (11):** Higher rates of burnout in the shift workers (5); Impact of different types of shift work on job satisfaction and burnout: mixed results (6) **Depression, Anxiety and Stress (17):** inconsistent results **Resilience and Coping (9):** inconsistent results
Zhao et al. (35), Australia Meta-analysis	Shift work (12) Rotating (5) Permanent night or evening (12) Irregular (14)	**General mental health**: Kessler-6 (4); SF-36/SF-12 (7); GHQ-12 (5); ILfeld psychiatric symptoms index (3) **Depression:** CES-D (8); BDI (3); WHO wellbeing scale (3); NHP (1); CIDI-SF (1); PHQ-9 (1); HAD-S (1); STAI-Y (1)	NA (6) Adjustments (27)	**Mental health problems:** Shift work: OR = 1.32 (1.01–1.73), *I*^2^ 63% (4); Night/Evening work: significant association (5/12)

(*n*), number of studies concerned; MD, missing data; NA, non adjusted.

### Sedentariness

Until September 2021, none of the 122 reviews addressed the relationship between shift work and sedentariness after applying the eligibility criteria (Supplementary Figure A). However, when updating to September 2022, two systematic reviews were retrieved: one with meta-analysis (36) and one without meta-analysis (37) (Supplementary Table I).

The quality of the included systematic reviews was moderate. While the inclusion and exclusion criteria, the study selection, and the data extraction were well-described, the risk of bias was not assessed.

Only one primary study was included in both systematic reviews.

The two systematic reviews gathered 52 primary studies (49 in Monnaatsie et al. and three in Crowther et al.) among workers from different job sectors covering the period 2001–2021. The total number of participants for these systematic reviews varied from 29,701 to 310,710.

In the meta-analysis, Monnaatsie et al. studied the prevalence of meeting physical activity guidelines, time spent in moderate-to-vigorous physical activity and in sedentary behavior (36). No significant difference was found in the prevalence of meeting physical activity guidelines and for the time spent in moderate-to-vigorous physical activity among shift-workers compared to non-shift workers. Time spent in sedentary behavior was lower in shift workers than non-shift workers [SMD = −0.2 (−0.50; −0.001)] (Table 7).

**Table 7:** Main results of systematic reviews focused on the link between shift work and sedentariness.

**References, Country**	**Type - Shift work (*n*)**	**Assessment of outcomes (*n*)**	**Confounding factors (*n*)**	**Main results shift work vs. day work (*n*)**
Monnaatsie et al. (36), Australia Meta-analysis	Shift work (26) Rotating (13) Night (25)	IPAQ (9) Other questionnaire (22) Self-report (3) Actigraph (14) Accelerometer (3) Calorie counter (1)	MD (49)	Meeting physical activity guidelines: shift work: 8–63.4%/non-shift work 3–67.7%; OR = 0.84 (0.68–1.03), *I*^2^ 93.3% (12) Time spent in physical activity/day: shift work 13.2%/non-shift work 14.2%, NS; SMD = −0.1 (−0.4 to 0.2), *I*^2^ 98.8% (12) Time spent in sedentary behavior/day: shift work 37.0%/non-shift work 39.0%; SMD = −0.2 (−0.5 to −0.001) (7)
Crowther et al. (37), Australia	Permanent night (2) Rotating (3)	Questionnaire (3)	NA (3)	Shift work: 14–19% inactive (1) Significant increase physical inactivity over time (1) No significant change in physical activity over time (1)

(*n*), number of studies concerned; MD, missing data; NA, non adjusted; NS, non significant.

## Discussion

### Main findings

A comprehensive synthesis of the main findings from the 33 included systematic reviews, structured around the type of cardiovascular risk factors was conducted. From this umbrella review, which aimed to evaluate the existing evidence on the effect of night-shift work and its subtypes on cardiovascular risk factors, the key findings can be displayed as two categories: well-established results and those that require further research (Graphical Abstract).

The results asserted an excess risk of diabetes at around 10%, regardless of the type of night-shift work, with a suspected dose-response effect in women (increased risk of 5–7% every 5 years). A stated excess risk of being overweight at around 25% was also reported for overall shift workers; and it could reach 38% among night-shift workers. When it comes to obesity, elevated risks estimated at 5% for night-shift workers and at 18% for rotating shift workers were observed, with an increase of this risk based on the density and duration of exposure. An excess risk of hypertension was estimated at around 30% when the broad definition of shift work was considered and when night periods were included in rotating shift work.

Literature provided inconsistent results for the relationship between lipid disorders (total cholesterol, HDL-C, LDL-C) and night-shift work, with a probable variation according to the type of shift work (lower HDL-C level among permanent and rotating night-shift workers). Although no clear conclusion can be drawn, shift workers appeared to be more likely to smoke. The relationship between shift work and occupational psychosocial stressors was scarcely explored in available studies. However, the consequences of night-shift work on mental health disorders (depression, in particular) were further investigated, with an increased risk of depression at 32–42%. Finally, the sedentariness was scarcely considered in systematic reviews, which prevents any firm conclusions.

One previous umbrella review, conducted on systematic reviews with meta-analyses published until April 2019, aimed to assess the relationship between shift work or long working hours and various chronic health conditions (38). Only three cardiovascular risk factors of interest were considered in this umbrella review. The authors found very low-grade evidence concerning the relationship between shift work and diabetes mellitus (based on two systematic reviews with meta-analysis), obesity (four systematic reviews with meta-analysis) and hypertension (one systematic review with meta-analysis). Another umbrella review conducted on systematic reviews with or without meta-analyses published until April 2020, aimed not only to summarize the evidence but also to assess the validity of the associations of shift work with different health outcomes (39). Diabetes mellitus incidence was the only health outcome in common with our umbrella review. Based only on the results of Li et al. meta-analysis (20), Wu et al. concluded to highly suggestive evidence for association between shift work and diabetes mellitus incidence (39). Our umbrella review specifically focused on cardiovascular risk factors, retained a higher number of systematic reviews with or without meta-analyses, and deeply investigated the specific effect of night-shift work and its different subtypes (permanent or rotating). Thus, considering five systematic reviews (12–16) and four meta-analyses (17–20) reinforced the evidence of the association between night-shift work and diabetes, and provided a more comprehensive and detailed overview of cardiovascular risk profile of night-shift work.

### Assessment of night-shift work

In recent years, the primary studies tended to progress on a homogeneous definition of night-shift work. However, some reviews included in this umbrella highlighted the difficulties to compare results across studies due to inconsistent definitions of night-shift work (mixed rotating, irregular, evening, unspecified) (20, 25, 27, 29). The lack of detailed characteristics of night-shift work was also observed. Few studies assessed exposure parameters such as cumulative duration of exposure of night work alongside working life, average number of night shifts per month, number of consecutive nights per shift period and direction of rotation (clockwise and counter clockwise). Therefore, the dose-response effect cannot be determined.

Information on work schedules were obtained by different sources in the primary studies included in the reviews (14, 17–19). Data of work schedules were reported from the workers through self-administered questionnaires or from other sources such as payment records, employment records, or a list of job titles and workplace characteristics. In the case of self-administered questionnaires, several studies assessed exposure to shift work based on simple questions such as, “do you do shift work?” or “ever worked a night shift?” Other studies sought to distinguish permanent night shift from rotating night shift by asking the following question: “do you normally work (a) day, (b) evening, (c) night or (d) rotating shifts?”

In 2011, the IARC Working Group (cancer research) published recommendations to improve exposure to shift work in a consensus report (40). The authors notably pointed out the need to consider at least 3 h of work between midnight and 5 a.m. as a preliminary criterion in the definition of night work.

To our knowledge, the effects of former night-shift work have not been studied as a specific topic in systematic reviews. It may be worth considering in new synthesis works.

### Assessment of outcomes

As is usually done, the cardiovascular risk factors were reported from self-questionnaires, medical reports or clinical and biological measurements used as continuous values or according to established referenced thresholds.

With regards to diabetes, since all primary studies included in the reviews were conducted in adults, it could be assumed that diabetes was mostly type 2. Apart from two systematic reviews (15, 19), diabetes was not clearly specified as type 2, in particular when self-reports or death certificates were used. Moreover, when biological markers were used, the diagnosis of diabetes was based on several tests including glycaemia, HbA1c, OGTT, and random plasma glucose. The different definitions of the diabetes outcome may have introduced heterogeneity across the studies. However, the relationship was confirmed when Gan et al. conducted a subgroup analysis by restricting to studies that specified the type of outcome as type 2 diabetes (18).

With regards to being overweight/obesity, although this has been little explored in primary studies, the relationships between shift work and waist circumference were consistent with those observed when BMI was used (an increased risk of being overweight/obesity in night-shift workers). Moreover, some authors explored the time-varying weight gain, but this was done insufficiently to be able to determine the exposure duration threshold.

In addition to the 30% excess risk of hypertension observed when considering the referenced thresholds, mean differences of BP were used to explore the effect of night-shift work in Gambao Madeira's meta-analysis (30). A significant increase of a pooled mean difference of SBP was observed among permanent night workers (2.52 mmHg) and workers in rotating shift with nights (0.65 mmHg) compared to day workers. It is well-known that reductions in usual SBP levels of only 2 mmHg result in a 7–10% decrease of cardiovascular mortality in middle-aged people (41). In addition, given the BP nycthemeral cycle, the hour of BP measurement on the 24 h-period is important to mention for shift workers in the systematic reviews.

While significant mean differences were observed for the levels of triglyceride and HDL-C between night-shift workers and day workers, the results about lipid disorders estimated by referenced thresholds, were inconsistent in the systematic reviews.

Some of these cardiovascular risk factors are components of the metabolic syndrome (MetS). Two meta-analyses assessed significant increased risks of MetS, estimated at 57% for those exposed to night shift work, 31% for rotating shift workers and 28% for permanent night workers (42, 43). From a prevention point of view, knowledge on constitutive elements is more informative than the ultimate outcome.

Monnaatsie et al. reported similar levels of total physical activity among shift and day workers. They assumed that shift workers might report higher level of occupational physical activity and day workers higher level of leisure-time physical activity (36). Nevertheless, to clarify in particular the role of occupational physical activity, further research is needed.

In relation to the psychosocial stress pathway, the three systematic reviews selected in this umbrella review aimed to determine the relationship between night-shift work and psychological functioning in nurses (33) or in the working population in general (34, 35). Most of psychological outcomes reported in these systematic reviews were assessed with proxies of stress (i.e., psychological wellbeing, quality of life) or focused on consequences of stress (depression, burnout, job satisfaction), rather than with an assessment of occupational psychosocial stressors. Few authors reported that night workers had less autonomy and more conflict at work than day workers (44), while others observed that permanent night workers were more often satisfied with their co-workers and autonomy at work, although were more often confronted with workplace violence (45). Recently, Tucker et al. suggested that, despite notable differences in psychosocial working conditions between night and day workers, chronic disruption of circadian rhythms and sleep may play a more important role than psychosocial working conditions in explaining the observed significant health effects (symptoms of depression in men and short-term sick leave in women) (46). Thus, the psychosocial stress pathway requires further studies, focusing on occupational psychosocial stressors.

### Mechanisms

The pathophysiological mechanisms to explain the associations between shift work and cardiovascular risk factors are based on several complex and interrelated pathways. One most documented explanatory mechanism concerns a direct effect of the unusual schedule pattern, to which shift workers are subjected, on the internal hypothalamic clock that manages the alternation of periods of wakefulness and sleep and secondary internal clocks (i.e., located in the heart, adipose tissue, kidney, pancreas, and liver). These auto-regulated clocks at central and peripheral levels act through expression of many genetic factors, which determine the circadian rhythm of insulin secretion, carbohydrate, lipid metabolism, and adipogenesis. As well-demonstrated, the consequences of shift work on sleep are well-established, in particular in terms of reduction of sleep duration and quality (47). The misalignment of sleep and awake periods leads to sleep disturbances such as higher frequency of sleepiness, difficulties falling asleep and recovery sleep. More and more evidence is provided on the associations between sleep disorders and hypertension, autonomic dysregulation, metabolic disorders (48). In 2019, the American college of cardiology/American heart association promoted sleep hygiene to prevent cardiovascular diseases (49).

In addition to these internal circadian rhythms, food intake constitutes a well-known external environmental synchroniser. The misalignment induced by eating during the night is a major assumption advanced to explain the metabolism troubles encountered in night-shift or rotating shift workers. As demonstrated, the total 24-h energy intake did not differ significantly between shift, permanent or rotating workers and day workers (50), although any conclusion could be asserted concerning the macronutrient intake. The redistribution of energy intake and the eating behavior changes, pointed out a main effect of shift work on chrono-dietetic.

Psychological stress results from expression of stressors and notably occupational stressors, which can lead to, with an individual variability, mental health problems such as depression. As recognized by the main recent guidelines, preventing chronic psychological stress constitutes an important step to prevent the development of cardiovascular disease and the exacerbation of those (2). Controlled by the axis (hypothalamus-pituitary-adrenal glands), the stress induces an inappropriate secretion of adrenocorticotropic hormones in charge of the development of hypertension and insulin resistance. Through an imbalance of sympathetic and parasympathetic responses, peripheral resistances increase, and the secretion of epinephrine and norepinephrine maintains this mechanism. Moreover, the chronic stress induces an immune dysregulation, which promotes atherosclerosis, by increasing the production of pro-inflammatory biomarkers such as cytokines (51). Suppressing or limiting the progression of these stressors remains a major challenge at the workplace.

### Strength and limitation

Our umbrella review was based on systematic reviews, which used rigorous, high-quality methods leading to a selection of primary articles depending on their inclusion criteria. In counterpart, if the inclusion criteria were too restrictive, the authors may have overlooked some good-quality primary studies. Another limitation of this umbrella review is the possible misclassification of the type of shift work given the lack of detailed exposure characteristics, in particular in the oldest primary studies. To minimize this reporting bias, we provided a quality assessment with a reference tool (AMSTAR 2) and often carefully reviewed the original articles when definitions were unclear. In most systematic reviews undertaken, the healthy worker effect is difficult to assess, leading to a potential underestimation of the risk of night-shift work on cardiovascular factors.

The primary studies included in the selected systematic reviews gave an interesting wide overview of shift work in different occupations: nurses, factory workers (steel plant, semiconductors manufacturing, motor corporation, chemical industry, etc.), white-collar workers such as employees from public administration, but also workers from population-based cohorts gathering a wide range of occupations. Additionally, the results on the effects of night-shift work were obtained for jobs held in Europe, America and Asia, covering a large geographical area.

Given the huge number of studies and reviews undertaken on this topic, this umbrella review provides a summarized and updated overview of knowledge, useful for clinical practitioners and in occupational health. Our umbrella review constitutes a strong base to identify gaps in research and to promote future studies.

### Implication for future research

Some interesting results are provided from clinical trials, such as the rearranging of meal times at night (52), performing exercise sessions (53, 54), or the changes of shift rotation (55). Our umbrella review highlighted the need:

1) to detail the characteristics of night-shift work (working hours, direction of rotation, rotating schedules, etc.).
2) to better define the duration of exposure to night-shift work in working life (continuous or intermittent exposure) in order to assess a dose-response effect.
3) to evaluate the potential reversible health effect in former night-shift workers.
4) to deeply explore some outcomes such as sedentariness, working and leisure-time physical activity, smoking habits and occupational psychosocial stressors.
5) to develop interventional studies on potential mediators (i.e., diet, sleep) but also on the shift-work characteristics in order to counteract the adverse effects of night-shift work.

## Conclusion

This umbrella review reported evidence on the consequences of night-shift work on diabetes, being overweight/obesity and hypertension. In contrast, the links with lipid disorders, sedentariness, smoking habits, and occupational psychosocial stressors are worth being explored further. Monitoring these cardiovascular risk factors for night-shift workers could be implemented by practitioners. Given the widespread use of these working time patterns, it represents a major challenge for public health policies. In upcoming years, research must focus on evaluating the relevance of preventive countermeasures implemented in the workplace.

## Data availability statement

The original contributions presented in the study are included in the article/Supplementary material, further inquiries can be directed to the corresponding author/s.

## Author contributions

SB, EB, and YE: conception and design and methodology. SB, EB, YE, and JF: review of literature, substantial contributions to interpretation of data and have been involved in revising the manuscript it critically for important intellectual content, and writing and editing. All authors contributed to the article and approved the submitted version.

## Funding

This research has benefited from the joint assistance of the French National Health Insurance Fund for Employees (CNAMTS), the French Directorate General of Health (DGS), the Arc Foundation for Cancer Research, the French National Cancer Institute (INCA), the French National Institute for Prevention and Education in Health (INPES), the French National Institute of Health and Medical Research (INSERM), the French Inter-Departmental Agency for the Fight against Drugs and Addictive Behaviors (Mildeca), and the French Social Security Scheme for Liberal Professionals (RSI) as part of the Primary Prevention call for proposal issued by IReSP and INCA in 2013 and from CHU Toulouse. No role of sponsor in the writing, in analysis strategy, in the results, and discussion.

## Conflict of interest

The authors declare that the research was conducted in the absence of any commercial or financial relationships that could be construed as a potential conflict of interest.

## Publisher's note

All claims expressed in this article are solely those of the authors and do not necessarily represent those of their affiliated organizations, or those of the publisher, the editors and the reviewers. Any product that may be evaluated in this article, or claim that may be made by its manufacturer, is not guaranteed or endorsed by the publisher.
