# Supplementary material for: What do we know about the effect of night-shift work on cardiovascular risk factors? An umbrella review
Source: Front Public Health. 2022 Nov 23;10:1034195. doi: 10.3389/fpubh.2022.1034195 (PMC9727235; doi:10.3389/fpubh.2022.1034195)
Supplement: Supplementary file 1 [file Data_Sheet_1.PDF]

Supplemental table A. Detail of the Search strategies

Diabetes:<br><br>**#1 AND #2**<br>**Defined by: DOCUMENT TYPES:** (REVIEW) AND **TOPIC:** (humans) AND **LANGUAGES:** (ENGLISH OR FRENCH)<br>**Timespan:** All years<br>**Databases:** WOS, KJD, MEDLINE, RSCI, SCIELO<br>**#1:** TS = ("shift work" OR "shiftwork" OR "night work" OR "nightwork" OR "night shift work" OR "night-shift " OR "shiftworking" OR "rotating shift work" OR "12-hour shift" OR "evening shift" OR "morning shift" OR "long working hour ")<br>**#2:** TS = ("glycemia" OR "glucose" OR "diabetes" OR "diabetes mellitus" OR "NIDDM" OR "noninsulin dependent" OR "type II diabetes")
Lipid disorders:<br><br>**#1 AND #2**<br>**Refined by: DOCUMENT TYPES:** (REVIEW) AND **TOPIC:** (humans) AND **LANGUAGES:** (ENGLISH OR FRENCH)<br><br>**Timespan:** All years<br>**Databases:** WOS, KJD, MEDLINE, RSCI, SCIELO<br>**#1:** TS = ("shift work" OR "shiftwork" OR "night work" OR "nightwork" OR "night shift work" OR "night-shift " OR "shiftworking" OR "rotating shift work" OR "12-hour shift" OR "evening shift" OR "morning shift" OR "long working hour ")<br>**#2:** TS=("lipids" OR "cholesterol" OR "triglycerides" OR "apolipoprotein" OR "chylomicron" OR " very low-density lipoprotein" OR "low-density lipoprotein" OR " high-density lipoprotein")
Being overweight:<br><br>**#1 AND #2**<br>**Refined by: DOCUMENT TYPES:** (REVIEW) AND **TOPIC:** (humans) AND **LANGUAGES:** (ENGLISH OR FRENCH)<br>**Timespan:** All years<br>**Databases:** WOS, KJD, MEDLINE, RSCI, SCIELO<br>**#1:** TS = ("shift work" OR "shiftwork" OR "night work" OR "nightwork" OR "night shift work" OR "night-shift " OR "shiftworking" OR "rotating shift work" OR "12-hour shift" OR "evening shift" OR "morning shift" OR "long working hour ")<br>**#2:** TS = ("body mass index" OR "overweight" OR "obesity" OR "waist circumference")
Hypertension:<br><br>**#1 AND #2**<br>**Refined by: DOCUMENT TYPES:** (REVIEW) AND **TOPIC:** (humans) AND **LANGUAGES:** (ENGLISH OR FRENCH)<br>**Timespan:** All years<br>**Databases:** WOS, KJD, MEDLINE, RSCI, SCIELO

**#1:** TS = ("shift work" OR "shiftwork" OR "night work" OR "nightwork" OR "night shift work" OR "night-shift " OR "shiftworking" OR "rotating shift work" OR "12-hour shift" OR "evening shift" OR "morning shift" OR "long working hour ")<br>**#2:** TS = ("blood pressure" OR "hypertension" OR "high blood pressure" OR "systolic pressure" OR "diastolic pressure")
Smoking habits<br><br>**#1 AND #2**<br>**Refined by: DOCUMENT TYPES:** (REVIEW) AND **TOPIC:** (humans) AND **LANGUAGES:** (ENGLISH OR FRENCH)<br>**Timespan:** All years<br>**Databases:** WOS, KJD, MEDLINE, RSCI, SCIELO<br>**#1:** TS = ("shift work" OR "shiftwork" OR "night work" OR "nightwork" OR "night shift work" OR "night-shift " OR "shiftworking" OR "rotating shift work" OR "12-hour shift" OR "evening shift" OR "morning shift" OR "long working hour ")<br>**#2:** TS= (tobacco OR smoking)
Sedentariness<br><br>**#1 AND #2**<br>**Refined by: DOCUMENT TYPES:** (REVIEW) AND **TOPIC:** (humans) AND **LANGUAGES:** (ENGLISH OR FRENCH)<br>**Timespan:** All years<br>**Databases:** WOS, KJD, MEDLINE, RSCI, SCIELO<br>**#1:** TS = ("shift work" OR "shiftwork" OR "night work" OR "nightwork" OR "night shift work" OR "night-shift " OR "shiftworking" OR "rotating shift work" OR "12-hour shift" OR "evening shift" OR "morning shift" OR "long working hour ")<br>**#2:** TS= (sedentary OR sedentariness OR sedentarity OR physical activity)
Occupational psychosocial stressors<br><br>**#1 AND #2**<br>**Refined by: DOCUMENT TYPES:** (REVIEW) AND **TOPIC:** (humans) AND **LANGUAGES:** (ENGLISH OR FRENCH)<br>**Timespan:** All years<br>**Databases:** WOS, KJD, MEDLINE, RSCI, SCIELO<br>**#1**: TS = ("shift work" OR "shiftwork" OR "night work" OR "nightwork" OR "night shift work" OR "night-shift " OR "shiftworking" OR "rotating shift work" OR "12-hour shift" OR "evening shift" OR "morning shift" OR "long working hour ")<br>***#2: TS= (psychosocial risk OR psychosocial factors OR stress)***

Supplemental table B. List of excluded articles and reasons for exclusion after reading the full text

DIABETES	IDENTIFICATION	SCREENING		ELIGIBILITY	INCLUDED	REASON FOR EXCLUSION
	SEARCH / REFERENCE LIST	TITLE	ABSTRACT	FULL TEXT	FULL TEST	
Adams, S. T., et al. (2013). "Roux-en-Y gastric bypass for morbid obesity: what are the preoperative predictors of weight loss?" Postgraduate Medical Journal 89(1053): 411-416.	1	0	0	0	0	
Afroz-Hossain, A., et al. (2019). "Sleep and Environmental Factors Affecting Glycemic Control in People with Type 2 Diabetes Mellitus." Current Diabetes Reports 19(7).	1	1	0	0	0	
Allaf, M., et al. (2021). "Intermittent fasting for the prevention of cardiovascular disease." Cochrane Database of Systematic Reviews(1).	1	0	0	0	0	
Allison, K. C. and N. Goel (2018). "Timing of eating in adults across the weight spectrum: Metabolic factors and potential circadian mechanisms." Physiology & Behavior 192: 158-166.	1	0	0	0	0	
Amaral, F. G., et al. (2014). "Environmental Control of Biological Rhythms: Effects on Development, Fertility and Metabolism." Journal of Neuroendocrinology 26(9): 603-612.	1	0	0	0	0	
Amiri, S. and S. Behnezhad (2020). "Sleep disturbances and risk of sick leave: systematic review and meta-analysis." Sleep and Biological Rhythms 18(4): 283-295.	1	0	0	0	0	
Andriessen, C., et al. (2021). "The importance of 24-h metabolism in obesity-related metabolic disorders: opportunities for timed interventions." International Journal of Obesity 45(3): 479-490.	1	0	0	0	0	
Angeles Bonmati-Carrion, M., et al. (2014). "Protecting the Melatonin Rhythm through Circadian Healthy Light Exposure." International Journal of Molecular Sciences 15(12): 23448-23500.	1	0	0	0	0	
Angelousi, A., et al. (2018). "Clock genes alterations and endocrine disorders." European Journal of Clinical Investigation 48(6).	1	0	0	0	0	
Antunes, L. C., et al. (2010). "Obesity and shift work: chronobiological aspects." Nutrition Research Reviews 23(1): 155-168.	1	1	1	1	0	NO EVALUATION OF THE ASSOCIATION BETWEEN SW AND OUTCOME
Ashby, T. and M. Louis (2019). "Circadian Misalignment and Cardiovascular Risk." Cardiovascular Innovations and Applications 3(4): 435-440.	1	1	1	1	0	NARRATIVE

Atkinson, G., et al. (2008). "Exercise, energy balance and the shift worker." Sports Medicine 38(8): 671-685.	1	1	0	0	0	
Azmi, N., et al. (2020). "Consequences of Circadian Disruption in Shift Workers on Chrononutrition and their Psychosocial Well-Being." International Journal of Environmental Research and Public Health 17(6).	1	0	0	0	0	
Bae, S.-A., et al. (2019). "At the Interface of Lifestyle, Behavior, and Circadian Rhythms: Metabolic Implications." Frontiers in Nutrition 6.	1	0	0	0	0	
Bailey, S. M., et al. (2014). "Circadian regulation of metabolism." Journal of Endocrinology 222(2): R75-R96.	1	0	0	0	0	
Bannai, A. and A. Tamakoshi (2014). "The association between long working hours and health: A systematic review of epidemiological evidence." Scandinavian Journal of Work Environment & Health 40(1): 5-18.	1	1	0	0	0	
Baron, K. G. and K. J. Reid (2014). "Circadian misalignment and health." International Review of Psychiatry 26(2): 139-154.	1	0	0	0	0	
Bass, J. and J. S. Takahashi (2010). "Circadian Integration of Metabolism and Energetics." Science 330(6009): 1349-1354.	1	0	0	0	0	
Boivin, D. B. and F. O. James (2005). "Light treatment and circadian adaptation to shift work." Industrial Health 43(1): 34-48.	1	0	0	0	0	
Boivin, D. B., et al. (2007). "Working on atypical schedules." Sleep Medicine 8(6): 578-589.	1	1	0	0	0	
Bonham, M. P., et al. (2016). "Energy intake of shift workers compared to fixed day workers: A systematic review and meta-analysis." Chronobiology International 33(8): 1086-1100.	1	1	0	0	0	
Broussard, J. and M. J. Brady (2010). "The impact of sleep disturbances on adipocyte function and lipid metabolism." Best Practice & Research Clinical Endocrinology & Metabolism 24(5): 763-773.	1	0	0	0	0	
Broussard, J. L. and S. Devkota (2016). "The changing microbial landscape of Western society: Diet, dwellings and discordance." Molecular Metabolism 5(9): 737-742.	1	0	0	0	0	
Brubakk, A. O., et al. (2014). "Saturation Diving; Physiology and Pathophysiology." Comprehensive Physiology 4(3): 1229-1272.	1	0	0	0	0	
Buijs, F. N., et al. (2016). "The Circadian System: A Regulatory Feedback Network of Periphery and Brain." Physiology 31(3): 170-181.	1	0	0	0	0	

Buijs, R. M., et al. (2013). "The circadian system and the balance of the autonomic nervous system." Handbook of clinical neurology 117: 173-191.	1	0	0	0	0	
Buijs, R. M., et al. (2019). "The suprachiasmatic nucleus; a responsive clock regulating homeostasis by daily changing the setpoints of physiological parameters." Autonomic Neuroscience-Basic & Clinical 218: 43-50.	1	0	0	0	0	
Buijs, R., et al. (2013). Peripheral Circadian Oscillators: Time and Food. Chronobiology: Biological Timing in Health and Diesease. M. U. Gillette. 119: 83-103.	1	0	0	0	0	
Burman, D. (2017). "Sleep Disorders: Circadian Rhythm Sleep-Wake Disorders." FP essentials 460: 33-36.	1	0	0	0	0	
Cagampang, F. R. and K. D. Bruce (2012). "The role of the circadian clock system in nutrition and metabolism." British Journal of Nutrition 108(3): 381-392.	1	0	0	0	0	
Cai, C., et al. (2019). "The impact of occupational shift work and working hours during pregnancy on health outcomes: a systematic review and meta-analysis." American Journal of Obstetrics and Gynecology 221(6): 563-576.	1	1	0	0	0	
Cai, C., et al. (2020). "The impact of occupational activities during pregnancy on pregnancy outcomes: a systematic review and metaanalysis." American Journal of Obstetrics and Gynecology 222(3): 224-238.	1	0	0	0	0	
Campagna, M., et al. (2016). "Metabolomic patterns associated to QTc interval in shiftworkers: an explorative analysis." Biomarkers 21(7): 607-613.	1	0	0	0	0	
Canuto, R., et al. (2013). "Metabolic syndrome and shift work: A systematic review." Sleep Medicine Reviews 17(6): 425-431.	1	1	1	1	0	NO EVALUATION OF THE ASSOCIATION BETWEEN SW AND OUTCOME
Cappuccio, F. P. and M. A. Miller (2017). "Sleep and Cardio-Metabolic Disease." Current Cardiology Reports 19(11).	1	0	0	0	0	
Carroll, R. G., et al. (2019). "Immunometabolism around the Clock." Trends in Molecular Medicine 25(7): 612-625.	1	0	0	0	0	
Cayanan, E. A., et al. (2019). "Is 24-hour energy intake greater during night shift compared to non-night shift patterns? A systematic review." Chronobiology International 36(12): 1599-1612.	1	0	0	0	0	
Chaix, A., et al. (2019). Time-Restricted Eating to Prevent and Manage Chronic Metabolic Diseases. Annual Review of Nutrition, Vol 39, 2019. P. J. Stover and R. Balling. 39: 291-315.	1	0	0	0	0	

Challet, E. (2013). Circadian Clocks, Food Intake, and Metabolism. Chronobiology: Biological Timing in Health and Diesease. M. U. Gillette. 119: 105-135.	1	0	0	0	0	
Challet, E. (2015). "Keeping circadian time with hormones." Diabetes Obesity & Metabolism 17: 76-83.	1	0	0	0	0	
Chao, H.-W., et al. (2019). "Homeostasis of Glucose and Lipid in Non-Alcoholic Fatty Liver Disease." International Journal of Molecular Sciences 20(2).	1	0	0	0	0	
Charlot, A., et al. (2021). "Beneficial Effects of Early Time-Restricted Feeding on Metabolic Diseases: Importance of Aligning Food Habits with the Circadian Clock." Nutrients 13(5).	1	0	0	0	0	
Cipolla-Neto, J., et al. (2014). "Melatonin, energy metabolism, and obesity: a review." Journal of Pineal Research 56(4): 371-381.	1	0	0	0	0	
Covassin, N., et al. (2016). "Keeping Up With the Clock: Circadian Disruption and Obesity Risk." Hypertension 68(5): 1081-1090.	1	0	0	0	0	
de Goede, P., et al. (2018). "Circadian rhythms in mitochondrial respiration." Journal of Molecular Endocrinology 60(3): R115-R130.	1	0	0	0	0	
Demou, E., et al. (2018). "Group-based healthy lifestyle workplace interventions for shift workers: a systematic review." Scandinavian Journal of Work Environment & Health 44(6): 568-584.	1	1	0	0	0	
Deng, N., et al. (2018). "The Relationship Between Shift Work and Men's Health." Sexual Medicine Reviews 6(3): 446-456.	1	0	0	0	0	
Depner, C. M., et al. (2014). "Metabolic Consequences of Sleep and Circadian Disorders." Current Diabetes Reports 14(7).	1	0	0	0	0	
Dibner, C. and U. Schibler (2015). "Circadian timing of metabolism in animal models and humans." Journal of Internal Medicine 277(5): 513-527.	1	0	0	0	0	
Diene, E., et al. (2012). "Cardiovascular diseases and psychosocial factors at work." Archives of Cardiovascular Diseases 105(1): 33-39.	1	0	0	0	0	
Ding, C., et al. (2018). "Sleep and Obesity." Journal of Obesity & Metabolic Syndrome 27(1): 4-24.	1	0	0	0	0	
Ding, L. and X.-H. Xiao (2020). "Gut microbiota: closely tied to the regulation of circadian clock in the development of type 2 diabetes mellitus." Chinese Medical Journal 133(7): 817-825.	1	0	0	0	0	

Dollet, L. and J. R. Zierath (2019). "Interplay between diet, exercise and the molecular circadian clock in orchestrating metabolic adaptations of adipose tissue." Journal of Physiology-London 597(6): 1439-1450.	1	0	0	0	0	
Duez, H. and B. Staels (2009). "Rev-erb-alpha: an integrator of circadian rhythms and metabolism." Journal of Applied Physiology 107(6): 1972-1980.	1	0	0	0	0	
Duez, H. and B. Staels (2010). "Nuclear Receptors Linking Circadian Rhythms and Cardiometabolic Control." Arteriosclerosis Thrombosis and Vascular Biology 30(8): 1529-1534.	1	0	0	0	0	
Eckel-Mahan, K. and P. Sassone-Corsi (2013). "METABOLISM AND THE CIRCADIAN CLOCK CONVERGE." Physiological Reviews 93(1): 107-135.	1	0	0	0	0	
Engin, A. (2017). "Circadian Rhythms in Diet-Induced Obesity." Advances in experimental medicine and biology 960: 19-52.	1	0	0	0	0	
Fatima, N. and Sobia (2020). "Metabolic implications of circadian disruption." Pflugers Archiv-European Journal of Physiology 472(5): 513-526.	1	0	0	0	0	
Ferrell, J. M. and J. Y. L. Chiang (2015). "Circadian rhythms in liver metabolism and disease." Acta Pharmaceutica Sinica B 5(2): 113-122.	1	0	0	0	0	
Ferri, G. M., et al. (2019). "Healthy Diet and Reduction of Chronic Disease Risks of Night Shift Workers." Current Medicinal Chemistry 26(19): 3521-3541.	1	1	0	0	0	
Figueiro, M. G. and R. D. White (2013). "Health consequences of shift work and implications for structural design." Journal of Perinatology 33: S17-S23.	1	1	1	1	0	NO EVALUATION OF THE ASSOCIATION BETWEEN SW AND OUTCOME
Finger, A. M., et al. (2020). "Coupled network of the circadian clocks: a driving force of rhythmic physiology." Febs Letters 594(17): 2734-2769.	1	0	0	0	0	
Forrestel, A. C., et al. (2017). "Chronomedicine and type 2 diabetes: shining some light on melatonin." Diabetologia 60(5): 808-822.	1	0	0	0	0	
Forsyth, C. B., et al. (2015). "Circadian rhythms, alcohol and gut interactions." Alcohol 49(4): 389-398.	1	0	0	0	0	
Frazier, K., et al. (2020). "Mediators of Host-Microbe Circadian Rhythms in Immunity and Metabolism." Biology-Basel 9(12).	1	0	0	0	0	

Gamaldo, C. E., et al. (2014). "Tick-tock-tick-tock: the impact of circadian rhythm disorders on cardiovascular health and wellness." Journal of the American Society of Hypertension 8(12): 921-929.	1	1	0	0	0	
Gangwisch, J. E. (2009). "Epidemiological evidence for the links between sleep, circadian rhythms and metabolism." Obesity Reviews 10: 37-45.	1	0	0	0	0	
Garaulet, M. and J. A. Madrid (2009). "Chronobiology, genetics and metabolic syndrome." Current Opinion in Lipidology 20(2): 127-134.	1	0	0	0	0	
Garaulet, M., et al. (2020). "Melatonin Effects on Glucose Metabolism: Time To Unlock the Controversy." Trends in Endocrinology and Metabolism 31(3): 192-204.	1	0	0	0	0	
Gende, M. R. A. (2021). "Cognitive impairment of resident doctors due to sleep deprivation in 24-hour guards." Revista San Gregorio(45): 175-192.	1	0	0	0	0	
Gerhart-Hines, Z. and M. A. Lazar (2015). "Circadian Metabolism in the Light of Evolution." Endocrine Reviews 36(3): 289-304.	1	0	0	0	0	
Gibson, E. M., et al. (2009). "Aging in the circadian system: Considerations for health, disease prevention and longevity." Experimental Gerontology 44(1-2): 51-56.	1	0	0	0	0	
Gillie, O. (2010). "Sunlight robbery: A critique of public health policy on vitamin D in the UK." Molecular Nutrition & Food Research 54(8): 1148-1163.	1	0	0	0	0	
Gohari, A., et al. "Shift working and cardiovascular health." Chronobiology International.	1	1	0	0	0	
Gonnissen, H. K. J., et al. (2013). "Chronobiology, endocrinology, and energy- and food-reward homeostasis." Obesity Reviews 14(5): 405-416.	1	0	0	0	0	
Gonzalez-Gonzalez, A., et al. (2018). "Melatonin: A Molecule for Reducing Breast Cancer Risk." Molecules 23(2).	1	0	0	0	0	
Guerrero-Vargas, N. N., et al. (2018). "Shift-work: is time of eating determining metabolic health? Evidence from animal models." Proceedings of the Nutrition Society 77(3): 199-215.	1	0	0	0	0	
Hardeland, R. (2014). "Melatonin, Noncoding RNAs, Messenger RNA Stability and Epigenetics-Evidence, Hints, Gaps and Perspectives." International Journal of Molecular Sciences 15(10): 18221-18252.	1	0	0	0	0	
Harfmann, B. D., et al. (2015). "Circadian Rhythms, the Molecular Clock, and Skeletal Muscle." Journal of Biological Rhythms 30(2): 84-94.	1	0	0	0	0	

Harma, M. (2006). "Workhours in relation to work stress, recovery and health." Scandinavian Journal of Work Environment & Health 32(6): 502-514.	1	1	1	1	0	NO EVALUATION OF THE ASSOCIATION BETWEEN SW AND OUTCOME
Hasler, B. P., et al. (2015). "Sleep and circadian contributions to adolescent alcohol use disorder." Alcohol 49(4): 377-387.	1	0	0	0	0	
Hatori, M. and S. Panda (2015). Response of Peripheral Rhythms to the Timing of Food Intake. Circadian Rhythms and Biological Clocks, Pt B. A. Sehgal. 552: 145-161.	1	0	0	0	0	
Haus, E. L. and M. H. Smolensky (2013). "Shift work and cancer risk: Potential mechanistic roles of circadian disruption, light at night, and sleep deprivation." Sleep Medicine Reviews 17(4): 273-284.	1	0	0	0	0	
Henry, C. J., et al. (2020). "Chrononutrition in the management of diabetes." Nutrition & Diabetes 10(1).	1	0	0	0	0	
Heyde, I., et al. (2018). "Mutual influence of sleep and circadian clocks on physiology and cognition." Free Radical Biology and Medicine 119: 8-16.	1	0	0	0	0	
Hruby, A., et al. (2016). "Determinants and Consequences of Obesity." American Journal of Public Health 106(9): 1656-1662.	1	0	0	0	0	
Iavicoli, I., et al. (2019). "Diabetes and work: The need of a close collaboration between diabetologist and occupational physician." Nutrition Metabolism and Cardiovascular Diseases 29(3): 220-227.	1	1	1	1	0	NO EVALUATION OF THE ASSOCIATION BETWEEN SW AND OUTCOME
Ikegami, K., et al. (2019). "Interconnection between circadian clocks and thyroid function." Nature Reviews Endocrinology 15(10): 590-600.	1	0	0	0	0	
Ioja, S., et al. (2012). "Relationship Between Sleep Disorders and the Risk for Developing Type 2 Diabetes Mellitus." Postgraduate Medicine 124(4): 119-129.	1	0	0	0	0	
Ivanov, D. O., et al. (2020). "The Role of Prenatal Melatonin in the Regulation of Childhood Obesity." Biology 9(4).	1	0	0	0	0	
Jha, P. K., et al. (2015). "Circadian rhythms in glucose and lipid metabolism in nocturnal and diurnal mammals." Molecular and Cellular Endocrinology 418: 74-88.	1	0	0	0	0	
Jiang, P. and F. W. Turek (2018). "The endogenous circadian clock programs animals to eat at certain times of the 24-hour day: What if we ignore the clock?" Physiology & Behavior 193: 211-217.	1	0	0	0	0	
Johnston, J. D. (2014). "Physiological responses to food intake throughout the day." Nutrition Research Reviews 27(1): 107-118.	1	0	0	0	0	

Johnston, J. D., et al. (2016). "Circadian Rhythms, Metabolism, and Chrononutrition in Rodents and Humans." Advances in Nutrition 7(2): 399-406.	1	0	0	0	0	
Karthikeyan, R., et al. (2014). "Should we listen to our clock to prevent type 2 diabetes mellitus?" Diabetes Research and Clinical Practice 106(2): 182-190.	1	1	0	0	0	
Ke, D.-S. (2012). "Overwork, stroke, and karoshi-death from overwork." Acta neurologica Taiwanica 21(2): 54-59.	1	0	0	0	0	
Kerkhof, G. A. and H. P. A. Van Dongen (2010). Circadian rhythms and cognition. Human Sleep and Cognition, Part I: Basic Research. G. A. Kerkhof and H. P. A. van Dongen. 185: 131-153.	1	0	0	0	0	
Kervezee, L., et al. (2018). "Impact of Shift Work on the Circadian Timing System and Health in Women." Sleep Medicine Clinics 13(3): 295-306.	1	1	0	0	0	
Kervezee, L., et al. (2020). "Metabolic and cardiovascular consequences of shift work: The role of circadian disruption and sleep disturbances." European Journal of Neuroscience 51(1): 396-412.	1	1	1	1	0	NARRATIVE
Kessler, K. and O. Pivovarova-Ramich (2019). "Meal Timing, Aging, and Metabolic Health." International Journal of Molecular Sciences 20(8).	1	0	0	0	0	
Khan, S., et al. (2018). "Health risks associated with genetic alterations in internal clock system by external factors." International Journal of Biological Sciences 14(7): 791-798.	1	0	0	0	0	
Knutsson, A. (2003). "Health disorders of shift workers." Occupational Medicine-Oxford 53(2): 103-108.	1	1	1	1	0	NARRATIVE
Kolbe, I. and H. Oster (2019). "Chronodisruption, Metabolic Homeostasis, and the Regulation of Inflammation in Adipose Tissues." Yale Journal of Biology and Medicine 92(2): 317-325.	1	0	0	0	0	
Koren, D., et al. (2015). "Metabolic and Glycemic Sequelae of Sleep Disturbances in Children and Adults." Current Diabetes Reports 15(1).	1	0	0	0	0	
Kozlowska, L., et al. (2019). "HEALTH RISK IN TRANSPORT WORKERS PART II. DIETARY COMPOUNDS AS MODULATORS OF OCCUPATIONAL EXPOSURE TO CHEMICALS." International Journal of Occupational Medicine and Environmental Health 32(4): 441-464.	1	0	0	0	0	
Lee, J., et al. (2015). "Circadian control of -cell function and stress responses." Diabetes Obesity & Metabolism 17: 123-133.	1	0	0	0	0	

Lee, J., et al. (2018). "Untimely oxidative stress in beta-cells leads to diabetes - Role of circadian clock in beta-cell function." Free Radical Biology and Medicine 119: 69-74.	1	0	0	0	0	
Leger, D., et al. (2018). "Shift-workers and night-workers' health consequences: State of art and recommendations." Presse Medicale 47(11-12): 991-999.	1	1	1	1	0	NARRATIVE
Leung, G. K. W., et al. (2020). "Time of day difference in postprandial glucose and insulin responses: Systematic review and meta-analysis of acute postprandial studies." Chronobiology International 37(3): 311-326.	1	0	0	0	0	
Liou, T. G. (2019). "The Clinical Biology of Cystic Fibrosis Transmembrane Regulator Protein Its Role and Function in Extrapulmonary Disease." Chest 155(3): 605-616.	1	0	0	0	0	
Longo, V. D. and S. Panda (2016). "Fasting, Circadian Rhythms, and Time-Restricted Feeding in Healthy Lifespan." Cell Metabolism 23(6): 1048-1059.	1	0	0	0	0	
Maiese, K. (2017). "Moving to the Rhythm with Clock (Circadian) Genes, Autophagy, mTOR, and SIRT1 in Degenerative Disease and Cancer." Current Neurovascular Research 14(3): 299-304.	1	0	0	0	0	
Martchenko, A., et al. (2020). "Circadian Rhythms and the Gastrointestinal Tract: Relationship to Metabolism and Gut Hormones." Endocrinology 161(12).	1	0	0	0	0	
Martino, T. A. and M. E. Young (2015). "Influence of the Cardiomyocyte Circadian Clock on Cardiac Physiology and Pathophysiology." Journal of Biological Rhythms 30(3): 183-205.	1	0	0	0	0	
Mason, I. C., et al. (2020). "Impact of circadian disruption on glucose metabolism: implications for type 2 diabetes." Diabetologia 63(3): 462-472.	1	1	0	0	0	
Maury, E. (2019). "Off the Clock: From Circadian Disruption to Metabolic Disease." International Journal of Molecular Sciences 20(7).	1	0	0	0	0	
Mayeuf-Louchart, A., et al. (2017). "Circadian control of metabolism and pathological consequences of clock perturbations." Biochimie 143: 42-50.	1	0	0	0	0	
McHill, A. W. and K. P. Wright, Jr. (2017). "Role of sleep and circadian disruption on energy expenditure and in metabolic predisposition to human obesity and metabolic disease." Obesity Reviews 18: 15-24.	1	0	0	0	0	
Meiliana, A., et al. (2015). "Chronodisruption and Obesity." Indonesian Biomedical Journal 7(3): 117-128.	1	0	0	0	0	

Moran-Ramos, S.-A., et al. (2016). "When to eat? The influence of circadian rhythms on metabolic health: are animal studies providing the evidence?" Nutrition Research Reviews 29(2): 180-193.	1	0	0	0	0	
Morgan, D. and S. C. Tsai (2015). "Sleep and the Endocrine System." Critical Care Clinics 31(3): 403-+.	1	0	0	0	0	
Morgan, L., et al. (2003). "Circadian aspects of postprandial metabolism." Chronobiology International 20(5): 795-808.	1	0	0	0	0	
Morris, C. J., et al. (2012). "Circadian system, sleep and endocrinology." Molecular and Cellular Endocrinology 349(1): 91-104.	1	0	0	0	0	
Morris, C. J., et al. (2012). The impact of the circadian timing system on cardiovascular and metabolic function. Neurobiology of Circadian Timing. A. Kalsbeek, M. Merrow, T. Roenneberg and R. G. Foster. 199: 337-358.	1	1	0	0	0	
Mosendane, T., et al. (2008). "Shift work and its effects on the cardiovascular system." Cardiovascular Journal of Africa 19(4): 210-215.	1	1	1	1	0	MECHANISTIC
Mulder, H., et al. (2009). "Melatonin receptors in pancreatic islets: good morning to a novel type 2 diabetes gene." Diabetologia 52(7): 1240-1249.	1	0	0	0	0	
Nader, N., et al. (2010). "Interactions of the circadian CLOCK system and the HPA axis." Trends in Endocrinology and Metabolism 21(5): 277-286.	1	0	0	0	0	
Narita, K. and E. Amiya "Social and environmental risks as contributors to the clinical course of heart failure." Heart Failure Reviews.	1	0	0	0	0	
Nedeltcheva, A. V. and F. A. J. L. Scheer (2014). "Metabolic effects of sleep disruption, links to obesity and diabetes." Current Opinion in Endocrinology Diabetes and Obesity 21(4): 293-298.	1	1	0	0	0	
Nicholson, P. J. and D. A. P. D'Auria (1999). "Shift work, health, the working time regulations and health assessments." Occupational Medicine-Oxford 49(3): 127-137.	1	1	0	0	0	
Nohara, K., et al. (2015). "Manipulating the circadian and sleep cycles to protect against metabolic disease." Frontiers in Endocrinology 6.	1	0	0	0	0	
Ogilvie, R. P. and S. R. Patel (2018). "The Epidemiology of Sleep and Diabetes." Current Diabetes Reports 18(10).	1	1	1	1	0	NARRATIVE

Olaoye, O. A., et al. (2019). "Circadian Clock Genes in Diabetic Kidney Disease (DKD)." Current Diabetes Reports 19(7).	1	0	0	0	0	
Onaolapo, A. Y., et al. (2019). "Cerebrovascular Disease in the Young Adult: Examining Melatonin's Possible Multiple Roles." Journal of Experimental Neuroscience 13.	1	0	0	0	0	
Oosterman, J. E., et al. (2020). "The Circadian Clock, Shift Work, and Tissue-Specific Insulin Resistance." Endocrinology 161(12).	1	0	0	0	0	
Opperhuizen, A.-L., et al. (2015). "Rodent models to study the metabolic effects of shiftwork in humans." Frontiers in Pharmacology 6.	1	0	0	0	0	
Otamas, A., et al. (2020). "Diabetes and atherothrombosis: The circadian rhythm and role of melatonin in vascular protection." Diabetes & Vascular Disease Research 17(3).	1	0	0	0	0	
Pagano, E. S., et al. (2017). "White Adipose Tissue and Circadian Rhythm Dysfunctions in Obesity: Pathogenesis and Available Therapies." Neuroendocrinology 104(4): 347-363.	1	0	0	0	0	
Paschos, G. K. (2021). "Diurnal rhythms and obesity." Current Opinion in Clinical Nutrition and Metabolic Care 24(4): 333-338.	1	0	0	0	0	
Phoi, Y. Y. and J. B. Keogh (2019). "Dietary Interventions for Night Shift Workers: A Literature Review." Nutrients 11(10).	1	0	0	0	0	
Plano, S. A., et al. (2017). "Circadian and Metabolic Effects of Light: Implications in Weight Homeostasis and Health." Frontiers in Neurology 8.	1	0	0	0	0	
Porkka-Heiskanen, T., et al. (2013). "Sleep, its regulation and possible mechanisms of sleep disturbances." Acta Physiologica 208(4): 311-328.	1	0	0	0	0	
Porter, J., et al. (2017). "Is physiological glucocorticoid replacement important in children?" Archives of Disease in Childhood 102(2): 199-205.	1	0	0	0	0	
Potter, G. D. M. and T. R. Wood (2020). "The Future of Shift Work: Circadian Biology Meets Personalised Medicine and Behavioural Science." Frontiers in Nutrition 7.	1	0	0	0	0	
Potter, G. D. M., et al. (2016). "Circadian Rhythm and Sleep Disruption: Causes, Metabolic Consequences, and Countermeasures." Endocrine Reviews 37(6): 584-608.	1	1	0	0	0	
Pourcet, B. and H. Duez (2020). "Circadian Control of Inflammasome Pathways: Implications for Circadian Medicine." Frontiers in Immunology 11.	1	0	0	0	0	

Prasai, M. J., et al. (2008). "Molecular clocks, type 2 diabetes and cardiovascular disease." Diabetes & Vascular Disease Research 5(2): 89-95.	1	0	0	0	0	
Puttonen, S., et al. (2010). "Shift work and cardiovascular disease - pathways from circadian stress to morbidity." Scandinavian Journal of Work Environment & Health 36(2): 96-108.	1	1	1	1	0	NARRATIVE
Qian, J. and F. A. J. L. Scheer (2016). "Circadian System and Glucose Metabolism: Implications for Physiology and Disease." Trends in Endocrinology and Metabolism 27(5): 282-293.	1	0	0	0	0	
Rakshit, K., et al. (2014). "Does Disruption of Circadian Rhythms Contribute to Beta-Cell Failure in Type 2 Diabetes?" Current Diabetes Reports 14(4).	1	0	0	0	0	
Reutrakul, S. and K. L. Knutson (2015). "Consequences of Circadian Disruption on Cardiometabolic Health." Sleep Medicine Clinics 10(4): 455-468.	1	0	0	0	0	
Reynolds, A. C., et al. (2017). "The shift work and health research agenda: Considering changes in gut microbiota as a pathway linking shift work, sleep loss and circadian misalignment, and metabolic disease." Sleep Medicine Reviews 34: 3-9.	1	0	0	0	0	
Rosa, D., et al. (2020). "The relationship between urolithiasis, metabolic syndrome and nurse shift work. A literature review." International Journal of Urological Nursing 14(2): 57-66.	1	0	0	0	0	
Ruddick-Collins, L. C., et al. (2020). "Mealtime: A circadian disruptor and determinant of energy balance?" Journal of Neuroendocrinology 32(7).	1	0	0	0	0	
Rueger, M. and F. A. J. L. Scheer (2009). "Effects of circadian disruption on the cardiometabolic system." Reviews in Endocrine & Metabolic Disorders 10(4): 245-260.	1	0	0	0	0	
Rynders, C. A., et al. (2019). "Effectiveness of Intermittent Fasting and Time-Restricted Feeding Compared to Continuous Energy Restriction for Weight Loss." Nutrients 11(10).	1	0	0	0	0	
Sahar, S. and P. Sassone-Corsi (2012). "Regulation of metabolism: the circadian clock dictates the time." Trends in Endocrinology and Metabolism 23(1): 1-8.	1	0	0	0	0	
Sakuraya, A., et al. (2017). "Work-related psychosocial factors and onset of metabolic syndrome among workers: a systematic review and meta-analysis protocol." Bmj Open 7(6).	1	1	1	1	0	STUDY PROTOCOL
Samanta, S. "Physiological and pharmacological perspectives of melatonin." Archives of Physiology and Biochemistry.	1	0	0	0	0	

Sanchez-Barcelo, E. J., et al. (2010). "Clinical Uses of Melatonin: Evaluation of Human Trials." Current Medicinal Chemistry 17(19): 2070-2095.	1	0	0	0	0	
Santana-Herrera, J., et al. (2014). "Turnos de trabajo: ¿un factor de riesgo cardiovascular" Medicina y Seguridad del Trabajo 60(234): 179-197.	1	0	0	0	0	
Schilperoort, M., et al. (2020). "Time for Novel Strategies to Mitigate Cardiometabolic Risk in Shift Workers." Trends in Endocrinology and Metabolism 31(12): 952-964.	1	1	1	1	0	NARRATIVE
Schipper, S. B. J., et al. "Sleep disorders in people with type 2 diabetes and associated health outcomes: a review of the literature." Diabetologia.	1	0	0	0	0	
Shetty, A., et al. (2018). "Role of the Circadian Clock in the Metabolic Syndrome and Nonalcoholic Fatty Liver Disease." Digestive Diseases and Sciences 63(12): 3187-3206.	1	0	0	0	0	
Skaer, T. L. and D. A. Sclar (2010). "Economic Implications of Sleep Disorders." Pharmacoeconomics 28(11): 1015-1023.	1	0	0	0	0	
Slanovic-Kuzmanovic, Z., et al. (2013). "ENDOCRINE, LIFESTYLE, AND GENETIC FACTORS IN THE DEVELOPMENT OF METABOLIC SYNDROME." Arhiv Za Higijenu Rada I Toksikologiju-Archives of Industrial Hygiene and Toxicology 64(4): 581-591.	1	0	0	0	0	
Smyth, A., et al. (2020). "Systematic review of clinical practice guidelines to identify recommendations for sleep in type 2 diabetes mellitus management." Diabetes Research and Clinical Practice 170.	1	0	0	0	0	
Sookoian, S. and C. J. Pirola (2013). "Epigenetics of Insulin Resistance: An Emerging Field in Translational Medicine." Current Diabetes Reports 13(2): 229-237.	1	0	0	0	0	
Stenvers, D. J., et al. (2019). "Circadian clocks and insulin resistance." Nature Reviews Endocrinology 15(2): 75-89.	1	0	0	0	0	
Stevens, R. G. and Y. Zhu (2015). "Electric light, particularly at night, disrupts human circadian rhythmicity: is that a problem?" Philosophical Transactions of the Royal Society B-Biological Sciences 370(1667).	1	0	0	0	0	
Straat, M. E., et al. (2021). "Circadian control of brown adipose tissue." Biochimica Et Biophysica Acta-Molecular and Cell Biology of Lipids 1866(8).	1	0	0	0	0	
Strohmaier, S., et al. (2018). "A Review of Data of Findings on Night Shift Work and the Development of DM and CVD Events: a Synthesis	1	1	0	0	0	

of the Proposed Molecular Mechanisms." Current Diabetes Reports 18(12).						
Sulli, G., et al. (2018). "Training the Circadian Clock, Clocking the Drugs, and Drugging the Clock to Prevent, Manage, and Treat Chronic Diseases." Trends in Pharmacological Sciences 39(9): 812-827.	1	0	0	0	0	
Szosland, D. (2010). "SHIFT WORK AND METABOLIC SYNDROME, DIABETES MELLITUS AND ISCHAEMIC HEART DISEASE." International Journal of Occupational Medicine and Environmental Health 23(3): 287-291.	1	1	1	1	0	NARRATIVE
Tamashiro, K. L., et al. (2011). "Chronic stress, metabolism, and metabolic syndrome." Stress-the International Journal on the Biology of Stress 14(5): 468-474.	1	0	0	0	0	
Thorpy, M. J., et al. (2007). "Patient-management strategies." American Journal of Managed Care 13(6): S140-S147.	1	0	0	0	0	
Tirabassi, G., et al. (2014). "Harmful effects of functional hypercortisolism: a working hypothesis." Endocrine 46(3): 370-386.	1	0	0	0	0	
Touitou, Y., et al. (2017). "Association between light at night, melatonin secretion, sleep deprivation, and the internal clock: Health impacts and mechanisms of circadian disruption." Life Sciences 173: 94-106.	1	0	0	0	0	
Uth, K. and R. Sleigh (2014). "Deregulation of the circadian clock constitutes a significant factor in tumorigenesis: a clockwork cancer. Part II. In vivo studies." Biotechnology & Biotechnological Equipment 28(3): 379-386.	1	0	0	0	0	
van Drongelen, A., et al. (2011). "The effects of shift work on body weight change - a systematic review of longitudinal studies." Scandinavian Journal of Work Environment & Health 37(4): 263-275.	1	0	0	0	0	
Versteeg, R. I., et al. (2015). "SEROTONIN, A POSSIBLE INTERMEDIATE BETWEEN DISTURBED CIRCADIAN RHYTHMS AND METABOLIC DISEASE." Neuroscience 301: 155-167.	1	0	0	0	0	
Vieira, E., et al. (2015). "Role of the clock gene Rev-erb in metabolism and in the endocrine pancreas." Diabetes Obesity & Metabolism 17: 106-114.	1	0	0	0	0	
Vogel, M., et al. (2012). "The effects of shift work on physical and mental health." Journal of Neural Transmission 119(10): 1121-1132.	1	1	1	1	0	NO EVALUATION OF THE ASSOCIATION BETWEEN SW AND OUTCOME

Wajid, F., et al. (2020). "Therapeutic potential of melatonin as a chronobiotic and cytoprotective agent in diabetes mellitus." Journal of Diabetes and Metabolic Disorders 19(2): 1797-1825.	1	0	0	0	0	
Watanabe, K., et al. (2018). "Work-related psychosocial factors and metabolic syndrome onset among workers: a systematic review and meta-analysis." Obesity Reviews 19(11): 1557-1568.	1	1	0	0	0	
Westerterp-Plantenga, M. S. (2016). "Sleep, circadian rhythm and body weight: parallel developments." Proceedings of the Nutrition Society 75(4): 431-439.	1	0	0	0	0	
Woller, A. and D. Gonze (2021). "Circadian Misalignment and Metabolic Disorders: A Story of Twisted Clocks." Biology-Basel 10(3).	1	0	0	0	0	
Yang, Y. X. and J. F. Zhang (2020). "Bile acid metabolism and circadian rhythm." American Journal of Physiology-Gastrointestinal and Liver Physiology 319(5): G549-G563.	1	0	0	0	0	
Young, M. E. (2006). "The circadian clock within the heart: potential influence on myocardial gene expression, metabolism, and function." American Journal of Physiology-Heart and Circulatory Physiology 290(1): H1-H16.	1	0	0	0	0	
Zanquetta, M. M., et al. (2010). "Body weight, metabolism and clock genes." Diabetology & Metabolic Syndrome 2.	1	0	0	0	0	
Zarrinpar, A., et al. (2016). "Daily Eating Patterns and Their Impact on Health and Disease." Trends in Endocrinology and Metabolism 27(2): 69-83.	1	0	0	0	0	
Zelinski, E. L., et al. (2014). "The trouble with circadian clock dysfunction: Multiple deleterious effects on the brain and body." Neuroscience and Biobehavioral Reviews 40: 80-101.	1	0	0	0	0	
Zimberg, I. Z., et al. (2012). "Metabolic impact of shift work." Work-a Journal of Prevention Assessment & Rehabilitation 41: 4376-4383.	1	1	1	1	0	NARRATIVE
Zimberg, I. Z., et al. (2012). "Short sleep duration and obesity: mechanisms and future perspectives." Cell Biochemistry and Function 30(6): 524-529.	1	0	0	0	0	
Zimmerman, F. H. (2012). "Cardiovascular Disease and Risk Factors in Law Enforcement Personnel: A Comprehensive Review." Cardiology in Review 20(4): 159-166.	1	1	0	0	0	

Zmrzljak, U. P. and D. Rozman (2012). "Circadian Regulation of the Hepatic Endobiotic and Xenobitoic Detoxification Pathways: The Time Matters." Chemical Research in Toxicology 25(4): 811-824.	1	0	0	0	0	

LIPID DISORDERS	IDENTIFICATION	SCREENING		ELIGIBILITY	INCLUDED	REASON FOR EXCLUSION
	SEARCH / REFERENCE LIST	TITLE	ABSTRACT	FULL TEXT	FULL TEST	
Arendt, J. (2012). "Biological Rhythms During Residence in Polar Regions." Chronobiol Int 29(4): 379-394.	1	0	0	0	0	
Blask, D. E., et al. (2011). "Circadian regulation of molecular, dietary, and metabolic signaling mechanisms of human breast cancer growth by the nocturnal melatonin signal and the consequences of its disruption by light at night." Journal of Pineal Research 51(3): 259-269.	1	0	0	0	0	
Boivin, D. B. and F. O. James (2005). "Light treatment and circadian adaptation to shift work." Industrial Health 43(1): 34-48.	1	0	0	0	0	
Bonham, M. P., et al. (2019). "Effect of Night Time Eating on Postprandial Triglyceride Metabolism in Healthy Adults: A Systematic Literature Review." J Biol Rhythms 34(2): 119-130.	1	0	0	0	0	
Brubakk, A. O., et al. (2014). "Saturation Diving; Physiology and Pathophysiology." Compr Physiol 4(3): 1229-1272.	1	0	0	0	0	
Canuto, R., et al. (2013). "Metabolic syndrome and shift work: A systematic review." Sleep Med Rev 17(6): 425-431.	1	1	0	0	0	
Chao, H.-W., et al. (2019). "Homeostasis of Glucose and Lipid in Non-Alcoholic Fatty Liver Disease." International Journal of Molecular Sciences 20(2).	1	0	0	0	0	
de Goede, P., et al. (2018). "Circadian rhythms in mitochondrial respiration." Journal of Molecular Endocrinology 60(3): R115-R130.	1	0	0	0	0	
Duez, H. and B. Staels (2009). "Rev-erb-alpha: an integrator of circadian rhythms and metabolism." Journal of Applied Physiology 107(6): 1972-1980.	1	0	0	0	0	
Fatima, N. and S. Rana (2020). "Metabolic implications of circadian disruption." Pflugers Archiv-European Journal of Physiology 472(5): 513-526.	1	0	0	0	0	
Ferrell, J. M. and J. Y. L. Chiang (2015). "Circadian rhythms in liver metabolism and disease." Acta Pharmaceutica Sinica B 5(2): 113-122.	1	0	0	0	0	
Gooley, J. J. (2016). "Circadian regulation of lipid metabolism." Proceedings of the Nutrition Society 75(4): 440-450.	1	1	0	0	0	

Gooley, J. J. and E. C.-P. Chua (2014). "Diurnal Regulation of Lipid Metabolism and Applications of Circadian Lipidomics." Journal of Genetics and Genomics 41(5): 231-250.	1	0	0	0	0	
Hansen, A. M., et al. (2009). "A Review of the Effect of the Psychosocial Working Environment on Physiological Changes in Blood and Urine." Basic Clin Pharmacol Toxicol 105(2): 73-83.	1	1	1	1	0	NO EVALUATION OF THE ASSOCIATION BETWEEN SW AND OUTCOME
Ivanov, D. O., et al. (2020). "The Role of Prenatal Melatonin in the Regulation of Childhood Obesity." Biology-Basel 9(4).	1	0	0	0	0	
Jha, P. K., et al. (2015). "Circadian rhythms in glucose and lipid metabolism in nocturnal and diurnal mammals." Mol Cell Endocrinol 418: 74-88.	1	0	0	0	0	
Johnston, J. D. (2014). "Physiological responses to food intake throughout the day." Nutr Res Rev 27(1): 107-118.	1	0	0	0	0	
Kiehn, J.-T., et al. (2017). "Circadian Rhythms in Adipose Tissue Physiology." Compr Physiol 7(2): 383-427.	1	1	0	0	0	
Knutsson, A. (1989). "Shift work and coronary heart disease." Scand J Soc Med Suppl 44: 1-36.	1	1	1	1	0	NO EVALUATION OF THE ASSOCIATION BETWEEN SW AND OUTCOME
Kovac, U., et al. (2019). "Oxysterols and Gastrointestinal Cancers Around the Clock." Frontiers in Endocrinology 10.	1	0	0	0	0	
Landry, G. J. and T. Liu-Ambrose (2014). "Buying time: a rationale for examining the use of circadian rhythm and sleep interventions to delay progression of mild cognitive impairment to Alzheimer's disease." Frontiers in Aging Neuroscience 6.	1	0	0	0	0	
Loaiza, N., et al. (2017). "Novel regulators of plasma lipid levels." Current Opinion in Lipidology 28(3): 231-240.	1	0	0	0	0	
Matveeva, O., et al. (2018). "Western lifestyle and immunopathology of multiple sclerosis." Ann N Y Acad Sci 1417(1): 71-86.	1	0	0	0	0	
Morgan, L., et al. (2003). "Circadian aspects of postprandial metabolism." Chronobiol Int 20(5): 795-808.	1	1	1	1	0	MECHANISTIC
Opperhuizen, A.-L., et al. (2015). "Rodent models to study the metabolic effects of shiftwork in humans." Frontiers in Pharmacology 6.	1	0	0	0	0	
Paschos, G. K., et al. (2012). "Obesity in mice with adipocyte-specific deletion of clock component Arntl." Nature Medicine 18(12): 1768-+.	1	0	0	0	0	

Peter, R. and J. Siegrist (2000). "Psychosocial work environment and the risk of coronary heart disease." International Archives of Occupational and Environmental Health 73: S41-S45.	1	1	1	1	0	NO EVALUATION OF THE ASSOCIATION BETWEEN SW AND OUTCOME
Phoi, Y. Y. and J. B. Keogh (2019). "Dietary Interventions for Night Shift Workers: A Literature Review." Nutrients 11(10).	1	1	1	1	0	NO EVALUATION OF THE ASSOCIATION BETWEEN SW AND OUTCOME
Pot, G. K., et al. (2016). "Meal irregularity and cardiometabolic consequences: results from observational and intervention studies." Proceedings of the Nutrition Society 75(4): 475-486.	1	1	1	1	0	NO EVALUATION OF THE ASSOCIATION BETWEEN SW AND OUTCOME
Puttonen, S., et al. (2010). "Shift work and cardiovascular disease - pathways from circadian stress to morbidity." Scandinavian Journal of Work Environment & Health 36(2): 96-108.	1	1	1	1	0	NO EVALUATION OF THE ASSOCIATION BETWEEN SW AND OUTCOME
Reilly, T. and J. Waterhouse (2007). "Altered sleep-wake cycles and food intake: The Ramadan model." Physiology & Behavior 90(2-3): 219-228.	1	0	0	0	0	
Sahar, S. and P. Sassone-Corsi (2012). "Regulation of metabolism: the circadian clock dictates the time." Trends in Endocrinology and Metabolism 23(1): 1-8.	1	0	0	0	0	
Santana-Herrera, J., et al. (2014). "Turnos de trabajo: ¿un factor de riesgo cardiovascular?" Medicina y Seguridad del Trabajo 60(234): 179-197.	1	0	0	0	0	
Slanovic-Kuzmanovic, Z., et al. (2013). "ENDOCRINE, LIFESTYLE, AND GENETIC FACTORS IN THE DEVELOPMENT OF METABOLIC SYNDROME." Arhiv Za Higijenu Rada I Toksikologiju-Archives of Industrial Hygiene and Toxicology 64(4): 581-591.	1	0	0	0	0	
Tanner, C. M. (2010). "Advances in Environmental Epidemiology." Movement Disorders 25(3): S58-S62.	1	0	0	0	0	
Waldman, H. S., et al. (2020). "Time-restricted feeding for the prevention of cardiometabolic diseases in high-stress occupations: a mechanistic review." Nutrition Reviews 78(6): 459-464.	1	1	0	0	0	
Yasutake, K., et al. (2014). "Dietary habits and behaviors associated with nonalcoholic fatty liver disease." World Journal of Gastroenterology 20(7): 1756-1767.	1	0	0	0	0	

BEING OVERWEIGHT	IDENTIFICATION	SCREENING		ELIGIBILITY	INCLUSION	REASON FOR EXCLUSION
	SEARCH / REFERENCE LIST	TITLE	ABSTRACT	FULL TEXT	FULL TEXT	
Alfredsson, L. and T. Olsson (2019). "Lifestyle and Environmental Factors in Multiple Sclerosis." Cold Spring Harbor Perspectives in Medicine 9(4).	1	0	0	0	0	
Allison, K. C. and N. Goel (2018). "Timing of eating in adults across the weight spectrum: Metabolic factors and potential circadian mechanisms." Physiology & Behavior 192: 158-166.	1	0	0	0	0	
Andriessen, C., et al. (2021). "The importance of 24-h metabolism in obesity-related metabolic disorders: opportunities for timed interventions." International Journal of Obesity 45(3): 479-490.	1	0	0	0	0	
Angeles Bonmati-Carrion, M., et al. (2014). "Protecting the Melatonin Rhythm through Circadian Healthy Light Exposure." Int J Mol Sci 15(12): 23448-23500.	1	0	0	0	0	
Anothaisintawee, T., et al. (2016). "Sleep disturbances compared to traditional risk factors for diabetes development: Systematic review and meta-analysis." Sleep Med Rev 30: 11-24.	1	1	1	1	0	NO EVALUATION OF THE ASSOCIATION BETWEEN SW AND OUTCOME
Arble, D. M., et al. (2010). "Circadian disruption and metabolic disease: Findings from animal models." Best Practice & Research Clinical Endocrinology & Metabolism 24(5): 785-800.	1	0	0	0	0	
Ashby, T. and M. Louis (2019). "Circadian Misalignment and Cardiovascular Risk." Cardiovascular Innovations and Applications 3(4): 435-440.	1	1	1	1	0	NARRATIVE
Atkinson, G. and D. Davenne (2007). "Relationships between sleep, physical activity and human health." Physiology & Behavior 90(2-3): 229-235.	1	0	0	0	0	
Atkinson, G., et al. (2008). "Exercise, energy balance and the shift worker." Sports Medicine 38(8): 671-685.	1	1	1	1	0	NO EVALUATION OF THE ASSOCIATION BETWEEN SW AND OUTCOME
Azmi, N. A. S. M., et al. (2020). "Consequences of Circadian Disruption in Shift Workers on Chrononutrition and their Psychosocial Well-Being." International Journal of Environmental Research and Public Health 17(6).	1	1	0	0	0	
Bae, S.-A., et al. (2019). "At the Interface of Lifestyle, Behavior, and Circadian Rhythms: Metabolic Implications." Frontiers in Nutrition 6.	1	1	1	1	0	NO EVALUATION OF THE ASSOCIATION BETWEEN SW AND OUTCOME

Bailey, S. M., et al. (2014). "Circadian regulation of metabolism." Journal of Endocrinology 222(2): R75-R96.	1	1	0	0	0	
Ball, L. J., et al. (2016). "The Pathophysiologic Role of Disrupted Circadian and Neuroendocrine Rhythms in Breast Carcinogenesis." Endocr Rev 37(5): 450-466.	1	0	0	0	0	
Baron, K. G. and K. J. Reid (2014). "Circadian misalignment and health." International Review of Psychiatry 26(2): 139-154.	1	1	0	0	0	
Bass, J. and J. S. Takahashi (2010). "Circadian Integration of Metabolism and Energetics." Science 330(6009): 1349-1354.	1	0	0	0	0	
Bayon, V. and D. Leger (2014). "Occupational diseases and night-shift work." Rev Prat 64(3): 363-368.	1	1	1	1	0	NARRATIVE
Bayon, V., et al. (2014). "Sleep debt and obesity." Ann Med 46(5): 264-272.	1	1	1	1	0	NO EVALUATION OF THE ASSOCIATION BETWEEN SW AND OUTCOME
Bishehsari, F., et al. (2020). "Circadian rhythms and the gut microbiota: from the metabolic syndrome to cancer." Nature Reviews Endocrinology 16(12): 731-739.	1	0	0	0	0	
Boga, J. A., et al. (2019). "Therapeutic potential of melatonin related to its role as an autophagy regulator: A review." J Pineal Res 66(1).	1	0	0	0	0	
Bonham, M. P., et al. (2016). "Energy intake of shift workers compared to fixed day workers: A systematic review and meta-analysis." Chronobiol Int 33(8): 1086-1100.	1	1	0	0	0	
Booker, L. A., et al. (2018). "Individual vulnerability to insomnia, excessive sleepiness and shift work disorder amongst healthcare shift workers. A systematic review." Sleep Med Rev 41: 220-233.	1	1	0	0	0	
Borel, A.-L. (2019). "Sleep Apnea and Sleep Habits: Relationships with Metabolic Syndrome." Nutrients 11(11).	1	1	0	0	0	
Bottalico, L. N. and A. M. Weljie (2021). "Cross-species physiological interactions of endocrine disrupting chemicals with the circadian clock." General and Comparative Endocrinology 301.	1	0	0	0	0	
Broussard, J. and M. J. Brady (2010). "The impact of sleep disturbances on adipocyte function and lipid metabolism." Best Practice & Research Clinical Endocrinology & Metabolism 24(5): 763-773.	1	0	0	0	0	

Broussard, J. L. and E. Van Cauter (2016). "Disturbances of sleep and circadian rhythms: novel risk factors for obesity." Current Opinion in Endocrinology Diabetes and Obesity 23(5): 353-359.	1	1	1	1	0	NO EVALUATION OF THE ASSOCIATION BETWEEN SW AND OUTCOME
Broussard, J. L. and S. Devkota (2016). "The changing microbial landscape of Western society: Diet, dwellings and discordance." Molecular Metabolism 5(9): 737-742.	1	0	0	0	0	
Brzecka, A., et al. (2020). "The Association of Sleep Disorders, Obesity and Sleep-Related Hypoxia with Cancer." Current Genomics 21(6): 444-453.	1	0	0	0	0	
Buijs, R. M., et al. (2013). "The circadian system and the balance of the autonomic nervous system." Handb Clin Neurol 117: 173-191.	1	0	0	0	0	
Buss, J. (2012). "Associations Between Obesity and Stress and Shift Work Among Nurses." Workplace Health Saf 60(10): 453-458.	1	1	1	1	0	NO EVALUATION OF THE ASSOCIATION BETWEEN SW AND OUTCOME
Campagna, M., et al. (2016). "Metabolomic patterns associated to QTc interval in shiftworkers: an explorative analysis." Biomarkers 21(7): 607-613.	1	1	0	0	0	
Canuto, R., et al. (2013). "Metabolic syndrome and shift work: A systematic review." Sleep Med Rev 17(6): 425-431.	1	1	1	1	0	NO EVALUATION OF THE ASSOCIATION BETWEEN SW AND OUTCOME
Capers, P. L., et al. (2015). "A systemic review and meta-analysis of randomized controlled trials of the impact of sleep duration on adiposity and components of energy balance." Obesity Reviews 16(9): 771-782.	1	1	0	0	0	
Cappuccio, F. P. and M. A. Miller (2017). "Sleep and Cardio-Metabolic Disease." Curr Cardiol Rep 19(11).	1	1	1	1	0	NO EVALUATION OF THE ASSOCIATION BETWEEN SW AND OUTCOME
Carroll, R. G., et al. (2019). "Immunometabolism around the Clock." Trends in Molecular Medicine 25(7): 612-625.	1	0	0	0	0	
Caruso, C. C. (2014). "Negative Impacts of Shiftwork and Long Work Hours." Rehabilitation Nursing 39(1): 16-25.	1	1	1	1	0	NO EVALUATION OF THE ASSOCIATION BETWEEN SW AND OUTCOME
Caruso, C. C. (2015). "Reducing Risks to Women Linked to Shift Work, Long Work Hours, and Related Workplace Sleep and Fatigue Issues." Journal of Womens Health 24(10): 789-794.	1	1	0	0	0	
Cayanan, E. A., et al. (2019). "Is 24-hour energy intake greater during night shift compared to non-night shift patterns? A systematic review." Chronobiol Int 36(12): 1599-1612.	1	1	0	0	0	

Cermakian, N. and D. B. Boivin (2009). "The regulation of central and peripheral circadian clocks in humans." Obesity Reviews 10: 25-36.	1	0	0	0	0	
Chaix, A., et al. (2019). Time-Restricted Eating to Prevent and Manage Chronic Metabolic Diseases. Annual Review of Nutrition, Vol 39, 2019. P. J. Stover and R. Balling. 39: 291-315.	1	0	0	0	0	
Challet, E. (2013). Circadian Clocks, Food Intake, and Metabolism. Chronobiology: Biological Timing in Health and Diesease. M. U. Gillette. 119: 105-135.	1	0	0	0	0	
Challet, E. (2015). "Keeping circadian time with hormones." Diabetes Obesity & Metabolism 17: 76-83.	1	0	0	0	0	
Chappel, S. E., et al. (2017). "Nurses' occupational physical activity levels: A systematic review." International Journal of Nursing Studies 73: 52-62.	1	1	0	0	0	
Charlot, A., et al. (2021). "Beneficial Effects of Early Time-Restricted Feeding on Metabolic Diseases: Importance of Aligning Food Habits with the Circadian Clock." Nutrients 13(5).	1	0	0	0	0	
Cherrie, J. W., et al. (2018). "A review of the impact of shift-work on cancer: summary of the evidence for practitioners." Policy and Practice in Health and Safety 16(1): 145-151.	1	0	0	0	0	
Cipolla-Neto, J., et al. (2014). "Melatonin, energy metabolism, and obesity: a review." J Pineal Res 56(4): 371-381.	1	1	0	0	0	
Clougherty, J. E., et al. (2010). Work and its role in shaping the social gradient in health. Biology of Disadvantage: Socioeconomic Status and Health. N. E. Adler and J. Stewart. 1186: 102-124.	1	1	0	0	0	
Covassin, N., et al. (2016). "Keeping Up With the Clock: Circadian Disruption and Obesity Risk." Hypertension 68(5): 1081-1090.	1	1	0	0	0	
Crispim, C. A., et al. (2007). "Relation between sleep and obesity: A literature review." Arquivos Brasileiros De Endocrinologia E Metabologia 51(7): 1041-1049.	1	1	0	0	0	
Crowther, M. E., et al. (2021). "Non-Pharmacological Interventions to Improve Chronic Disease Risk Factors and Sleep in Shift Workers: A Systematic Review and Meta-Analysis." Clocks & Sleep 3(1): 132-178.	1	0	0	0	0	
Daly, A. A., et al. (2021). "A Review of Modifiable Risk Factors in Young Women for the Prevention of Breast Cancer." Breast Cancer-Targets and Therapy 13: 241-257.	1	0	0	0	0	

Davis, S. and D. K. Mirick (2006). "Circadian disruption, shift work and the risk of cancer: A summary of the evidence and studies in Seattle." Cancer Causes & Control 17(4): 539-545.	1	0	0	0	0	
Demou, E., et al. (2018). "Group-based healthy lifestyle workplace interventions for shift workers: a systematic review." Scandinavian Journal of Work Environment & Health 44(6): 568-584.	1	1	1	1	0	NO EVALUATION OF THE ASSOCIATION BETWEEN SW AND OUTCOME
Depner, C. M., et al. (2014). "Metabolic Consequences of Sleep and Circadian Disorders." Curr Diab Rep 14(7).	1	1	0	0	0	
Dibner, C. and U. Schibler (2015). "Circadian timing of metabolism in animal models and humans." Journal of Internal Medicine 277(5): 513-527.	1	0	0	0	0	
Ding, C., et al. (2018). "Sleep and Obesity." Journal of Obesity & Metabolic Syndrome 27(1): 4-24.	1	1	1	1	0	NO EVALUATION OF THE ASSOCIATION BETWEEN SW AND OUTCOME
Ding, L. and X.-H. Xiao (2020). "Gut microbiota: closely tied to the regulation of circadian clock in the development of type 2 diabetes mellitus." Chinese Medical Journal 133(7): 817-825.	1	0	0	0	0	
Dollet, L. and J. R. Zierath (2019). "Interplay between diet, exercise and the molecular circadian clock in orchestrating metabolic adaptations of adipose tissue." Journal of Physiology-London 597(6): 1439-1450.	1	0	0	0	0	
Duez, H. and B. Staels (2008). "The nuclear receptors Rev-erbs and RORs integrate circadian rhythms and metabolism." Diabetes & Vascular Disease Research 5(2): 82-88.	1	0	0	0	0	
Duez, H. and B. Staels (2010). "Nuclear Receptors Linking Circadian Rhythms and Cardiometabolic Control." Arteriosclerosis Thrombosis and Vascular Biology 30(8): 1529-1534.	1	0	0	0	0	
Duez, H., et al. (2013). "Circadian rhythmicity and metabolism: integration of metabolic and environmental signals." M S-Medecine Sciences 29(8-9): 772-777.	1	0	0	0	0	
Eckel-Mahan, K. and P. Sassone-Corsi (2013). "METABOLISM AND THE CIRCADIAN CLOCK CONVERGE." Physiological Reviews 93(1): 107-135.	1	0	0	0	0	
Eckel-Mahan, K. and P. Sassone-Corsi (2013). Epigenetic Regulation of the Molecular Clockwork. Chronobiology: Biological Timing in Health and Diesease. M. U. Gillette. 119: 29-50.	1	0	0	0	0	

Ekmekcioglu, C. and Y. Touitou (2011). "Chronobiological aspects of food intake and metabolism and their relevance on energy balance and weight regulation." Obesity Reviews 12(1): 14-25.	1	0	0	0	0	
Engin, A. (2017). "Circadian Rhythms in Diet-Induced Obesity." Adv Exp Med Biol 960: 19-52.	1	0	0	0	0	
Evans, J. A. and A. J. Davidson (2013). Health Consequences of Circadian Disruption in Humans and Animal Models. Chronobiology: Biological Timing in Health and Diesease. M. U. Gillette. 119: 283-323.	1	1	0	0	0	
Evans, M. C. and G. M. Anderson (2018). "Integration of Circadian and Metabolic Control of Reproductive Function." Endocrinology 159(11): 3661-3673.	1	0	0	0	0	
Fatima, N. and S. Rana (2020). "Metabolic implications of circadian disruption." Pflugers Archiv-European Journal of Physiology 472(5): 513-526.	1	0	0	0	0	
Ferrell, J. M. and J. Y. L. Chiang (2015). "Circadian rhythms in liver metabolism and disease." Acta Pharmaceutica Sinica B 5(2): 113-122.	1	0	0	0	0	
Ferri, G. M., et al. (2019). "Healthy Diet and Reduction of Chronic Disease Risks of Night Shift Workers." Current Medicinal Chemistry 26(19): 3521-3541.	1	1	0	0	0	
Fonken, L. K. and R. J. Nelson (2014). "The Effects of Light at Night on Circadian Clocks and Metabolism." Endocr Rev 35(4): 648-670.	1	0	0	0	0	
Forrestel, A. C., et al. (2017). "Chronomedicine and type 2 diabetes: shining some light on melatonin." Diabetologia 60(5): 808-822.	1	0	0	0	0	
Fossum, I. N., et al. (2013). "Effects of Shift and Night Work in the Offshore Petroleum Industry: A Systematic Review." Ind Health 51(5): 530-544.	1	1	1	1	0	NO EVALUATION OF THE ASSOCIATION BETWEEN SW AND OUTCOME
Frazier, K., et al. (2020). "Mediators of Host-Microbe Circadian Rhythms in Immunity and Metabolism." Biology-Basel 9(12).	1	0	0	0	0	
Frost, P., et al. (2009). "Shift work and the risk of ischemic heart disease - a systematic review of the epidemiologic evidence." Scandinavian Journal of Work Environment & Health 35(3): 163-179.	1	1	0	0	0	
Funderburk, L., et al. (2020). "Healthy Behaviors through Behavioral Design-Obesity Prevention." International Journal of Environmental Research and Public Health 17(14).	1	0	0	0	0	

Gallant, A. R., et al. (2012). "The night-eating syndrome and obesity." Obesity Reviews 13(6): 528-536.	1	1	0	0	0	
Gan, Y., et al. (2018). "Association between shift work and risk of prostate cancer: a systematic review and meta-analysis of observational studies." Carcinogenesis 39(2): 87-97.	1	0	0	0	0	
Gangwisch, J. E. (2009). "Epidemiological evidence for the links between sleep, circadian rhythms and metabolism." Obesity Reviews 10: 37-45.	1	1	0	0	0	
Gao, Y., et al. (2020). "Association between shift work and risk of type 2 diabetes mellitus: a systematic review and dose-response meta-analysis of observational studies." Chronobiol Int 37(1): 29-46.	1	0	0	0	0	
Garaulet, M. and J. A. Madrid (2009). "Chronobiology, genetics and metabolic syndrome." Current Opinion in Lipidology 20(2): 127-134.	1	0	0	0	0	
Garaulet, M. and P. Gomez-Abellan (2013). "Chronobiology and obesity." Nutr Hosp 28: 114-120.	1	0	0	0	0	
Garaulet, M., et al. (2010). "The chronobiology, etiology and pathophysiology of obesity." International Journal of Obesity 34(12): 1667-1683.	1	0	0	0	0	
Gerhart-Hines, Z. and M. A. Lazar (2015). "Circadian Metabolism in the Light of Evolution." Endocr Rev 36(3): 289-304.	1	0	0	0	0	
Gillie, O. (2010). "Sunlight robbery: A critique of public health policy on vitamin D in the UK." Molecular Nutrition & Food Research 54(8): 1148-1163.	1	0	0	0	0	
Gohari, A., et al. (2021). "Shift working and cardiovascular health." Chronobiology International.	1	1	0	0	0	
Golubnitschaja, O., et al. (2016). "Breast cancer epidemic in the early twenty-first century: evaluation of risk factors, cumulative questionnaires and recommendations for preventive measures." Tumor Biology 37(10): 12941-12957.	1	0	0	0	0	
Gonnissen, H. K. J., et al. (2013). "Chronobiology, endocrinology, and energy- and food-reward homeostasis." Obesity Reviews 14(5): 405-416.	1	0	0	0	0	
Gonzalez-Gonzalez, A., et al. (2018). "Melatonin: A Molecule for Reducing Breast Cancer Risk." Molecules 23(2).	1	0	0	0	0	
Gooley, J. J. (2016). "Circadian regulation of lipid metabolism." Proceedings of the Nutrition Society 75(4): 440-450.	1	0	0	0	0	

Gooley, J. J. and E. C.-P. Chua (2014). "Diurnal Regulation of Lipid Metabolism and Applications of Circadian Lipidomics." Journal of Genetics and Genomics 41(5): 231-250.	1	0	0	0	0	
Guandalini, L. S., et al. (2020). "Analysis of the evidence of related factors, associated conditions and at-risk populations of the NANDA-I nursing diagnosis insomnia." International Journal of Nursing Sciences 7(4): 376-386.	1	0	0	0	0	
Guerrero-Vargas, N. N., et al. (2018). "Shift-work: is time of eating determining metabolic health? Evidence from animal models." Proceedings of the Nutrition Society 77(3): 199-215.	1	0	0	0	0	
Hardeland, R. (2014). "Melatonin, Noncoding RNAs, Messenger RNA Stability and Epigenetics-Evidence, Hints, Gaps and Perspectives." Int J Mol Sci 15(10): 18221-18252.	1	0	0	0	0	
Hatori, M. and S. Panda (2015). Response of Peripheral Rhythms to the Timing of Food Intake. Circadian Rhythms and Biological Clocks, Pt B. A. Sehgal. 552: 145-161.	1	0	0	0	0	
Haus, E. L. and M. H. Smolensky (2013). "Shift work and cancer risk: Potential mechanistic roles of circadian disruption, light at night, and sleep deprivation." Sleep Med Rev 17(4): 273-284.	1	0	0	0	0	
Hedstrom, A. K., et al. (2015). "The Role of Environment and Lifestyle in Determining the Risk of Multiple Sclerosis." Current topics in behavioral neurosciences 26: 87-104.	1	0	0	0	0	
Hedstrom, A. K., et al. (2016). "Environmental factors and their interactions with risk genotypes in MS susceptibility." Curr Opin Neurol 29(3): 293-298.	1	0	0	0	0	
Herichova, I. (2013). "Changes of physiological functions induced by shift work." Endocr Regul 47(3): 159-170.	1	1	1	1	0	NO EVALUATION OF THE ASSOCIATION BETWEEN SW AND OUTCOME
Hittle, B. M. and G. L. Gillespie (2018). "Identifying shift worker chronotype: implications for health." Ind Health 56(6): 512-523.	1	1	1	1	0	NO EVALUATION OF THE ASSOCIATION BETWEEN SW AND OUTCOME
Hoogerwerf, W. A. (2009). "Role of biological rhythms in gastrointestinal health and disease." Rev Endocr Metab Disord 10(4): 293-300.	1	0	0	0	0	
Horakova, D., et al. (2018). "Risks and protective factors for triple negative breast cancer with a focus on micronutrients and infections." Biomedical Papers-Olomouc 162(2): 83-89.	1	0	0	0	0	

Hruby, A., et al. (2016). "Determinants and Consequences of Obesity." Am J Public Health 106(9): 1656-1662.	1	1	1	1	0	NO EVALUATION OF THE ASSOCIATION BETWEEN SW AND OUTCOME
Ikegami, K., et al. (2019). "Interconnection between circadian clocks and thyroid function." Nature Reviews Endocrinology 15(10): 590-600.	1	0	0	0	0	
Ivanov, D. O., et al. (2020). "The Role of Prenatal Melatonin in the Regulation of Childhood Obesity." Biology-Basel 9(4).	1	0	0	0	0	
Jha, P. K., et al. (2015). "Circadian rhythms in glucose and lipid metabolism in nocturnal and diurnal mammals." Mol Cell Endocrinol 418: 74-88.	1	0	0	0	0	
Jiang, P. and F. W. Turek (2018). "The endogenous circadian clock programs animals to eat at certain times of the 24-hour day: What if we ignore the clock?" Physiology & Behavior 193: 211-217.	1	0	0	0	0	
Johnston, J. D., et al. (2016). "Circadian Rhythms, Metabolism, and Chrononutrition in Rodents and Humans." Advances in Nutrition 7(2): 399-406.	1	0	0	0	0	
Kales, S. N., et al. (2009). "Blood-Pressure in Firefighters, Police Officers, and Other Emergency Responders." American Journal of Hypertension 22(1): 11-20.	1	1	0	0	0	
Kanikowska, D., et al. (2015). "Contribution of daily and seasonal biorhythms to obesity in humans." Int J Biometeorol 59(4): 377-384.	1	1	1	1	0	NO EVALUATION OF THE ASSOCIATION BETWEEN SW AND OUTCOME
Karatsoreos, I. N. (2012). "Effects of Circadian Disruption on Mental and Physical Health." Current Neurology and Neuroscience Reports 12(2): 218-225.	1	1	0	0	0	
Kelly, M. and J. Wills (2018). "Systematic review: What works to address obesity in nurses?" Occupational Medicine-Oxford 68(4): 228-238.	1	1	1	1	0	NO EVALUATION OF THE ASSOCIATION BETWEEN SW AND OUTCOME
Kennedy, S. H. (1994). "MELATONIN DISTURBANCES IN ANOREXIA-NERVOSA AND BULIMIA-NERVOSA." International Journal of Eating Disorders 16(3): 257-265.	1	0	0	0	0	
Kenny, G. P., et al. (2016). "Age, human performance, and physical employment standards." Applied Physiology Nutrition and Metabolism 41(6): S92-S107.	1	0	0	0	0	
Kessler, K. and O. Pivovarova-Ramich (2019). "Meal Timing, Aging, and Metabolic Health." Int J Mol Sci 20(8).	1	0	0	0	0	
Khan, S., et al. (2018). "Shiftwork-Mediated Disruptions of Circadian Rhythms and Sleep Homeostasis Cause Serious Health Problems." International Journal of Genomics.	1	1	1	1	0	NO EVALUATION OF THE ASSOCIATION BETWEEN SW AND OUTCOME

Kiehn, J.-T., et al. (2017). "Circadian Rhythms in Adipose Tissue Physiology." Comprehensive Physiology 7(2): 383-427.	1	0	0	0	0	
Knutsson, A. (1989). "Shift work and coronary heart disease." Scand J Soc Med Suppl 44: 1-36.	1	1	0	0	0	
Kolbe, I. and H. Oster (2019). "Chronodisruption, Metabolic Homeostasis, and the Regulation of Inflammation in Adipose Tissues." Yale Journal of Biology and Medicine 92(2): 317-325.	1	0	0	0	0	
Kolbe, I., et al. (2019). "Interplay of central and peripheral circadian clocks in energy metabolism regulation." Journal of Neuroendocrinology 31(5).	1	0	0	0	0	
Konturek, P. C., et al. (2011). "GUT CLOCK: IMPLICATION OF CIRCADIAN RHYTHMS IN THE GASTOINTESTINAL TRACT." Journal of Physiology and Pharmacology 62(2): 139-150.	1	0	0	0	0	
Koren, D., et al. (2015). "Metabolic and Glycemic Sequelae of Sleep Disturbances in Children and Adults." Curr Diab Rep 15(1).	1	0	0	0	0	
Kozlowska, L., et al. (2019). "HEALTH RISK IN TRANSPORT WORKERS PART II. DIETARY COMPOUNDS AS MODULATORS OF OCCUPATIONAL EXPOSURE TO CHEMICALS." Int J Occup Med Environ Health 32(4): 441-464.	1	1	1	1	0	NO EVALUATION OF THE ASSOCIATION BETWEEN SW AND OUTCOME
Kushner, J. and T. Ruffin (2015). "Empowering a Healthy Practice Environment." Nursing Clinics of North America 50(1): 167-+.	1	0	0	0	0	
Laermans, J. and I. Depoortere (2016). "Chronobesity: role of the circadian system in the obesity epidemic." Obesity Reviews 17(2): 108-125.	1	1	1	1	0	NO EVALUATION OF THE ASSOCIATION BETWEEN SW AND OUTCOME
Leger, D., et al. (2009). "Biological clock, sleep and shift-work medical consequences." Archives Des Maladies Professionnelles Et De L Environnement 70(3): 246-252.	1	1	1	1	0	NO EVALUATION OF THE ASSOCIATION BETWEEN SW AND OUTCOME
Leger, D., et al. (2018). "Shift-workers and night-workers' health consequences: State of art and recommendations." Presse Medicale 47(11-12): 991-999.	1	1	1	1	0	NO EVALUATION OF THE ASSOCIATION BETWEEN SW AND OUTCOME
Li, H., et al. (2019). "Napping on night-shifts among nursing staff: A mixed-methods systematic review" J Adv Nurs 75(2): 291-312.	1	1	0	0	0	
Li, J., et al. (2014). "Parents' Nonstandard Work Schedules and Child Well-Being: A Critical Review of the Literature." Journal of Primary Prevention 35(1): 53-73.	1	0	0	0	0	

Li, W., et al. (2021). "Association of noise exposure with risk of metabolic syndrome: Evidence from 44,698 individuals." Diabetes research and clinical practice 178: 108944-108944.	1	0	0	0	0	
Liang, X. and G. A. FitzGerald (2017). "Timing the Microbes: The Circadian Rhythm of the Gut Microbiome." Journal of Biological Rhythms 32(6): 505-515.	1	0	0	0	0	
Madeira, S. G., et al. (2021). "The Impact of Different Types of Shift Work on Blood Pressure and Hypertension: A Systematic Review and Meta-Analysis." International Journal of Environmental Research and Public Health 18(13).	1	0	0	0	0	
Marqueze, E. C., et al. (2012). "Irregular working times and metabolic disorders among truck drivers: a review." Work-a Journal of Prevention Assessment & Rehabilitation 41: 3718-3725.	1	1	1	1	0	NO EVALUATION OF THE ASSOCIATION BETWEEN SW AND OUTCOME
Martchenko, A., et al. (2020). "Circadian Rhythms and the Gastrointestinal Tract: Relationship to Metabolism and Gut Hormones." Endocrinology 161(12).	1	0	0	0	0	
Matheson, A., et al. (2014). "The impact of shiftwork on health: a literature review." Journal of Clinical Nursing 23(23-24): 3309-3320.	1	1	1	1	0	NARRATIVE
Maury, E. (2019). "Off the Clock: From Circadian Disruption to Metabolic Disease." Int J Mol Sci 20(7).	1	0	0	0	0	
Mayeuf-Louchart, A., et al. (2017). "Circadian control of metabolism and pathological consequences of clock perturbations." Biochimie 143: 42-50.	1	0	0	0	0	
McHill, A. W. and K. P. Wright, Jr. (2017). "Role of sleep and circadian disruption on energy expenditure and in metabolic predisposition to human obesity and metabolic disease." Obesity Reviews 18: 15-24.	1	1	0	0	0	
McMahon, D. M., et al. (2018). "Persistence of social jetlag and sleep disruption in healthy young adults." Chronobiol Int 35(3): 312-328.	1	0	0	0	0	
Meiliana, A., et al. (2015). "Chronodisruption and Obesity." Indonesian Biomedical Journal 7(3): 117-128.	1	1	0	0	0	
Mendoza, J. (2019). "Food intake and addictive-like eating behaviors: Time to think about the circadian clock(s)." Neuroscience and Biobehavioral Reviews 106: 122-132.	1	0	0	0	0	
Moran-Ramos, S.-A., et al. (2016). "When to eat? The influence of circadian rhythms on metabolic health: are animal studies providing the evidence?" Nutr Res Rev 29(2): 180-193.	1	0	0	0	0	

Morris, C. J., et al. (2012). The impact of the circadian timing system on cardiovascular and metabolic function. Neurobiology of Circadian Timing. A. Kalsbeek, M. Merrow, T. Roenneberg and R. G. Foster. 199: 337-358.	1	1	1	1	0	NO EVALUATION OF THE ASSOCIATION BETWEEN SW AND OUTCOME
Mosendane, T., et al. (2008). "Shift work and its effects on the cardiovascular system." Cardiovasc J Afr 19(4): 210-215.	1	1	1	1	0	NARRATIVE
Mukherji, A., et al. (2019). "The circadian clock and liver function in health and disease." Journal of Hepatology 71(1): 200-211.	1	0	0	0	0	
Mukherji, A., et al. (2020). "Perturbation of the circadian clock and pathogenesis of NAFLD." Metabolism-Clinical and Experimental 111.	1	0	0	0	0	
Nea, F. M., et al. (2015). "Dietary and lifestyle habits and the associated health risks in shift workers." Nutr Res Rev 28(2): 143-166.	1	1	1	1	0	NARRATIVE
Nedeltcheva, A. V. and F. A. J. L. Scheer (2014). "Metabolic effects of sleep disruption, links to obesity and diabetes." Current Opinion in Endocrinology Diabetes and Obesity 21(4): 293-298.	1	0	0	0	0	
Nelson, R. J. and S. Chbeir (2018). "Dark matters: effects of light at night on metabolism." Proceedings of the Nutrition Society 77(3): 223-229.	1	0	0	0	0	
Nicholls, R., et al. (2017). "Barriers and facilitators to healthy eating for nurses in the workplace: an integrative review." J Adv Nurs 73(5): 1051-1065.	1	1	1	1	0	NO EVALUATION OF THE ASSOCIATION BETWEEN SW AND OUTCOME
Nohara, K., et al. (2015). "Manipulating the circadian and sleep cycles to protect against metabolic disease." Frontiers in Endocrinology 6.	1	0	0	0	0	
Olsson, T., et al. (2017). "Interactions between genetic, lifestyle and environmental risk factors for multiple sclerosis." Nature Reviews Neurology 13(1): 25-36.	1	0	0	0	0	
Oosterman, J. E., et al. (2020). "The Circadian Clock, Shift Work, and Tissue-Specific Insulin Resistance." Endocrinology 161(12).	1	0	0	0	0	
Opperhuizen, A.-L., et al. (2015). "Rodent models to study the metabolic effects of shiftwork in humans." Frontiers in Pharmacology 6.	1	0	0	0	0	
Orihara, K., et al. (2020). "Crosstalk Among Circadian Rhythm, Obesity and Allergy." Int J Mol Sci 21(5).	1	0	0	0	0	
Otamas, A., et al. (2020). "Diabetes and atherothrombosis: The circadian rhythm and role of melatonin in vascular protection." Diabetes & Vascular Disease Research 17(3).	1	0	0	0	0	

Pagano, E. S., et al. (2017). "White Adipose Tissue and Circadian Rhythm Dysfunctions in Obesity: Pathogenesis and Available Therapies." Neuroendocrinology 104(4): 347-363.	1	0	0	0	0	
Page, A. J. (2021). "Gastrointestinal Vagal Afferents and Food Intake: Relevance of Circadian Rhythms." Nutrients 13(3).	1	0	0	0	0	
Page, A. J., et al. (2020). "Circadian regulation of appetite and time restricted feeding." Physiology & Behavior 220.	1	0	0	0	0	
Pandalai, S. P., et al. (2013). "Conceptual heuristic models of the interrelationships between obesity and the occupational environment." Scandinavian Journal of Work Environment & Health 39(3): 221-232.	1	1	1	1	0	NO EVALUATION OF THE ASSOCIATION BETWEEN SW AND OUTCOME
Paschos, G. K. (2015). "Circadian clocks, feeding time, and metabolic homeostasis." Frontiers in Pharmacology 6.	1	0	0	0	0	
Paschos, G. K. (2021). "Diurnal rhythms and obesity." Current Opinion in Clinical Nutrition and Metabolic Care 24(4): 333-338.	1	1	0	0	0	
Paschos, G. K., et al. (2012). "Obesity in mice with adipocyte-specific deletion of clock component Arntl." Nature Medicine 18(12): 1768-+.	1	0	0	0	0	
Peplonska, B., et al. (2019). "THE ASSOCIATION BETWEEN NIGHT SHIFT WORK AND NUTRITION PATTERNS AMONG NURSES: A LITERATURE REVIEW." Medycyna Pracy 70(3): 363-376.	1	1	0	0	0	
Perez-Chada, D., et al. (2009). "DIURNAL RHYTHMS, OBESITY AND EDUCATIONAL ACHIEVEMENT IN SOUTH AMERICAN CULTURES." International Journal of Neuroscience 119(8): 1091-1104.	1	0	0	0	0	
Phoi, Y. Y. and J. B. Keogh (2019). "Dietary Interventions for Night Shift Workers: A Literature Review." Nutrients 11(10).	1	1	0	0	0	
Plano, S. A., et al. (2017). "Circadian and Metabolic Effects of Light: Implications in Weight Homeostasis and Health." Frontiers in Neurology 8.	1	0	0	0	0	
Poljsak, B. (2018). "NAMPT-Mediated NAD Biosynthesis as the Internal Timing Mechanism: In NAD plus World, Time Is Running in Its Own Way." Rejuvenation Research 21(3): 210-224.	1	0	0	0	0	
Porkka-Heiskanen, T., et al. (2013). "Sleep, its regulation and possible mechanisms of sleep disturbances." Acta Physiologica 208(4): 311-328.	1	0	0	0	0	

Porter, J., et al. (2017). "Is physiological glucocorticoid replacement important in children?" Arch Dis Child 102(2): 199-205.	1	0	0	0	0	
Pot, G. K. (2018). "Sleep and dietary habits in the urban environment: the role of chrono-nutrition." Proceedings of the Nutrition Society 77(3): 189-198.	1	0	0	0	0	
Pot, G. K., et al. (2016). "Meal irregularity and cardiometabolic consequences: results from observational and intervention studies." Proceedings of the Nutrition Society 75(4): 475-486.	1	1	0	0	0	
Prasai, M. J., et al. (2008). "Molecular clocks, type 2 diabetes and cardiovascular disease." Diabetes & Vascular Disease Research 5(2): 89-95.	1	0	0	0	0	
Puttonen, S., et al. (2010). "Shift work and cardiovascular disease - pathways from circadian stress to morbidity." Scandinavian Journal of Work Environment & Health 36(2): 96-108.	1	1	1	1	0	NARRATIVE
Qian, J. and F. A. J. L. Scheer (2016). "Circadian System and Glucose Metabolism: Implications for Physiology and Disease." Trends in Endocrinology and Metabolism 27(5): 282-293.	1	0	0	0	0	
Reutrakul, S. and K. L. Knutson (2015). "Consequences of Circadian Disruption on Cardiometabolic Health." Sleep Med Clin 10(4): 455-468.	1	1	1	1	0	NO EVALUATION OF THE ASSOCIATION BETWEEN SW AND OUTCOME
Reynolds, A. C., et al. (2017). "The shift work and health research agenda: Considering changes in gut microbiota as a pathway linking shift work, sleep loss and circadian misalignment, and metabolic disease." Sleep Med Rev 34: 3-9.	1	1	1	1	0	NO EVALUATION OF THE ASSOCIATION BETWEEN SW AND OUTCOME
Rosa, D., et al. (2020). "The relationship between urolithiasis, metabolic syndrome and nurse shift work. A literature review." International Journal of Urological Nursing 14(2): 57-66.	1	1	0	0	0	
Rueger, M. and F. A. J. L. Scheer (2009). "Effects of circadian disruption on the cardiometabolic system." Rev Endocr Metab Disord 10(4): 245-260.	1	1	1	1	0	NO EVALUATION OF THE ASSOCIATION BETWEEN SW AND OUTCOME
Rynders, C. A., et al. (2019). "Effectiveness of Intermittent Fasting and Time-Restricted Feeding Compared to Continuous Energy Restriction for Weight Loss." Nutrients 11(10).	1	0	0	0	0	
Sakamoto, Y. S., et al. (2018). "Prevalence of obstructive sleep apnea in shift workers: a systematic review." Ciencia & Saude Coletiva 23(10): 3381-3392.	1	1	1	1	0	NO EVALUATION OF THE ASSOCIATION BETWEEN SW AND OUTCOME

Santos, I., et al. (2020). "Obstructive sleep apnea, shift work and cardiometabolic risk." Sleep Medicine 74: 132-140.	1	1	0	0	0	
Schilperoort, M., et al. (2020). "Time for Novel Strategies to Mitigate Cardiometabolic Risk in Shift Workers." Trends in Endocrinology and Metabolism 31(12): 952-964.	1	1	1	1	0	NARRATIVE
Scott, C. and A. M. Johnstone (2012). "Stress and Eating Behaviour: Implications for Obesity." Obesity Facts 5(2): 277-287.	1	0	0	0	0	
Sharma, S. and M. Kavuru (2010). "Sleep and Metabolism: An Overview." International Journal of Endocrinology.	1	0	0	0	0	
Shearer, J., et al. (2016). "Nutra-ergonomics: influence of nutrition on physical employment standards and the health of workers." Applied Physiology Nutrition and Metabolism 41(6): S165-S174.	1	0	0	0	0	
Shetty, A., et al. (2018). "Role of the Circadian Clock in the Metabolic Syndrome and Nonalcoholic Fatty Liver Disease." Digestive Diseases and Sciences 63(12): 3187-3206.	1	0	0	0	0	
Shi, T., et al. (2020). "Does insomnia predict a high risk of cancer? A systematic review and meta-analysis of cohort studies." Journal of Sleep Research 29(1).	1	0	0	0	0	
Shrestha, N., et al. (2016). "The Impact of Obesity in the Workplace: a Review of Contributing Factors, Consequences and Potential Solutions." Curr Obes Rep 5(3): 344-360.	1	1	1	1	0	NARRATIVE
Slanovic-Kuzmanovic, Z., et al. (2013). "ENDOCRINE, LIFESTYLE, AND GENETIC FACTORS IN THE DEVELOPMENT OF METABOLIC SYNDROME." Arhiv Za Higijenu Rada I Toksikologiju-Archives of Industrial Hygiene and Toxicology 64(4): 581-591.	1	0	0	0	0	
Sleddens, E. F. C., et al. (2015). "Correlates of dietary behavior in adults: an umbrella review." Nutrition Reviews 73(8): 477-499.	1	0	0	0	0	
Smith, E. C., et al. (2019). "Exploring the Physical and Mental Health Challenges Associated with Emergency Service Call-Taking and Dispatching: A Review of the Literature." Prehospital and Disaster Medicine 34(6): 619-624.	1	0	0	0	0	
Soares, E. M. K. V. K., et al. (2020). "Worldwide prevalence of obesity among firefighters: a systematic review protocol." Bmj Open 10(1).	1	0	0	0	0	
Soteriades, E. S., et al. (2011). "Cardiovascular Disease in US Firefighters A Systematic Review." Cardiol Rev 19(4): 202-215.	1	1	0	0	0	

Stevens, R. G. and Y. Zhu (2015). "Electric light, particularly at night, disrupts human circadian rhythmicity: is that a problem?" Philosophical Transactions of the Royal Society B-Biological Sciences 370(1667).	1	0	0	0	0	
Sun, X., et al. (2020). "The association between sleep chronotype and obesity among black and white participants of the Bogalusa Heart Study." Chronobiol Int 37(1): 123-134.	1	0	0	0	0	
Tamashiro, K. L., et al. (2011). "Chronic stress, metabolism, and metabolic syndrome." Stress-the International Journal on the Biology of Stress 14(5): 468-474.	1	0	0	0	0	
Thorpy, M. J., et al. (2007). "Patient-management strategies." American Journal of Managed Care 13(6): S140-S147.	1	0	0	0	0	
Tirabassi, G., et al. (2014). "Harmful effects of functional hypercortisolism: a working hypothesis." Endocrine 46(3): 370-386.	1	0	0	0	0	
Touitou, Y., et al. (2017). "Association between light at night, melatonin secretion, sleep deprivation, and the internal clock: Health impacts and mechanisms of circadian disruption." Life Sci 173: 94-106.	1	0	0	0	0	
Versteeg, R. I., et al. (2015). "SEROTONIN, A POSSIBLE INTERMEDIATE BETWEEN DISTURBED CIRCADIAN RHYTHMS AND METABOLIC DISEASE." Neuroscience 301: 155-167.	1	0	0	0	0	
Vieira, E., et al. (2015). "Role of the clock gene Rev-erb in metabolism and in the endocrine pancreas." Diabetes Obesity & Metabolism 17: 106-114.	1	0	0	0	0	
Viswanathan, A. N. and E. S. Schernhammer (2009). "Circulating melatonin and the risk of breast and endometrial cancer in women." Cancer Lett 281(1): 1-7.	1	0	0	0	0	
Waldman, H. S., et al. (2020). "Time-restricted feeding for the prevention of cardiometabolic diseases in high-stress occupations: a mechanistic review." Nutrition Reviews 78(6): 459-464.	1	0	0	0	0	
Watanabe, K., et al. (2018). "Work-related psychosocial factors and metabolic syndrome onset among workers: a systematic review and meta-analysis." Obesity Reviews 19(11): 1557-1568.	1	1	0	0	0	
West, A. C. and D. A. Bechtold (2015). "The cost of circadian desynchrony: Evidence, insights and open questions." Bioessays 37(7): 777-788.	1	1	0	0	0	

Wester, V. L. and E. F. C. van Rossum (2015). "Clinical applications of cortisol measurements in hair." Eur J Endocrinol 173(4): M1-M10.	1	0	0	0	0	
Westerterp-Plantenga, M. S. (2016). "Sleep, circadian rhythm and body weight: parallel developments." Proceedings of the Nutrition Society 75(4): 431-439.	1	0	0	0	0	
Wittert, G. (2014). "The relationship between sleep disorders and testosterone in men." Asian Journal of Andrology 16(2): 262-265.	1	0	0	0	0	
Wolk, R. and V. K. Somers (2007). "Sleep and the metabolic syndrome." Exp Physiol 92(1): 67-78.	1	0	0	0	0	
Wolk, R., et al. (2005). "Sleep and cardiovascular disease." Curr Probl Cardiol 30(12): 625-662.	1	0	0	0	0	
Wosu, A. C., et al. (2013). "Correlates of cortisol in human hair: implications for epidemiologic studies on health effects of chronic stress." Annals of Epidemiology 23(12): 797-811.	1	0	0	0	0	
Wyse, C. A., et al. (2011). "Circadian desynchrony and metabolic dysfunction; did light pollution make us fat?" Medical Hypotheses 77(6): 1139-1144.	1	0	0	0	0	
Wyse, C. A., et al. (2014). "The bright-nights and dim-days of the urban photoperiod: Implications for circadian rhythmicity, metabolism and obesity." Ann Med 46(5): 253-263.	1	0	0	0	0	
Xu, T. and B. Lu (2019). "The effects of phytochemicals on circadian rhythm and related diseases." Critical Reviews in Food Science and Nutrition 59(6): 882-892.	1	0	0	0	0	
Yamada, Y., et al. (2002). "Prevention of weight gain and obesity in occupational populations: A new target of health promotion services at worksites." Journal of Occupational Health 44(6): 373-384.	1	1	0	0	0	
Yang, Y. and J. Zhang (2020). "Bile acid metabolism and circadian rhythm." American Journal of Physiology-Gastrointestinal and Liver Physiology 319(5): G549-G563.	1	0	0	0	0	
Yasutake, K., et al. (2014). "Dietary habits and behaviors associated with nonalcoholic fatty liver disease." World Journal of Gastroenterology 20(7): 1756-1767.	1	0	0	0	0	
Young, M. E. (2006). "The circadian clock within the heart: potential influence on myocardial gene expression, metabolism, and function." American Journal of Physiology-Heart and Circulatory Physiology 290(1): H1-H16.	1	0	0	0	0	

Yu, E., et al. (2016). "Diet, Lifestyle, Biomarkers, Genetic Factors, and Risk of Cardiovascular Disease in the Nurses' Health Studies." Am J Public Health 106(9): 1616-1623.	1	1	1	1	0	NO EVALUATION OF THE ASSOCIATION BETWEEN SW AND OUTCOME
Zanquetta, M. M., et al. (2010). "Body weight, metabolism and clock genes." Diabetology & Metabolic Syndrome 2.	1	0	0	0	0	
Zarrinpar, A., et al. (2016). "Daily Eating Patterns and Their Impact on Health and Disease." Trends in Endocrinology and Metabolism 27(2): 69-83.	1	0	0	0	0	
Zelinski, E. L., et al. (2014). "The trouble with circadian clock dysfunction: Multiple deleterious effects on the brain and body." Neuroscience and Biobehavioral Reviews 40: 80-101.	1	0	0	0	0	
Zimberg, I. Z., et al. (2012). "Metabolic impact of shift work." Work-a Journal of Prevention Assessment & Rehabilitation 41: 4376-4383.	1	1	1	1	0	NARRATIVE
Zimberg, I. Z., et al. (2012). "Short sleep duration and obesity: mechanisms and future perspectives." Cell Biochemistry and Function 30(6): 524-529.	1	0	0	0	0	
Zimmerman, F. H. (2012). "Cardiovascular Disease and Risk Factors in Law Enforcement Personnel: A Comprehensive Review." Cardiol Rev 20(4): 159-166.	1	0	0	0	0	

HYPERTENSION	IDENTIFICATION	SCREENING		ELIGIBILITY	INCLUDED	REASON FOR EXCLUSION
	SEARCH / REFERENCE LIST	TITLE	ABSTRACT	FULL TEXT	FULL TEXT	
Abbott, S. M., H. Attarian and P. C. Zee (2014). "Sleep disorders in perinatal women." Best Practice & Research Clinical Obstetrics & Gynaecology **28**(1): 159-168.	1	0	0	0	0	
Alibhai, F. J., E. V. Tsimakouridze, C. J. Reitz, W. G. Pyle and T. A. Martino (2015). "Consequences of Circadian and Sleep Disturbances for the Cardiovascular System." Canadian Journal of Cardiology **31**(7): 860-872.	1	0	0	0	0	
Ancoli-Israel, S., R. Cole, C. Alessi, M. Chambers, W. Moorcroft and C. P. Pollak (2003). "The role of actigraphy in the study of sleep and circadian rhythms." Sleep **26**(3): 342-392.	1	0	0	0	0	
Ashby, T. and M. Louis (2019). "Circadian Misalignment and Cardiovascular Risk." Cardiovascular Innovations and Applications **3**(4): 435-440.	1	1	1	1	0	NARRATIVE
Atkinson, G. and D. Davenne (2007). "Relationships between sleep, physical activity and human health." Physiology & Behavior **90**(2-3): 229-235.	1	0	0	0	0	
Atkinson, G. and T. Reilly (1996). "Circadian Variation in sports performance." Sports Medicine **21**(4): 292-312.	1	0	0	0	0	
Azmi, N. A. S. M., et al. (2020). "Consequences of Circadian Disruption in Shift Workers on Chrononutrition and their Psychosocial Well-Being." International Journal of Environmental Research and Public Health 17(6).	1	0	0	0	0	
Bass, J. and J. S. Takahashi (2010). "Circadian Integration of Metabolism and Energetics." Science **330**(6009): 1349-1354.	1	0	0	0	0	
Boggild, H. and A. Knutsson (1999). "Shift work, risk factors and cardiovascular disease." Scandinavian Journal of Work Environment & Health **25**(2): 85-99.	1	1	0	0	0	
Bollinger, T. and U. Schibler (2014). "Circadian rhythms - from genes to physiology and disease." Swiss Medical Weekly **144**.	1	0	0	0	0	
Bonny, O. and D. Firsov (2013). "Circadian regulation of renal function and potential role in hypertension." Current Opinion in Nephrology and Hypertension **22**(4): 439-444.	1	0	0	0	0	

Bonzini, M., D. Coggon and K. T. Palmer (2007). "Risk of prematurity, low birthweight and pre-eclampsia in relation to working hours and physical activities: a systematic review." Occupational and Environmental Medicine **64**(4): 228-243.	1	0	0	0	0	
Buijs, F. N., L. Leon-Mercado, M. Guzman-Ruiz, N. N. Guerrero-Vargas, F. Romo-Nava and R. M. Buijs (2016). "The Circadian System: A Regulatory Feedback Network of Periphery and Brain." Physiology **31**(3): 170-181.	1	0	0	0	0	
Buijs, R. M., et al. (2021). "The circadian system: From clocks to physiology." Handbook of clinical neurology 179: 233-247.	1	0	0	0	0	
Buijs, R., R. Salgado, E. Sabath and C. Escobar (2013). Peripheral Circadian Oscillators: Time and Food. Chronobiology: Biological Timing in Health and Diesease. M. U. Gillette. **119:** 83-103.	1	0	0	0	0	
Buijs, R. M., C. Escobar and D. F. Swaab (2013). "The circadian system and the balance of the autonomic nervous system." Handbook of clinical neurology **117**: 173-191.	1	0	0	0	0	
Buijs, R. M., M. A. Guzman Ruiz, R. Mendez Hernandez and B. Rodriguez Cortes (2019). "The suprachiasmatic nucleus; a responsive clock regulating homeostasis by daily changing the setpoints of physiological parameters." Autonomic Neuroscience-Basic & Clinical **218**: 43-50.	1	0	0	0	0	
Cai, C., B. Vandermeer, R. Khurana, K. Nerenberg, R. Featherstone, M. Sebastianski and M. H. Davenport (2019). "The impact of occupational shift work and working hours during pregnancy on health outcomes: a systematic review and meta-analysis." American Journal of Obstetrics and Gynecology **221**(6): 563-576.	1	1	0	0	0	
Cai, C., B. Vandermeer, R. Khurana, K. Nerenberg, R. Featherstone, M. Sebastianski and M. H. Davenport (2020). "The impact of occupational activities during pregnancy on pregnancy outcomes: a systematic review and metaanalysis." American Journal of Obstetrics and Gynecology **222**(3): 224-238.	1	0	0	0	0	
Cappuccio, F. P. and M. A. Miller (2017). "Sleep and Cardio-Metabolic Disease." Current Cardiology Reports **19**(11).	1	1	0	0	0	
Chan, O. Y. (1994). "Health of female shiftworkers in Singapore." Annals of the Academy of Medicine, Singapore **23**(5): 706-709.	1	1	0	0	0	

Chellappa, S. L., N. Vujovic, J. S. Williams and F. A. J. L. Scheer (2019). "Impact of Circadian Disruption on Cardiovascular Function and Disease." Trends in Endocrinology and Metabolism **30**(10): 767-779.	1	1	0	0	0	
Cheungpasitporn, W., C. Thongprayoon, N. Srivali, P. Vijayvargiya, C. A. Andersen, W. Kittanamongkolchai, I. J. J. Sathick, S. M. Caples and S. B. Erickson (2016). "The effects of napping on the risk of hypertension: a systematic review and meta-analysis." Journal of evidence-based medicine **9**(4): 205-212.	1	0	0	0	0	
Clougherty, J. E., K. Souza and M. R. Cullen (2010). Work and its role in shaping the social gradient in health. Biology of Disadvantage: Socioeconomic Status and Health. N. E. Adler and J. Stewart. **1186:** 102-124.	1	0	0	0	0	
Cornelissen, G., J. Halberg, F. Halberg, S. Sanchez de la Pena, W. Nelson, O. Schwartzkopff, A. Stoynev and E. Haus (2008). "Schedule shifts, cancer and longevity: good, bad or indifferent?" Journal of experimental therapeutics & oncology **7**(4): 263-273.	1	0	0	0	0	
Crnko, S., B. C. Du Pre, J. P. G. Sluijter and L. W. Van Laake (2019). "Circadian rhythms and the molecular clock in cardiovascular biology and disease." Nature Reviews Cardiology **16**(7): 437-447.	1	1	0	0	0	
Culpepper, L. (2010). "The social and economic burden of shift-work disorder." Journal of Family Practice **59**(1): S3-S11.	1	0	0	0	0	
da Silva, F. R., et al. (2020). "Does the compromised sleep and circadian disruption of night and shiftworkers make them highly vulnerable to 2019 coronavirus disease (COVID-19)?" Chronobiology International 37(5): 607-617.	1	0	0	0	0	
Deng, N., T. P. Kohn, L. I. Lipshultz and A. W. Pastuszak (2018). "The Relationship Between Shift Work and Men's Health." Sexual Medicine Reviews **6**(3): 446-456.	1	1	1	1	0	NARRATIVE
Diene, E., A. Fouquet and Y. Esquirol (2012). "Cardiovascular diseases and psychosocial factors at work." Archives of Cardiovascular Diseases **105**(1): 33-39.	1	1	1	1	0	NO EVALUATION OF THE ASSOCIATION BETWEEN SW AND OUTCOME
Duez, H. and B. Staels (2010). "Nuclear Receptors Linking Circadian Rhythms and Cardiometabolic Control." Arteriosclerosis Thrombosis and Vascular Biology **30**(8): 1529-1534.	1	0	0	0	0	

Egan, K. J., K. L. Knutson, A. C. Pereira and M. von Schantz (2017). "The role of race and ethnicity in sleep, circadian rhythms and cardiovascular health." Sleep Medicine Reviews **33**: 70-78.	1	0	0	0	0	
Egstrand, S., et al. (2021). "The Vascular Circadian Clock in Chronic Kidney Disease." Cells 10(7).	1	0	0	0	0	
Ekmekcioglu, C. (2014). "Expression and putative functions of melatonin receptors in malignant cells and tissues." Wiener medizinische Wochenschrift (1946) **164**(21-22): 472-478.	1	0	0	0	0	
Fink, A. M. (2020). "MEASURING THE EFFECTS OF NIGHT-SHIFT WORK ON CARDIAC AUTONOMIC MODULATION: AN APPRAISAL OF HEART RATE VARIABILITY METRICS." International Journal of Occupational Medicine and Environmental Health 33(4): 409-425.	1	0	0	0	0	
Firsov, D. and O. Bonny (2010). "Circadian regulation of renal function." Kidney International **78**(7): 640-645.	1	0	0	0	0	
Frost, P., H. A. Kolstad and J. P. Bonde (2009). "Shift work and the risk of ischemic heart disease - a systematic review of the epidemiologic evidence." Scandinavian Journal of Work Environment & Health **35**(3): 163-179.	1	0	0	0	0	
Gamaldo, C. E., Y. Chung, Y. M. Kang and R. M. E. Salas (2014). "Tick-tock-tick-tock: the impact of circadian rhythm disorders on cardiovascular health and wellness." Journal of the American Society of Hypertension **8**(12): 921-929.	1	1	0	0	0	
Gamble, K. L. and M. E. Young (2013). "Metabolism as an integral cog in the mammalian circadian clockwork." Critical Reviews in Biochemistry and Molecular Biology **48**(4): 317-331.	1	0	0	0	0	
Gangwisch, J. E. (2009). "Epidemiological evidence for the links between sleep, circadian rhythms and metabolism." Obesity Reviews **10**: 37-45.	1	0	0	0	0	
Gerhart-Hines, Z. and M. A. Lazar (2015). "Circadian Metabolism in the Light of Evolution." Endocrine Reviews **36**(3): 289-304.	1	0	0	0	0	
Gold, E. B. and E. Tomich (1994). "OCCUPATIONAL HAZARDS TO FERTILITY AND PREGNANCY OUTCOME." Occupational Medicine-State of the Art Reviews **9**(3): 435-469.	1	0	0	0	0	

Halberg, F., G. Cornelissen, Y. Kumagai, C. Bingham, J. Saito, K. Tamura, K. Otsuka, T. Breus, S. Rapoport, F. Komarov, R. Zaslavskaya, F. Delpozo, M. J. Rodriguez, M. T. Arredondo, E. Gomez, A. Portela, A. Ebihara, E. Haus, F. Halberg, J. Halberg, S. Fujii, P. Delmore and E. Bakken (1994). "TELEHYGIENE SYSTEM FOR PREVENTIVE CHRONOPHARMACOLOGY IN-SPACE AND REMOTE AREAS ON EARTH." Chronobiologia **21**(1-2): 33-43.	1	0	0	0	0	
Hirose, T. (2005). "An occupational health physician's report on the improvement in the sleeping conditions of night shift workers." Industrial Health **43**(1): 58-62.	1	0	0	0	0	
Hsu, C.-N. and Y.-L. Tain (2020). "Light and Circadian Signaling Pathway in Pregnancy: Programming of Adult Health and Disease." International Journal of Molecular Sciences **21**(6).	1	0	0	0	0	
Hwang, W. J. and O. Hong (2012). "Work-related cardiovascular disease risk factors using a socioecological approach: implications for practice and research." European Journal of Cardiovascular Nursing **11**(1): 114-126.	1	1	0	0	0	
Joyce, K., R. Pabayo, J. A. Critchley and C. Bambra (2010). "Flexible working conditions and their effects on employee health and wellbeing." Cochrane Database of Systematic Reviews(2).	1	1	0	0	0	
Kales, S. N., A. J. Tsismenakis, C. Zhang and E. S. Soteriades (2009). "Blood-Pressure in Firefighters, Police Officers, and Other Emergency Responders." American Journal of Hypertension **22**(1): 11-20.	1	0	0	0	0	
Ke, D.-S. (2012). "Overwork, stroke, and karoshi-death from overwork." Acta neurologica Taiwanica **21**(2): 54-59.	1	0	0	0	0	
Kervezee, L., A. Kosmadopoulos and D. B. Boivin (2020). "Metabolic and cardiovascular consequences of shift work: The role of circadian disruption and sleep disturbances." European Journal of Neuroscience **51**(1): 396-412.	1	1	0	0	0	
Khaper, N., C. D. C. Bailey, N. R. Ghugre, C. Reitz, Z. Awosanmi, R. Waines and T. A. Martino (2018). "Implications of disturbances in circadian rhythms for cardiovascular health: A new frontier in free radical biology." Free Radical Biology and Medicine **119**: 85-92.	1	0	0	0	0	
Klerman, E. B. (2005). "Clinical aspects of human circadian rhythms." Journal of Biological Rhythms **20**(4): 375-386.	1	0	0	0	0	

Knutsson, A. and H. Boggild (2000). "Shiftwork and cardiovascular disease: review of disease mechanisms." Reviews on environmental health **15**(4): 359-372.	1	1	0	0	0	
Koo, D. L., H. Nam, R. J. Thomas and C.-H. Yun (2018). "Sleep Disturbances as a Risk Factor for Stroke." Journal of Stroke **20**(1): 12-32.	1	0	0	0	0	
Kosir, R., K. Spaninger and D. Rozman (2013). "Circadian Events in Human Diseases and in Cytochrome P450-Related Drug Metabolism and Therapy." Iubmb Life **65**(6): 487-496.	1	0	0	0	0	
Kozlowska, L., J. Gromadzinska and W. Wasowicz (2019). "HEALTH RISK IN TRANSPORT WORKERS PART II. DIETARY COMPOUNDS AS MODULATORS OF OCCUPATIONAL EXPOSURE TO CHEMICALS." International Journal of Occupational Medicine and Environmental Health **32**(4): 441-464.	1	1	1	1	0	NO EVALUATION OF THE ASSOCIATION BETWEEN SW AND OUTCOME
Kristensen, T. S. (1989). "Cardiovascular diseases and the work environment. A critical review of the epidemiologic literature on nonchemical factors." Scandinavian journal of work, environment & health **15**(3): 165-179.	1	1	1	1	0	NO EVALUATION OF THE ASSOCIATION BETWEEN SW AND OUTCOME
Kumar, P. V. A., P. P. Dakup, S. Sarkar, J. B. Modasia, M. S. Motzner and S. Gaddameedhi (2019). "It's About Time: Advances in Understanding the Circadian Regulation of DNA Damage and Repair in Carcinogenesis and Cancer Treatment Outcomes." Yale Journal of Biology and Medicine **92**(2): 305-316.	1	0	0	0	0	
Kumar, R. (2008). "Approved and investigational uses of modafinil - An evidence-based review." Drugs **68**(13): 1803-1839.	1	0	0	0	0	
Leger, D., Y. Esquirol, C. Gronfier, A. Metlaine and Sfrms (2018). "Shift-workers and night-workers' health consequences: State of art and recommendations." Presse Medicale **47**(11-12): 991-999.	1	1	1	1	0	NARRATIVE
Liira, J., J. H. Verbeek, G. Costa, T. R. Driscoll, M. Sallinen, L. K. Isotalo and J. H. Ruotsalainen (2014). "Pharmacological interventions for sleepiness and sleep disturbances caused by shift work." Cochrane Database of Systematic Reviews(8).	1	0	0	0	0	
Liu, J. A., et al. (2021). "Disruptions of Circadian Rhythms and Thrombolytic Therapy During Ischemic Stroke Intervention." Frontiers in Neuroscience 15.	1	0	0	0	0	
Liu, P. Y. (2019). "A Clinical Perspective of Sleep and Andrological Health: Assessment, Treatment Considerations, and Future Research." Journal of Clinical Endocrinology & Metabolism **104**(10): 4398-4417.	1	0	0	0	0	

Liu, Q., J. Shi, P. Duan, B. Liu, T. Li, C. Wang, H. Li, T. Yang, Y. Gan, X. Wang, S. Cao and Z. Lu (2018). "Is shift work associated with a higher risk of overweight or obesity? A systematic review of observational studies with meta-analysis." International Journal of Epidemiology **47**(6): 1956-1971.	1	0	0	0	0	
Makarem, N., et al. (2021). "Effect of Sleep Disturbances on Blood Pressure." Hypertension 77(4): 1036-1046.	1	0	0	0	0	
Mansukhani, M. P., N. Covassin and V. K. Somers (2019). "Apneic Sleep, Insufficient Sleep, and Hypertension." Hypertension **73**(4): 744-756.	1	0	0	0	0	
Martino, T. A. and M. E. Young (2015). "Influence of the Cardiomyocyte Circadian Clock on Cardiac Physiology and Pathophysiology." Journal of Biological Rhythms **30**(3): 183-205.	1	0	0	0	0	
McDermott, M., D. L. Brown and R. D. Chervin (2018). "Sleep disorders and the risk of stroke." Expert Review of Neurotherapeutics **18**(7): 523-531.	1	0	0	0	0	
Melendez-Fernandez, O. H., et al. (2021). "Clocks, Rhythms, Sex, and Hearts: How Disrupted Circadian Rhythms, Time-of-Day, and Sex Influence Cardiovascular Health." Biomolecules 11(6).	1	0	0	0	0	
Mongrain, V. and N. Cermakian (2009). "Clock genes in health and diseases." Journal of Applied Biomedicine **7**(1): 15-33.	1	0	0	0	0	
Morris, C. J., D. Aeschbach and F. A. J. L. Scheer (2012). "Circadian system, sleep and endocrinology." Molecular and Cellular Endocrinology **349**(1): 91-104.	1	0	0	0	0	
Morris, C. J., J. N. Yang and F. A. J. L. Scheer (2012). The impact of the circadian timing system on cardiovascular and metabolic function. Neurobiology of Circadian Timing. A. Kalsbeek, M. Merrow, T. Roenneberg and R. G. Foster. **199:** 337-358.	1	0	0	0	0	
Mosendane, T., T. Mosendane and F. J. Raal (2008). "Shift work and its effects on the cardiovascular system." Cardiovascular Journal of Africa **19**(4): 210-215.	1	1	0	0	0	
Mozurkewich, E. L., B. Luke, M. Avni and F. M. Wolf (2000). "Working conditions and adverse pregnancy outcome: A meta-analysis." Obstetrics and Gynecology **95**(4): 623-635.	1	0	0	0	0	
Munakata, M. (2018). "Clinical significance of stress-related increase in blood pressure: current evidence in office and out-of-office settings." Hypertension Research **41**(8): 553-569.	1	0	0	0	0	

Neumann, A.-M., C. X. Schmidt, R. M. Brockmann and H. Oster (2019). "Circadian regulation of endocrine systems." Autonomic Neuroscience-Basic & Clinical **216**: 1-8.	1	0	0	0	0	
Nicholson, P. J. and D. A. P. D'Auria (1999). "Shift work, health, the working time regulations and health assessments." Occupational Medicine-Oxford **49**(3): 127-137.	1	1	0	0	0	
Okamura, H., M. Doi, K. Goto and R. Kojima (2016). "Clock genes and salt-sensitive hypertension: a new type of aldosterone-synthesizing enzyme controlled by the circadian clock and angiotensin II." Hypertension Research **39**(10): 681-687.	1	0	0	0	0	
Okamura, H., M. Doi, Y. Yamaguchi and J.-M. Fustin (2011). "Hypertension Due to Loss of Clock: Novel Insight From the Molecular Analysis of Cry1/Cry2-Deleted Mice." Current Hypertension Reports **13**(2): 103-108.	1	0	0	0	0	
Olaoye, O. A., S. H. Masten, R. Mohandas and M. L. Gumz (2019). "Circadian Clock Genes in Diabetic Kidney Disease (DKD)." Current Diabetes Reports **19**(7).	1	0	0	0	0	
Oyiengo, D., M. Louis, B. Hott and G. Bourjeily (2014). "Sleep Disorders in Pregnancy." Clinics in Chest Medicine **35**(3): 571-+.	1	0	0	0	0	
Palmer, K. T., M. Bonzini and J.-P. E. Bonde (2013). "Pregnancy: occupational aspects of management: concise guidance." Clinical Medicine **13**(1): 75-79.	1	0	0	0	0	
Palmer, K. T., M. Bonzini, E. C. Harris, C. Linaker and J. P. Bonde (2013). "Work activities and risk of prematurity, low birth weight and pre-eclampsia: an updated review with meta-analysis." Occupational and Environmental Medicine **70**(4): 213-222.	1	0	0	0	0	
Pandalai, S. P., P. A. Schulte and D. B. Miller (2013). "Conceptual heuristic models of the interrelationships between obesity and the occupational environment." Scandinavian Journal of Work Environment & Health **39**(3): 221-232.	1	0	0	0	0	
Paschos, G. K., J. E. Baggs, J. B. Hogenesch and G. A. FitzGerald (2010). "The Role of Clock Genes in Pharmacology." Annual Review of Pharmacology and Toxicology **50**: 187-214.	1	0	0	0	0	
Patterson, P. D., et al. (2020). "Impact of shift work on blood pressure among emergency medical services clinicians and related shift workers: A systematic review and meta-analysis." Sleep Health 6(3): 387-398.	1	1	1	1	0	NO EVALUATION OF THE ASSOCIATION BETWEEN SW AND OUTCOME

Peter, R. and J. Siegrist (2000). "Psychosocial work environment and the risk of coronary heart disease." International Archives of Occupational and Environmental Health **73**: S41-S45.	1	1	0	0	0	
Pickering, T. G. (2006). "Could hypertension be a consequence of the 24/7 society? The effects of sleep deprivation and shift work." Journal of clinical hypertension (Greenwich, Conn.) **8**(11): 819-822.	1	1	1	1	0	NARRATIVE
Portaluppi, F., P. Cortelli, G. C. Buonaura, M. H. Smolensky and F. Fabbian (2009). "Do Restless Legs Syndrome (RLS) and Periodic Limb Movements of Sleep (PLMS) Play a Role in Nocturnal Hypertension and Increased Cardiovascular Risk of Renally Impaired Patients?" Chronobiology International **26**(6): 1206-1221.	1	0	0	0	0	
Pot, G. K., S. Almoosawi and A. M. Stephen (2016). "Meal irregularity and cardiometabolic consequences: results from observational and intervention studies." Proceedings of the Nutrition Society **75**(4): 475-486.	1	0	0	0	0	
Prasai, M. J., J. T. George and E. M. Scott (2008). "Molecular clocks, type 2 diabetes and cardiovascular disease." Diabetes & Vascular Disease Research **5**(2): 89-95.	1	0	0	0	0	
Puttonen, S., M. Harma and C. Hublin (2010). "Shift work and cardiovascular disease - pathways from circadian stress to morbidity." Scandinavian Journal of Work Environment & Health **36**(2): 96-108.	1	1	0	0	0	
Reitz, C. J. and T. A. Martino (2015). "Disruption of Circadian Rhythms and Sleep on Critical Illness and the Impact on Cardiovascular Events." Current Pharmaceutical Design **21**(24): 3505-3511.	1	0	0	0	0	
Richards, J. and M. L. Gumz (2012). "Advances in understanding the peripheral circadian clocks." Faseb Journal **26**(9): 3602-3613.	1	0	0	0	0	
Sakamoto, Y. S., F. Porto-Sousa and C. Salles (2018). "Prevalence of obstructive sleep apnea in shift workers: a systematic review." Ciencia & Saude Coletiva **23**(10): 3381-3392.	1	0	0	0	0	
Sakuraya, A., K. Watanabe, N. Kawakami, K. Imamura, E. Ando, Y. Asai, H. Eguchi, Y. Kobayashi, N. Nishida, H. Arima, A. Shimazu and A. Tsutsumi (2017). "Work-related psychosocial factors and onset of metabolic syndrome among workers: a systematic review and meta-analysis protocol." Bmj Open **7**(6).	1	1	0	0	0	
Sanchez-Barcelo, E. J., M. D. Mediavilla, D. X. Tan and R. J. Reiter (2010). "Clinical Uses of Melatonin: Evaluation of Human Trials." Current Medicinal Chemistry **17**(19): 2070-2095.	1	0	0	0	0	

Shaw, E. and G. H. Tofler (2009). "Circadian rhythm and cardiovascular disease." Current Atherosclerosis Reports **11**(4): 289-295.	1	0	0	0	0	
Simko, F., R. J. Reiter, O. Pechanova and L. Paulis (2013). "Experimental models of melatonin-deficient hypertension." Frontiers in Bioscience-Landmark **18**: 616-625.	1	0	0	0	0	
Skogstad, M., H. A. Johannessen, T. Tynes, I. S. Mehlum, K. C. Nordby and A. Lie (2016). "Systematic review of the cardiovascular effects of occupational noise." Occupational Medicine-Oxford **66**(1): 10-16.	1	0	0	0	0	
Slanovic-Kuzmanovic, Z., I. Kos and A.-M. Domijan (2013). "ENDOCRINE, LIFESTYLE, AND GENETIC FACTORS IN THE DEVELOPMENT OF METABOLIC SYNDROME." Arhiv Za Higijenu Rada I Toksikologiju-Archives of Industrial Hygiene and Toxicology **64**(4): 581-591.	1	0	0	0	0	
Smolensky, M. H., R. C. Hermida, A. Reinberg, L. Sackett-Lundeen and F. Portaluppi (2016). "Circadian disruption: New clinical perspective of disease pathology and basis for chronotherapeutic intervention." Chronobiology International **33**(8): 1101-1119.	1	0	0	0	0	
Soteriades, E. S., D. L. Smith, A. J. Tsismenakis, D. M. Baur and S. N. Kales (2011). "Cardiovascular Disease in US Firefighters A Systematic Review." Cardiology in Review **19**(4): 202-215.	1	0	0	0	0	
Steenland, K., L. Fine, K. Belkic, P. Landsbergis, P. Schnall, D. Baker, T. Theorell, J. Siegrist, R. Peter, R. Karasek, M. Marmot, C. Brisson and F. Tuchsen (2000). "Research findings linking workplace factors to cardiovascular disease outcomes." Occupational Medicine-State of the Art Reviews **15**(1): 7-68.	1	1	1	1	0	NARRATIVE
Strohmaier, S., E. E. Devore, Y. Zhang and E. S. Schernhammer (2018). "A Review of Data of Findings on Night Shift Work and the Development of DM and CVD Events: a Synthesis of the Proposed Molecular Mechanisms." Current Diabetes Reports **18**(12).	1	0	0	0	0	
Thomas, A., A. Chan, M. B. Hunks and J. Zheng (2007). "Providing an in-centre nocturnal hemodialysis program: the pearls and pitfalls." CANNT journal = Journal ACITN **17**(4): 39-41.	1	0	0	0	0	
Thosar, S. S., M. P. Butler and S. A. Shea (2018). "Role of the circadian system in cardiovascular disease." Journal of Clinical Investigation **128**(6): 2157-2167.	1	0	0	0	0	

Togo, F. and M. Takahashi (2009). "Heart Rate Variability in Occupational Health -A Systematic Review." Industrial Health **47**(6): 589-602.	1	1	0	0	0	
Van Ryswyk, E., S. Mukherjee, C. L. Chai-Coetzer, A. Vakulin and R. D. McEvoy (2018). "Sleep Disorders, Including Sleep Apnea and Hypertension." American Journal of Hypertension **31**(8): 857-864.	1	0	0	0	0	
Wallace, D. M., A. R. Ramos and T. Rundek (2012). "Sleep disorders and stroke." International Journal of Stroke **7**(3): 231-242.	1	0	0	0	0	
Watanabe, K., A. Sakuraya, N. Kawakami, K. Imamura, E. Ando, Y. Asai, H. Eguchi, Y. Kobayashi, N. Nishida, H. Arima, A. Shimazu and A. Tsutsumi (2018). "Work-related psychosocial factors and metabolic syndrome onset among workers: a systematic review and meta-analysis." Obesity Reviews **19**(11): 1557-1568.	1	1	0	0	0	
Wolk, R., A. S. Gami, A. Garcia-Touchard and V. K. Somers (2005). "Sleep and cardiovascular disease." Current Problems in Cardiology **30**(12): 625-662.	1	0	0	0	0	
Wolk, R. and V. K. Somers (2007). "Sleep and the metabolic syndrome." Experimental Physiology **92**(1): 67-78.	1	0	0	0	0	
Xu, W., et al. (2021). "Molecular link between circadian clocks and cardiac function: a network of core clock, slave clock, and effectors." Current Opinion in Pharmacology 57: 28-40.	1	0	0	0	0	
Young, M. E. (2006). "The circadian clock within the heart: potential influence on myocardial gene expression, metabolism, and function." American Journal of Physiology-Heart and Circulatory Physiology **290**(1): H1-H16.	1	0	0	0	0	
Zimmerman, F. H. (2012). "Cardiovascular Disease and Risk Factors in Law Enforcement Personnel: A Comprehensive Review." Cardiology in Review **20**(4): 159-166.	1	1	0	0	0	
Zisapel, N. (2018). "New perspectives on the role of melatonin in human sleep, circadian rhythms and their regulation." British Journal of Pharmacology **175**(16): 3190-3199.	1	1	0	0	0	

SMOKING HABITS	IDENTIFICATION	SCREENING		ELIGIBILITY	INCLUDED	REASON FOR EXCLUSION
	SEARCH / REFERENCE LIST	TITLE	ABSTRACT	FULL TEXT	FULL TEST	
Adams, S. T., et al. (2013). "Roux-en-Y gastric bypass for morbid obesity: what are the preoperative predictors of weight loss?" Postgraduate Medical Journal 89(1053): 411-416.	1	0	0	0	0	
Alfredsson, L. and T. Olsson (2019). "Lifestyle and Environmental Factors in Multiple Sclerosis." Cold Spring Harbor Perspectives in Medicine 9(4).	1	0	0	0	0	
Amiri, S. (2021). "Meta inference of heterogeneous data streams." Journal of Biopharmaceutical Statistics.	1	0	0	0	0	
Bonnefond, A., et al. (2004). "A critical review of techniques aiming at enhancing and sustaining worker's alertness during the night shift." Industrial Health 42(1): 1-14.	1	0	0	0	0	
Canuto, R., et al. (2013). "Metabolic syndrome and shift work: A systematic review." Sleep Medicine Reviews 17(6): 425-431.	1	1	1	1	0	NO EVALUATION OF THE ASSOCIATION BETWEEN SW AND OUTCOME
Cole, R. J., et al. (1990). "PSYCHIATRIC ASPECTS OF SHIFTWORK." Occupational Medicine-State of the Art Reviews 5(2): 301-314.	1	0	0	0	0	
Daly, A. A., et al. (2021). "A Review of Modifiable Risk Factors in Young Women for the Prevention of Breast Cancer." Breast Cancer-Targets and Therapy 13: 241-257.	1	0	0	0	0	
Engel, C. L., et al. (2018). "Work and Female Breast Cancer: The State of the Evidence, 2002-2017." New Solutions-a Journal of Environmental and Occupational Health Policy 28(1): 55-78.	1	0	0	0	0	
Foster, R. G. and K. Wulff (2005). "The rhythm of rest and excess." Nature Reviews Neuroscience 6(5): 407-414.	1	0	0	0	0	
Frost P, Kolstad HA, Bonde JP. Shift work and the risk of ischemic heart disease – a systematic review of the epidemiologic evidence. Scand J Work Environ Health. 2009;35(3):163–79.	1	1	1	1	0	NO EVALUATION OF THE ASSOCIATION BETWEEN SW AND OUTCOME
Gestal, J. J. (1987). "OCCUPATIONAL HAZARDS IN HOSPITALS - ACCIDENTS, RADIATION, EXPOSURE TO NOXIOUS CHEMICALS, DRUG-	1	0	0	0	0	

ADDICTION AND PSYCHIC PROBLEMS, AND ASSAULT." British Journal of Industrial Medicine 44(8): 510-520.						
Golmohammadi, R. and E. Darvishi (2019). "The combined effects of occupational exposure to noise and other risk factors - a systematic review." Noise & Health 21(101): 125-141.	1	0	0	0	0	
Guandalini, L. S., et al. (2020). "Analysis of the evidence of related factors, associated conditions and at-risk populations of the NANDA-I nursing diagnosis insomnia." International Journal of Nursing Sciences 7(4): 376-386	1	0	0	0	0	
Hachim, M. Y., et al. (2019). "The Beneficial and Debilitating Effects of Environmental and Microbial Toxins, Drugs, Organic Solvents and Heavy Metals on the Onset and Progression of Multiple Sclerosis." Toxins 11(3).	1	0	0	0	0	
Hedstrom, A. K., et al. (2016). "Environmental factors and their interactions with risk genotypes in MS susceptibility." Current Opinion in Neurology 29(3): 293-298.	1	0	0	0	0	
Hedstrom, A. K., et al. (2015). "The Role of Environment and Lifestyle in Determining the Risk of Multiple Sclerosis." Current topics in behavioral neurosciences 26: 87-104.	1	0	0	0	0	
Hwang, W. J. and O. Hong (2012). "Work-related cardiovascular disease risk factors using a socioecological approach: implications for practice and research." European Journal of Cardiovascular Nursing 11(1): 114-126.	1	1	1	1	0	NO EVALUATION OF THE ASSOCIATION BETWEEN SW AND OUTCOME
Johnson, J. V. and J. Lipscomb (2006). "Long working hours, occupational health and the changing nature of work organization." American Journal of Industrial Medicine 49(11): 921-929.	1	1	0	0	0	
Knutson, A. and H. Boggild (2010). "Gastrointestinal disorders among shift workers." Scandinavian Journal of Work Environment & Health 36(2): 85-95.	1	0	0	0	0	
Knutsson, A. (1989). "Shift work and coronary heart disease." Scandinavian journal of social medicine. Supplementum 44: 1-36.	1	1	0	0	0	
Knutsson, A. and H. Boggild (2000). "Shiftwork and cardiovascular disease: review of disease mechanisms." Reviews on environmental health 15(4): 359-372.	1	1	1	1	0	NO EVALUATION OF THE ASSOCIATION BETWEEN SW AND OUTCOME

Kristensen, T. S. (1989). "Work environment and cardiovascular diseases. A short review of the literature." Journal of UOEH 11 Suppl: 120-133.	1	1	0	0	0	
Kumar, S., et al. (2019). "Environmental & occupational exposure & female reproductive dysfunction." Indian Journal of Medical Research 150(6): 532-545.	1	0	0	0	0	
Kwiatkowski, F., et al. (2005). "Stress, cancer and circadian rhythm of melatonin." Pathologie Biologie 53(5): 269-272.	1	0	0	0	0	
Legg, S. J. (1987). "PHYSIOLOGICAL ERGONOMICS IN NURSING." International Journal of Nursing Studies 24(4): 299-305.	1	0	0	0	0	
Li, W., et al. (2021). "Association of noise exposure with risk of metabolic syndrome: Evidence from 44,698 individuals." Diabetes research and clinical practice 178: 108944-108944	1	0	0	0	0	
Mendoza, J. (2019). "Food intake and addictive-like eating behaviors: Time to think about the circadian clock(s)." Neuroscience and Biobehavioral Reviews 106: 122-132.	1	0	0	0	0	
Murillo-Rodriguez, E., et al. (2018). "An Overview of the Clinical Uses, Pharmacology, and Safety of Modafinil." Acs Chemical Neuroscience 9(2): 151-158.	1	0	0	0	0	
Nakata, A., et al. (2010). "Nurses, Smoking, and Immunity: A Review." Rehabilitation Nursing 35(5): 198-205.	1	1	1	1	0	NARRATIVE
Nea, F. M., et al. (2015). "Dietary and lifestyle habits and the associated health risks in shift workers." Nutrition Research Reviews 28(2): 143-166.	1	1	0	0	0	
Nicholson, P. J. and D. A. P. D'Auria (1999). "Shift work, health, the working time regulations and health assessments." Occupational Medicine-Oxford 49(3): 127-137.	1	1	0	0	0	
Nurminen, T. (1998). "Shift work and reproductive health." Scandinavian Journal of Work Environment & Health 24: 28-34.	1	0	0	0	0	
Olsson, T., et al. (2017). "Interactions between genetic, lifestyle and environmental risk factors for multiple sclerosis." Nature Reviews Neurology 13(1): 25-36.	1	0	0	0	0	
Peplonska, B., et al. (2019). "THE ASSOCIATION BETWEEN NIGHT SHIFT WORK AND NUTRITION PATTERNS AMONG NURSES: A LITERATURE REVIEW." Medycyna Pracy 70(3): 363-376.	1	0	0	0	0	
Pritchett, D. and A. B. Reddy (2015). "Circadian Clocks in the Hematologic System." Journal of Biological Rhythms 30(5): 374-388.	1	0	0	0	0	

Pukkala, E., et al. (2009). "Occupation and cancer - follow-up of 15 million people in five Nordic countries." Acta Oncologica 48(5): 646-790.	1	0	0	0	0	
Puttonen, S., et al. (2010). "Shift work and cardiovascular disease - pathways from circadian stress to morbidity." Scandinavian Journal of Work Environment & Health 36(2): 96-108.	1	1	1	1	0	NARRATIVE
Radowicka, M., et al. (2013). "Assessment of the occurrence of menstrual disorders in female flight attendants - preliminary report and literature review." Neuroendocrinology Letters 34(8): 809-813.	1	0	0	0	0	
Ryden, L. A., et al. (1989). "OCCUPATIONAL LOW-BACK INJURY IN A HOSPITAL EMPLOYEE POPULATION - AN EPIDEMIOLOGIC ANALYSIS OF MULTIPLE RISK-FACTORS OF A HIGH-RISK OCCUPATIONAL GROUP." Spine 14(3): 315-320.	1	0	0	0	0	
Scott, A. J. and J. Ladou (1990). "SHIFTWORK - EFFECTS ON SLEEP AND HEALTH WITH RECOMMENDATIONS FOR MEDICAL SURVEILLANCE AND SCREENING." Occupational Medicine-State of the Art Reviews 5(2): 273-299.	1	1	0	0	0	
Shephard, R. J. (2017). "Peptic Ulcer and Exercise." Sports Medicine 47(1): 33-40.	1	0	0	0	0	
Shochat, T. (2012). "Impact of lifestyle and technology developments on sleep." Nature and science of sleep 4: 19-31.	1	0	0	0	0	
Smolensky, M. H., et al. (2016). "Circadian disruption: New clinical perspective of disease pathology and basis for chronotherapeutic intervention." Chronobiology International 33(8): 1101-1119.	1	0	0	0	0	
Snedeker, S. M. (2006). "Chemical exposures in the workplace: effect on breast cancer risk among women." AAOHN journal : official journal of the American Association of Occupational Health Nurses 54(6): 270-279; quiz 280-271.	1	0	0	0	0	
Soteriades, E. S., et al. (2011). "Cardiovascular Disease in US Firefighters A Systematic Review." Cardiology in Review 19(4): 202-215.	1	1	0	0	0	
Spurgeon, A., et al. (1997). "Health and safety problems associated with long working hours: A review of the current position." Occupational and Environmental Medicine 54(6): 367-375.	1	1	0	0	0	
Steenland, K. (1996). "Epidemiology of occupation and coronary heart disease: Research agenda." American Journal of Industrial Medicine 30(4): 495-499.	1	0	0	0	0	

Sun, X., et al. (2020). "The association between sleep chronotype and obesity among black and white participants of the Bogalusa Heart Study." Chronobiology International 37(1): 123-134.	1	0	0	0	0	
Tanner, C. M. (2010). "Advances in Environmental Epidemiology." Movement Disorders 25(3): S58-S62.	1	0	0	0	0	
Tirabassi, G., et al. (2014). "Harmful effects of functional hypercortisolism: a working hypothesis." Endocrine 46(3): 370-386.	1	0	0	0	0	
Toren, K., et al. (1996). "Health effects of working in pulp and paper mills: Exposure, obstructive airways diseases, hypersensitivity reactions, and cardiovascular diseases." American Journal of Industrial Medicine 29(2): 111-122.	1	0	0	0	0	
Virtanen, M., et al. (2015). "Long working hours and alcohol use: systematic review and meta-analysis of published studies and unpublished individual participant data." Bmj-British Medical Journal 350.	1	0	0	0	0	
Vyas, M. V., et al. (2012). "Shift work and vascular events: systematic review and meta-analysis." Bmj-British Medical Journal 345.	1	1	0	0	0	
Weiderpass, E., et al. (2011). "Risk factors for breast cancer, including occupational exposures." Safety and health at work 2(1): 1-8.	1	0	0	0	0	
Wittert, G. (2014). "The relationship between sleep disorders and testosterone in men." Asian Journal of Andrology 16(2): 262-265.	1	0	0	0	0	
Wosu, A. C., et al. (2013). "Correlates of cortisol in human hair: implications for epidemiologic studies on health effects of chronic stress." Annals of Epidemiology 23(12): 797-811.	1	0	0	0	0	
Yu, E., et al. (2016). "Diet, Lifestyle, Biomarkers, Genetic Factors, and Risk of Cardiovascular Disease in the Nurses' Health Studies." American Journal of Public Health 106(9): 1616-1623.	1	1	0	0	0	
Zimmerman, F. H. (2012). "Cardiovascular Disease and Risk Factors in Law Enforcement Personnel: A Comprehensive Review." Cardiology in Review 20(4): 159-166.	1	1	0	0	0	

SEDENTARINESS	IDENTIFICATION	SCREENING		ELIGIBILITY	INCLUDED	REASON FOR EXCLUSION
	SEARCH / REFERENCE LIST	TITLE	ABSTRACT	FULL TEXT	FULL TEST	
Adams, S. T., et al. (2013). "Roux-en-Y gastric bypass for morbid obesity: what are the preoperative predictors of weight loss?" Postgraduate Medical Journal 89(1053): 411-416.	1	0	0	0	0	
Aisbett, B., et al. (2017). "The Impact of Shiftwork on Skeletal Muscle Health." Nutrients 9(3).	1	0	0	0	0	
Allaf, M., et al. (2021). "Intermittent fasting for the prevention of cardiovascular disease." Cochrane Database of Systematic Reviews(1).	1	0	0	0	0	
Ancoli-Israel, S., et al. (2003). "The role of actigraphy in the study of sleep and circadian rhythms." Sleep 26(3): 342-392.	1	0	0	0	0	
Anothaisintawee, T., et al. (2016). "Sleep disturbances compared to traditional risk factors for diabetes development: Systematic review and meta-analysis." Sleep Medicine Reviews 30: 11-24.	1	0	0	0	0	
Aoyama, S., et al. (2021). "Chrono-Nutrition Has Potential in Preventing Age-Related Muscle Loss and Dysfunction." Frontiers in Neuroscience 15.	1	0	0	0	0	
Apostolopoulos, Y., et al. (2010). "Worksite-Induced Morbidities Among Truck Drivers in the United States." Aaohn Journal 58(7): 285-296.	1	1	0	0	0	
Arveux, P. and A. Bertaut (2013). "Epidemiology of breast cancer." La Revue du praticien 63(10): 1362-1366.	1	0	0	0	0	
Atkinson, G. and D. Davenne (2007). "Relationships between sleep, physical activity and human health." Physiology & Behavior 90(2-3): 229-235.	1	1	0	0	0	
Atkinson, G., et al. (2007). "Exercise as a synchroniser of human circadian rhythms: an update and discussion of the methodological problems." European Journal of Applied Physiology 99(4): 331-341.	1	0	0	0	0	
Atkinson, G., et al. (2008). "Exercise, energy balance and the shift worker." Sports Medicine 38(8): 671-685.	1	1	1	1	0	NARRATIVE
Atkinson, G. and T. Reilly (1996). "Circadian Variation in sports performance." Sports Medicine 21(4): 292-312.	1	0	0	0	0	

Barion, A. and P. C. Zee (2007). "A clinical approach to circadian rhythm sleep disorders." Sleep Medicine 8(6): 566-577.	1	0	0	0	0	
Baron, K. G. and K. J. Reid (2014). "Circadian misalignment and health." International Review of Psychiatry 26(2): 139-154.	1	0	0	0	0	
Bayon, V., et al. (2014). "Sleep debt and obesity." Annals of Medicine 46(5): 264-272.	1	0	0	0	0	
Beckett, M. and L. C. Roden (2009). "Mechanisms by which circadian rhythm disruption may lead to cancer." South African Journal of Science 105(11-12): 415-420.	1	0	0	0	0	
Bonde, J. P., et al. (2013). "Miscarriage and occupational activity: a systematic review and meta-analysis regarding shift work, working hours, lifting, standing, and physical workload." Scandinavian Journal of Work Environment & Health 39(4): 325-334.	1	1	0	0	0	
Bonzini, M., et al. (2007). "Risk of prematurity, low birthweight and pre-eclampsia in relation to working hours and physical activities: a systematic review." Occupational and Environmental Medicine 64(4): 228-243.	1	0	0	0	0	
Bonzini, M., et al. (2011). "Shift work and pregnancy outcomes: a systematic review with meta-analysis of currently available epidemiological studies." Bjog-an International Journal of Obstetrics and Gynaecology 118(12): 1429-1437.	1	0	0	0	0	
Booker, L. A., et al. (2018). "Individual vulnerability to insomnia, excessive sleepiness and shift work disorder amongst healthcare shift workers. A systematic review." Sleep Medicine Reviews 41: 220-233.	1	0	0	0	0	
Borel, A.-L. (2019). "Sleep Apnea and Sleep Habits: Relationships with Metabolic Syndrome." Nutrients 11(11).	1	0	0	0	0	
Bovbjerg, M. L. (2019). "Current Resources for Evidence-Based Practice, July 2019." Jognn-Journal of Obstetric Gynecologic and Neonatal Nursing 48(4): 478-491.	1	0	0	0	0	
Brubakk, A. O., et al. (2014). "Saturation Diving; Physiology and Pathophysiology." Comprehensive Physiology 4(3): 1229-1272.	1	0	0	0	0	
Buxton, O. M., et al. (1997). "Acute and delayed effects of exercise on human melatonin secretion." Journal of Biological Rhythms 12(6): 568-574.	1	0	0	0	0	
Cai, C., et al. (2020). "The impact of occupational activities during pregnancy on pregnancy outcomes: a systematic review and	1	0	0	0	0	

metaanalysis." American Journal of Obstetrics and Gynecology 222(3): 224-238.						
Chappel, S. E., et al. (2017). "Nurses' occupational physical activity levels: A systematic review." International Journal of Nursing Studies 73: 52-62.	1	1	0	0	0	
Chau, Y. M., et al. (2014). "Night Work and the Reproductive Health of Women: An Integrated Literature Review." Journal of Midwifery & Womens Health 59(2): 113-126.	1	0	0	0	0	
Choi, Y., et al. (2020). "Re-Setting the Circadian Clock Using Exercise against Sarcopenia." International Journal of Molecular Sciences 21(9).	1	0	0	0	0	
Copinschi, G., et al. (2000). "Pathophysiology of human circadian rhythms." Novartis Foundation symposium 227: 143-157; discussion 157-162.	1	0	0	0	0	
Daly, A. A., et al. (2021). "A Review of Modifiable Risk Factors in Young Women for the Prevention of Breast Cancer." Breast Cancer-Targets and Therapy 13: 241-257.	1	0	0	0	0	
Davis, M. P. and H. W. Goforth (2014). "Long-term and Short-term Effects of Insomnia in Cancer and Effective Interventions." Cancer Journal 20(5): 330-344.	1	0	0	0	0	
De Mello, M. T., et al. (2020). "Sleep and COVID-19: considerations about immunity, pathophysiology, and treatment." Sleep Science 13(3): 199-209.	1	0	0	0	0	
Demou, E., et al. (2018). "Group-based healthy lifestyle workplace interventions for shift workers: a systematic review." Scandinavian Journal of Work Environment & Health 44(6): 568-584.	1	1	0	0	0	
Dollander, M. (2002). "Etiology of adults's insomnia." Encephale-Revue De Psychiatrie Clinique Biologique Et Therapeutique 28(6): 493-502.	1	0	0	0	0	
Dorrian, J., et al. (2019). Self-regulation and social behavior during sleep deprivation. Sleep Deprivation and Cognition. H. P. A. VanDongen, P. Whitney, J. M. Hinson, K. A. Honn and M. W. L. Chee. 246: 73-110.	1	0	0	0	0	
Engel, C. L., et al. (2018). "Work and Female Breast Cancer: The State of the Evidence, 2002-2017." New Solutions-a Journal of Environmental and Occupational Health Policy 28(1): 55-78.	1	0	0	0	0	

Fernandez, R. C., et al. (2016). "Fixed or Rotating Night Shift Work Undertaken by Women: Implications for Fertility and Miscarriage." Seminars in Reproductive Medicine 34(2): 74-82.	1	0	0	0	0	
Fernandez, R. C., et al. (2016). "Fixed or Rotating Night Shift Work Undertaken by Women: Implications for Fertility and Miscarriage." Seminars in Reproductive Medicine 34(2): 74-82.	1	0	0	0	0	
Figa-Talamanca, I. (2000). "Reproductive problems among women health care workers: Epidemiologic evidence and preventive strategies." Epidemiologic Reviews 22(2): 249-260.	1	0	0	0	0	
Foret, J. (1992). "CONTRIBUTION OF CHRONOBIOLOGY TO THE CURRENT ISSUES OF SHIFTWORK." Travail Humain 55(3): 237-257.	1	0	0	0	0	
Funderburk, L., et al. (2020). "Healthy Behaviors through Behavioral Design-Obesity Prevention." International Journal of Environmental Research and Public Health 17(14).	1	0	0	0	0	
Gamble, K. L. and M. E. Young (2013). "Metabolism as an integral cog in the mammalian circadian clockwork." Critical Reviews in Biochemistry and Molecular Biology 48(4): 317-331.	1	0	0	0	0	
Gan, Y., et al. (2018). "Association between shift work and risk of prostate cancer: a systematic review and meta-analysis of observational studies." Carcinogenesis 39(2): 87-97.	1	0	0	0	0	
Gerhart-Hines, Z. and M. A. Lazar (2015). "Circadian Metabolism in the Light of Evolution." Endocrine Reviews 36(3): 289-304.	1	0	0	0	0	
Golubnitschaja, O., et al. (2016). "Breast cancer epidemic in the early twenty-first century: evaluation of risk factors, cumulative questionnaires and recommendations for preventive measures." Tumor Biology 37(10): 12941-12957.	1	0	0	0	0	
Good, C. H., et al. (2020). "Sleep in the United States Military." Neuropsychopharmacology 45(1): 176-191.	1	0	0	0	0	
Gupta, C. C., et al. (2019). "The factors influencing the eating behaviour of shiftworkers: what, when, where and why." Industrial Health 57(4): 419-453.	1	0	0	0	0	
Hittle, B. M. and G. L. Gillespie (2018). "Identifying shift worker chronotype: implications for health." Industrial Health 56(6): 512-523.	1	0	0	0	0	

Horakova, D., et al. (2018). "Risks and protective factors for triple negative breast cancer with a focus on micronutrients and infections." Biomedical Papers-Olomouc 162(2): 83-89.	1	0	0	0	0	
Hruby, A., et al. (2016). "Determinants and Consequences of Obesity." American Journal of Public Health 106(9): 1656-1662.	1	0	0	0	0	
Hwang, W. J. and O. Hong (2012). "Work-related cardiovascular disease risk factors using a socioecological approach: implications for practice and research." European Journal of Cardiovascular Nursing 11(1): 114-126.	1	0	0	0	0	
Kang, J., et al. (2020). "Sleep quality among shift-work nurses: A systematic review and meta-analysis." Applied Nursing Research 52.	1	0	0	0	0	
Kanikowska, D., et al. (2015). "Contribution of daily and seasonal biorhythms to obesity in humans." International Journal of Biometeorology 59(4): 377-384.	1	0	0	0	0	
Kelly, M. and J. Wills (2018). "Systematic review: What works to address obesity in nurses?" Occupational Medicine-Oxford 68(4): 228-238.	1	1	0	0	0	
Kenny, G. P., et al. (2016). "Age, human performance, and physical employment standards." Applied Physiology Nutrition and Metabolism 41(6): S92-S107.	1	0	0	0	0	
Kernbach, M. E., et al. (2018). "Dim light at night: physiological effects and ecological consequences for infectious disease." Integrative and Comparative Biology 58(5): 995-1007.	1	0	0	0	0	
Kervezee, L., et al. (2020). "Metabolic and cardiovascular consequences of shift work: The role of circadian disruption and sleep disturbances." European Journal of Neuroscience 51(1): 396-412.	1	0	0	0	0	
Kettner, N. M., et al. (2014). "Circadian gene variants in cancer." Annals of Medicine 46(4): 208-220.	1	0	0	0	0	
Knutsson, A. and H. Boggild (2000). "Shiftwork and cardiovascular disease: review of disease mechanisms." Reviews on environmental health 15(4): 359-372.	1	1	1	1	0	NO EVALUATION OF THE ASSOCIATION BETWEEN SW AND OUTCOME
Kozlowska, L., et al. (2019). "HEALTH RISK IN TRANSPORT WORKERS PART II. DIETARY COMPOUNDS AS MODULATORS OF OCCUPATIONAL EXPOSURE TO CHEMICALS." International Journal of Occupational Medicine and Environmental Health 32(4): 441-464.	1	0	0	0	0	

Kristensen, T. S. (1989). "Work environment and cardiovascular diseases. A short review of the literature." Journal of UOEH 11 Suppl: 120-133.	1	1	0	0	0	
Krstev, S. and A. Knutsson (2019). "Occupational Risk Factors for Prostate Cancer: A Meta-analysis." Journal of Cancer Prevention 24(2): 91-111.	1	0	0	0	0	
Kyriacou, C. P. and M. H. Hastings (2010). "Circadian clocks: genes, sleep, and cognition." Trends in Cognitive Sciences 14(6): 259-267.	1	0	0	0	0	
Laermans, J. and I. Depoortere (2016). "Chronobesity: role of the circadian system in the obesity epidemic." Obesity Reviews 17(2): 108-125.	1	0	0	0	0	
Li, W., et al. (2021). "Association of noise exposure with risk of metabolic syndrome: Evidence from 44,698 individuals." Diabetes research and clinical practice 178: 108944-108944.	1	0	0	0	0	
Mactaggart, F., et al. (2016). "Examining health and well-being outcomes associated with mining activity in rural communities of high-income countries: A systematic review." Australian Journal of Rural Health 24(4): 230-237.	1	0	0	0	0	
Moreno, C. R. C., et al. (2019). "Working Time Society consensus statements: Evidence-based effects of shift work on physical and mental health." Industrial Health 57(2): 139-157.	1	1	0	0	0	
Morris, C. J., et al. (2012). The impact of the circadian timing system on cardiovascular and metabolic function. Neurobiology of Circadian Timing. A. Kalsbeek, M. Merrow, T. Roenneberg and R. G. Foster. 199: 337-358.	1	0	0	0	0	
Mozurkewich, E. L., et al. (2000). "Working conditions and adverse pregnancy outcome: A meta-analysis." Obstetrics and Gynecology 95(4): 623-635.	1	0	0	0	0	
Nea, F. M., et al. (2015). "Dietary and lifestyle habits and the associated health risks in shift workers." Nutrition Research Reviews 28(2): 143-166.	1	1	1	1	0	NARRATIVE
Nedeltcheva, A. V. and F. A. J. L. Scheer (2014). "Metabolic effects of sleep disruption, links to obesity and diabetes." Current Opinion in Endocrinology Diabetes and Obesity 21(4): 293-298.	1	0	0	0	0	
Neil-Sztramko, S. E., et al. (2014). "Health-related interventions among night shift workers: a critical review of the literature." Scandinavian Journal of Work Environment & Health 40(6): 543-556.	1	1	0	0	0	

Palmer, K. T., et al. (2013). "Pregnancy: occupational aspects of management: concise guidance." Clinical Medicine 13(1): 75-79.	1	0	0	0	0	
Palmer, K. T., et al. (2013). "Work activities and risk of prematurity, low birth weight and pre-eclampsia: an updated review with meta-analysis." Occupational and Environmental Medicine 70(4): 213-222.	1	0	0	0	0	
Pandalai, S. P., et al. (2013). "Conceptual heuristic models of the interrelationships between obesity and the occupational environment." Scandinavian Journal of Work Environment & Health 39(3): 221-232.	1	0	0	0	0	
Peate, I. (2007). "Strategies for coping with shift work." Nursing standard (Royal College of Nursing (Great Britain) : 1987) 22(4): 42-45.	1	1	0	0	0	
Peter, R. and J. Siegrist (2000). "Psychosocial work environment and the risk of coronary heart disease." International Archives of Occupational and Environmental Health 73: S41-S45.	1	0	0	0	0	
Pittman-Polletta, B. R., et al. (2013). "The role of the circadian system in fractal neurophysiological control." Biological Reviews 88(4): 873-894.	1	0	0	0	0	
Polivka, J., Jr., et al. (2018). "Pregnancy-associated breast cancer: the risky status quo and new concepts of predictive medicine." Epma Journal 9(1): 1-13.	1	0	0	0	0	
Pot, G. K. (2018). "Sleep and dietary habits in the urban environment: the role of chrono-nutrition." Proceedings of the Nutrition Society 77(3): 189-198.	1	0	0	0	0	
Potter, G. D. M. and T. R. Wood (2020). "The Future of Shift Work: Circadian Biology Meets Personalised Medicine and Behavioural Science." Frontiers in Nutrition 7.	1	0	0	0	0	
Pukkala, E., et al. (2009). "Occupation and cancer - follow-up of 15 million people in five Nordic countries." Acta Oncologica 48(5): 646-790.	1	0	0	0	0	
Redlin, U. and N. Mrosovsky (1997). "Exercise and human circadian rhythms: What we know and what we need to know." Chronobiology International 14(2): 221-229.	1	1	0	0	0	
Reid, K. J., et al. (2004). "Circadian rhythm sleep disorders." Medical Clinics of North America 88(3): 631-+.	1	0	0	0	0	
Reid, K. J., et al. (2011). Cognition in circadian rhythm sleep disorders. Human Sleep and Cognition, Pt Ii: Clinical and Applied Research. H. P. A. VanDongen and G. A. Kerkhof. 190: 3-20.	1	0	0	0	0	

Reilly, T. (1990). "HUMAN CIRCADIAN-RHYTHMS AND EXERCISE." Critical Reviews in Biomedical Engineering 18(3): 165-180.	1	1	0	0	0	
Reilly, T., et al. (2006). "Some chronobiological considerations related to physical exercise." La Clinica terapeutica 157(3): 249-264.	1	1	0	0	0	
Reilly, T. and J. Waterhouse (2007). "Altered sleep-wake cycles and food intake: The Ramadan model." Physiology & Behavior 90(2-3): 219-228.	1	0	0	0	0	
Reinberg, A., et al. (2015). "Chronobiologic perspectives of black time-Accident risk is greatest at night: An opinion paper." Chronobiology International 32(7): 1005-1018.	1	0	0	0	0	
Rosa, R. R., et al. (1990). "INTERVENTION FACTORS FOR PROMOTING ADJUSTMENT TO NIGHTWORK AND SHIFTWORK." Occupational Medicine-State of the Art Reviews 5(2): 391-414.	1	1	0	0	0	
Samuelsson, L. B., et al. (2018). "Sleep and circadian disruption and incident breast cancer risk: An evidence-based and theoretical review." Neuroscience and Biobehavioral Reviews 84: 35-48.	1	0	0	0	0	
Schilperoort, M., et al. (2020). "Time for Novel Strategies to Mitigate Cardiometabolic Risk in Shift Workers." Trends in Endocrinology and Metabolism 31(12): 952-964.	1	1	1	1	0	NO EVALUATION OF THE ASSOCIATION BETWEEN SW AND OUTCOME
Shephard, R. J. (2017). "Peptic Ulcer and Exercise." Sports Medicine 47(1): 33-40.	1	0	0	0	0	
Shi, Y. and S. Zhong (2014). "From genomes to societies: a holistic view of determinants of human health." Current Opinion in Biotechnology 28: 134-142.	1	0	0	0	0	
Shochat, T. (2012). "Impact of lifestyle and technology developments on sleep." Nature and science of sleep 4: 19-31.	1	0	0	0	0	
Shrestha, N., et al. (2016). "The Impact of Obesity in the Workplace: a Review of Contributing Factors, Consequences and Potential Solutions." Current Obesity Reports 5(3): 344-360.	1	0	0	0	0	
Sleddens, E. F. C., et al. (2015). "Correlates of dietary behavior in adults: an umbrella review." Nutrition Reviews 73(8): 477-499.	1	0	0	0	0	
Smith, E. C., et al. (2019). "Exploring the Physical and Mental Health Challenges Associated with Emergency Service Call-Taking and Dispatching: A Review of the Literature." Prehospital and Disaster Medicine 34(6): 619-624.	1	1	1	1	0	NO EVALUATION OF THE ASSOCIATION BETWEEN SW AND OUTCOME

Smith, L., et al. (2016). "A review of occupational physical activity and sedentary behaviour correlates." Occupational Medicine-Oxford 66(3): 185-192.	1	1	0	0	0	
Smolensky, M. H. and A. Reinberg (1990). "CLINICAL CHRONOBIOLOGY - RELEVANCE AND APPLICATIONS TO THE PRACTICE OF OCCUPATIONAL-MEDICINE." Occupational Medicine-State of the Art Reviews 5(2): 239-272.	1	0	0	0	0	
Soteriades, E. S., et al. (2011). "Cardiovascular Disease in US Firefighters A Systematic Review." Cardiology in Review 19(4): 202-215.	1	0	0	0	0	
Steenland, K. (1996). "Epidemiology of occupation and coronary heart disease: Research agenda." American Journal of Industrial Medicine 30(4): 495-499.	1	0	0	0	0	
Sun, M., et al. (2018). "Meta-analysis on shift work and risks of specific obesity types." Obesity Reviews 19(1): 28-40.	1	1	0	0	0	
Sun, X., et al. (2020). "The association between sleep chronotype and obesity among black and white participants of the Bogalusa Heart Study." Chronobiology International 37(1): 123-134.	1	0	0	0	0	
Swanson, G. R. and H. J. Burgess (2017). "Sleep and Circadian Hygiene and Inflammatory Bowel Disease." Gastroenterology Clinics of North America 46(4): 881-+.	1	0	0	0	0	
Tahara, Y. and S. Shibata (2018). "Entrainment of the mouse circadian clock: Effects of stress, exercise, and nutrition." Free Radical Biology and Medicine 119: 129-138.	1	0	0	0	0	
Tanner, C. M. (2010). "Advances in Environmental Epidemiology." Movement Disorders 25(3): S58-S62.	1	0	0	0	0	
Thorpy, M. J., et al. (2007). "Patient-management strategies." American Journal of Managed Care 13(6): S140-S147.	1	0	0	0	0	
Togo, F. and M. Takahashi (2009). "Heart Rate Variability in Occupational Health -A Systematic Review." Industrial Health 47(6): 589-602.	1	0	0	0	0	
Turek, F. W. and O. Vanreeth (1989). "USE OF BENZODIAZEPINES TO MANIPULATE THE CIRCADIAN CLOCK REGULATING BEHAVIORAL AND ENDOCRINE RHYTHMS." Hormone Research 31(1-2): 59-65.	1	0	0	0	0	
van Drongelen, A., et al. (2011). "The effects of shift work on body weight change - a systematic review of longitudinal studies." Scandinavian Journal of Work Environment & Health 37(4): 263-275.	1	1	0	0	0	

van Melick, M. J. G. J., et al. (2014). "Shift work, long working hours and preterm birth: a systematic review and meta-analysis." International Archives of Occupational and Environmental Health 87(8): 835-849.	1	0	0	0	0	
Vincent, G. E., et al. (2018). "Sleep in wildland firefighters: what do we know and why does it matter?" International Journal of Wildland Fire 27(2): 73-84.	1	0	0	0	0	
Waldman, H. S., et al. (2020). "Time-restricted feeding for the prevention of cardiometabolic diseases in high-stress occupations: a mechanistic review." Nutrition Reviews 78(6): 459-464.	1	0	0	0	0	
Westerterp-Plantenga, M. S. (2016). "Sleep, circadian rhythm and body weight: parallel developments." Proceedings of the Nutrition Society 75(4): 431-439.	1	0	0	0	0	
Wirth, M., et al. (2013). "The epidemiology of cancer among police officers." American Journal of Industrial Medicine 56(4): 439-453.	1	0	0	0	0	
Wosu, A. C., et al. (2013). "Correlates of cortisol in human hair: implications for epidemiologic studies on health effects of chronic stress." Annals of Epidemiology 23(12): 797-811.	1	0	0	0	0	
Yamada, Y., et al. (2002). "Prevention of weight gain and obesity in occupational populations: A new target of health promotion services at worksites." Journal of Occupational Health 44(6): 373-384.	1	0	0	0	0	
Yu, E., et al. (2016). "Diet, Lifestyle, Biomarkers, Genetic Factors, and Risk of Cardiovascular Disease in the Nurses' Health Studies." American Journal of Public Health 106(9): 1616-1623.	1	1	0	0	0	
Zee, P. C. and P. Manthena (2007). "The brain's master circadian clock: Implications and opportunities for therapy of sleep disorders." Sleep Medicine Reviews 11(1): 59-70.	1	0	0	0	0	
Zhang, Q., et al. (2020). "Association between shift work and obesity among nurses: A systematic review and meta-analysis." International Journal of Nursing Studies 112.	1	1	0	0	0	
Zimmerman, F. H. (2012). "Cardiovascular Disease and Risk Factors in Law Enforcement Personnel: A Comprehensive Review." Cardiology in Review 20(4): 159-166.	1	0	0	0	0	

OCCUPATIONAL PSYCHOSOCIAL STRESSORS	IDENTIFICATION	SCREENING		ELIGIBILITY	INCLUDED	REASON FOR EXCLUSION
	SEARCH / REFERENCE LIST	TITLE	ABSTRACT	FULL TEXT	FULL TEST	
Abbott, S. M., et al. (2014). "Sleep disorders in perinatal women." Best Practice & Research Clinical Obstetrics & Gynaecology 28(1): 159-168.	1	0	0	0	0	
Alfonsi, V., et al. (2021). "Sleep-Related Problems in Night Shift Nurses: Towards an Individualized Interventional Practice." Frontiers in Human Neuroscience 15.	1	0	0	0	0	
Amiri, S. and S. Behnezhad (2020). "Job strain and mortality ratio: a systematic review and meta-analysis of cohort studies." Public Health 181: 24-33.	1	0	0	0	0	
Ancoli-Israel, S., et al. (2003). "The role of actigraphy in the study of sleep and circadian rhythms." Sleep 26(3): 342-392.	1	0	0	0	0	
Archer, S. N. and H. Oster (2015). "How sleep and wakefulness influence circadian rhythmicity: effects of insufficient and mistimed sleep on the animal and human transcriptome." Journal of Sleep Research 24(5): 476-493.	1	0	0	0	0	
Asghari, M. H., et al. (2018). "Does the use of melatonin overcome drug resistance in cancer chemotherapy?" Life Sciences 196: 143-155.	1	0	0	0	0	
Astiz, M. and H. Oster (2018). "Perinatal Programming of Circadian Clock-Stress Crosstalk." Neural Plasticity 2018.	1	0	0	0	0	
Baksa, D., et al. (2019). "Circadian Variation of Migraine Attack Onset: A Review of Clinical Studies." Biomed Research International 2019.	1	0	0	0	0	
Bambra, C. L., et al. (2008). "Shifting schedules - The health effects of reorganizing shift work." American Journal of Preventive Medicine 34(5): 427-434.	1	1	0	0	0	
Barger, L. K., et al. (2018). "Effect of Fatigue Training on Safety, Fatigue, and Sleep in Emergency Medical Services Personnel and Other Shift Workers: A Systematic Review and Meta-Analysis." Prehospital Emergency Care 22: 58-68.	1	0	0	0	0	
Barthe, B., et al. (2004). "Work activity during night shifts: A review of 25 years of research and perspectives." Travail Humain 67(1): 41-61.	1	1	0	0	0	

Bartman, C. M. and T. Eekle (2019). "Circadian-Hypoxia Link and its Potential for Treatment of Cardiovascular Disease." Current Pharmaceutical Design 25(10): 1075-1090.	1	0	0	0	0	
Behn, C. and N. De Gregorio (2020). "Melatonin Relations with Energy Metabolism as Possibly Involved in Fatal Mountain Road Traffic Accidents." International Journal of Molecular Sciences 21(6).	1	0	0	0	0	
Blok, M. M. and M. P. de Looze (2011). "What is the evidence for less shift work tolerance in older workers?" Ergonomics 54(3): 221-232.	1	1	0	0	0	
Boga, J. A., et al. (2019). "Therapeutic potential of melatonin related to its role as an autophagy regulator: A review." Journal of Pineal Research 66(1).	1	0	0	0	0	
Boggild, H. and A. Knutsson (1999). "Shift work, risk factors and cardiovascular disease." Scandinavian Journal of Work Environment & Health 25(2): 85-99.	1	1	1	1	0	NO EVALUATION OF THE ASSOCIATION BETWEEN SW AND OUTCOME
Bohle, P., et al. (2010). "TIME TO CALL IT QUITS? THE SAFETY AND HEALTH OF OLDER WORKERS." International Journal of Health Services 40(1): 23-41.	1	0	0	0	0	
Broussard, J. and M. J. Brady (2010). "The impact of sleep disturbances on adipocyte function and lipid metabolism." Best Practice & Research Clinical Endocrinology & Metabolism 24(5): 763-773.	1	0	0	0	0	
Brown, J. P., et al. (2020). "Mental Health Consequences of Shift Work: An Updated Review." Current Psychiatry Reports 22(2).	1	1	1	1	0	NO EVALUATION OF THE ASSOCIATION BETWEEN SW AND OUTCOME
Brown-DeGagne, A. M. and G. A. Eskes (1998). "Turning body time to shift time." The Canadian nurse 94(8): 51-52.	1	0	0	0	0	
Brubakk, A. O., et al. (2014). "Saturation Diving; Physiology and Pathophysiology." Comprehensive Physiology 4(3): 1229-1272.	1	0	0	0	0	
Bruhl, A. B. and B. J. Sahakian (2016). Drugs, games, and devices for enhancing cognition: implications for work and society. Year in Cognitive Neuroscience. A. Kingstone and M. B. Miller. 1369: 195-217.	1	0	0	0	0	
Brzecka, A., et al. (2020). "The Association of Sleep Disorders, Obesity and Sleep-Related Hypoxia with Cancer." Current Genomics 21(6): 444-453.	1	0	0	0	0	
Bubenik, G. A., et al. (1998). "Prospects of the clinical utilization of melatonin." Biological Signals and Receptors 7(4): 195-219.	1	0	0	0	0	

Buss, J. (2012). "Associations Between Obesity and Stress and Shift Work Among Nurses." Workplace Health & Safety 60(10): 453-458.	1	1	0	0	0	
Buxton, O. M., et al. (1997). "Acute and delayed effects of exercise on human melatonin secretion." Journal of Biological Rhythms 12(6): 568-574.	1	0	0	0	0	
Cardinali, D. P., et al. (2011). "The use of chronobiotics in the resynchronization of the sleep/wake cycle. Therapeutical application in the early phases of Alzheimer's disease." Recent patents on endocrine, metabolic & immune drug discovery 5(2): 80-90.	1	0	0	0	0	
Caruso, C. C. (2014). "Negative Impacts of Shiftwork and Long Work Hours." Rehabilitation Nursing 39(1): 16-25.	1	1	1	1	0	NO EVALUATION OF THE ASSOCIATION BETWEEN SW AND OUTCOME
Chuang, C.-H., et al. (2016). "Burnout in the intensive care unit professionals A systematic review." Medicine 95(50).	1	1	0	0	0	
Clougherty, J. E., et al. (2010). Work and its role in shaping the social gradient in health. Biology of Disadvantage: Socioeconomic Status and Health. N. E. Adler and J. Stewart. 1186: 102-124.	1	0	0	0	0	
Corlett, E. N. and P. Shipley (1989). "Stressful conditions of work: the participative ergonomics approach." Journal of UOEH 11 Suppl: 541-548.	1	0	0	0	0	
Costa, G. (2010). "Shift work and health: current problems and preventive actions." Safety and health at work 1(2): 112-123.	1	1	1	1	0	NO EVALUATION OF THE ASSOCIATION BETWEEN SW AND OUTCOME
Culebras, A. (1992). "UPDATE ON DISORDERS OF SLEEP AND THE SLEEP-WAKE CYCLE." Psychiatric Clinics of North America 15(2): 467-489.	1	0	0	0	0	
Culpepper, L. (2010). "The social and economic burden of shift-work disorder." Journal of Family Practice 59(1): S3-S11.	1	0	0	0	0	
da Silva, F. R., et al. (2020). "Does the compromised sleep and circadian disruption of night and shiftworkers make them highly vulnerable to 2019 coronavirus disease (COVID-19)?" Chronobiology International 37(5): 607-617.	1	0	0	0	0	
Damasio Silva, D. d. S., et al. (2015). "Depression and suicide risk among Nursing professionals: an integrative review." Revista Da Escola De Enfermagem Da Usp 49(6): 1023-1031.	1	1	0	0	0	
de Cordova, P. B., et al. (2012). "Twenty-four/seven: a mixed-method systematic review of the off-shift literature." Journal of Advanced Nursing 68(7): 1454-1468.	1	1	1	1	0	NO EVALUATION OF THE ASSOCIATION BETWEEN SW AND OUTCOME

De Mello, M. T., et al. (2020). "Sleep and COVID-19: considerations about immunity, pathophysiology, and treatment." Sleep Science 13(3): 199-209.	1	0	0	0	0	
Delagrange, P., et al. (2003). "Therapeutic perspectives for melatonin agonists and antagonists." Journal of Neuroendocrinology 15(4): 442-448.	1	0	0	0	0	
Descatha, A., et al. (2020). "The effect of exposure to long working hours on stroke: A systematic review and meta -analysis from the WHO/ILO Joint Estimates of the Work -related Burden of Disease and Injury." Environment International 142.	1	1	0	0	0	
Di Muzio, M., et al. (2019). "Can nurses' shift work jeopardize the patient safety? A systematic review." European Review for Medical and Pharmacological Sciences 23(10): 4507-4519.	1	0	0	0	0	
Diene, E., et al. (2012). "Cardiovascular diseases and psychosocial factors at work." Archives of Cardiovascular Diseases 105(1): 33-39.	1	1	0	0	0	
Dohrmann, S. B. and A. Leppin (2017). "Determinants of seafarers' fatigue: a systematic review and quality assessment." International Archives of Occupational and Environmental Health 90(1): 13-37.	1	0	0	0	0	
Dollander, M. (2002). "Etiology of adults's insomnia." Encephale-Revue De Psychiatrie Clinique Biologique Et Therapeutique 28(6): 493-502.	1	0	0	0	0	
Dorevitch, S. and L. Forst (2000). "The occupational hazards of emergency physicians." American Journal of Emergency Medicine 18(3): 300-311.	1	0	0	0	0	
Dumitru, C., et al. (2018). "Metabolic Adaptations of CD4(+) T Cells in inflammatory Disease." Frontiers in Immunology 9.	1	0	0	0	0	
Efinger, J., et al. (1995). "Understanding circadian rhythms: a holistic approach to nurses and shift work." Journal of holistic nursing : official journal of the American Holistic Nurses' Association 13(4): 306-322.	1	0	0	0	0	
Eismann, E. A., et al. (2010). "Circadian effects in cancer-relevant psychoneuroendocrine and immune pathways." Psychoneuroendocrinology 35(7): 963-976.	1	0	0	0	0	
El Mlili, N., et al. (2021). "Hair Cortisol Concentration as a Biomarker of Sleep Quality and Related Disorders." Life-Basel 11(2).	1	0	0	0	0	

Elder, G. J., et al. (2014). "The cortisol awakening response - Applications and implications for sleep medicine." Sleep Medicine Reviews 18(3): 215-224.	1	0	0	0	0	
Engel, C. L., et al. (2018). "Work and Female Breast Cancer: The State of the Evidence, 2002-2017." New Solutions-a Journal of Environmental and Occupational Health Policy 28(1): 55-78.	1	0	0	0	0	
Estabrooks, C. A., et al. (2009). "Effects of shift length on quality of patient care and health provider outcomes: systematic review." Quality & Safety in Health Care 18(3): 181-188.	1	1	0	0	0	
Faraut, B., et al. (2017). "Napping: A public health issue. From epidemiological to laboratory studies." Sleep Medicine Reviews 35: 85-100.	1	0	0	0	0	
Faraut, B., et al. (2013). "Neuroendocrine, immune and oxidative stress in shift workers." Sleep Medicine Reviews 17(6): 433-444.	1	0	0	0	0	
Ferguson, S. A., et al. (2016). "On-call work: To sleep or not to sleep? It depends." Chronobiology International 33(6): 678-684.	1	0	0	0	0	
Ferri, G. M., et al. (2019). "Healthy Diet and Reduction of Chronic Disease Risks of Night Shift Workers." Current Medicinal Chemistry 26(19): 3521-3541.	1	0	0	0	0	
Figa-Talamanca, I. (2000). "Reproductive problems among women health care workers: Epidemiologic evidence and preventive strategies." Epidemiologic Reviews 22(2): 249-260.	1	0	0	0	0	
Figa-Talamanca, I. (2006). "Occupational risk factors and reproductive health of women." Occupational Medicine-Oxford 56(8): 521-531.	1	0	0	0	0	
Fink, A. M. (2020). "MEASURING THE EFFECTS OF NIGHT-SHIFT WORK ON CARDIAC AUTONOMIC MODULATION: AN APPRAISAL OF HEART RATE VARIABILITY METRICS." International Journal of Occupational Medicine and Environmental Health 33(4): 409-425.	1	0	0	0	0	
Fischer, F. M., et al. (2019). "Working Time Society consensus statements: Psychosocial stressors relevant to the health and wellbeing of night and shift workers." Industrial Health 57(2): 175-183.	1	1	1	1	0	NARRATIVE
Fodale, V., et al. (2008). "Genotoxic effects of anesthetic agents." Expert Opinion on Drug Safety 7(4): 447-458.	1	0	0	0	0	

Foret, J. (1992). "CONTRIBUTION OF CHRONOBIOLOGY TO THE CURRENT ISSUES OF SHIFTWORK." Travail Humain 55(3): 237-257.	1	0	0	0	0	
Forrestel, A. C., et al. (2017). "Chronomedicine and type 2 diabetes: shining some light on melatonin." Diabetologia 60(5): 808-822.	1	0	0	0	0	
Forsyth, C. B., et al. (2015). "Circadian rhythms, alcohol and gut interactions." Alcohol 49(4): 389-398.	1	0	0	0	0	
Foster, R. G. (2020). "Sleep, circadian rhythms and health." Interface Focus 10(3).	1	0	0	0	0	
Gadbois, C. (2004). "Psychosocial conflicts created by shift work patterns: Pending questions." Travail Humain 67(1): 63-85.	1	1	0	0	0	
Gallant, A. R., et al. (2012). "The night-eating syndrome and obesity." Obesity Reviews 13(6): 528-536.	1	0	0	0	0	
Gannon, L. (1993). "MENOPAUSAL SYMPTOMS AS CONSEQUENCES OF DYSRHYTHMIA." Journal of Behavioral Medicine 16(4): 387-402.	1	0	0	0	0	
Garbarino, S., et al. (2019). "Sleep Quality among Police Officers: Implications and Insights from a Systematic Review and Meta-Analysis of the Literature." International Journal of Environmental Research and Public Health 16(5).	1	0	0	0	0	
Gehlert, S., et al. (2020). "Shift Work and Breast Cancer." International Journal of Environmental Research and Public Health 17(24).	1	0	0	0	0	
Geiger-Brown, J. and J. Lipscomb (2010). "The health care work environment and adverse health and safety consequences for nurses." Annual review of nursing research 28: 191-231.	1	1	0	0	0	
Genario, K., et al. (2019). "The usefulness of melatonin in the field of obstetrics and gynecology." Pharmacological Research 147.	1	0	0	0	0	
Gestal, J. J. (1987). "OCCUPATIONAL HAZARDS IN HOSPITALS - ACCIDENTS, RADIATION, EXPOSURE TO NOXIOUS CHEMICALS, DRUG-ADDICTION AND PSYCHIC PROBLEMS, AND ASSAULT." British Journal of Industrial Medicine 44(8): 510-520.	1	0	0	0	0	
Gilmour, D. (2000). "Risk for the new or expectant mother working in the perioperative environment." British journal of perioperative nursing : the journal of the National Association of Theatre Nurses 10(6): 306-310.	1	0	0	0	0	
Gitto, E., et al. (2011). "Update on the use of melatonin in pediatrics." Journal of Pineal Research 50(1): 21-28.	1	0	0	0	0	

Glozier, N., et al. (2013). "Psychosocial risk factors for coronary heart disease." Medical Journal of Australia 199(3): 179-180.	1	1	0	0	0	
Godderis, L., et al. (2018). "WHO/ILO work-related burden of disease and injury: Protocol for systematic reviews of exposure to long working hours and of the effect of exposure to long working hours on alcohol consumption and alcohol use disorders." Environment International 120: 22-33.	1	0	0	0	0	
Golding, S. E., et al. (2017). "Exploring the psychological health of emergency dispatch centre operatives: a systematic review and narrative synthesis." Peerj 5.	1	1	0	0	0	
Gonnissen, H. K. J., et al. (2013). "Chronobiology, endocrinology, and energy- and food-reward homeostasis." Obesity Reviews 14(5): 405-416.	1	0	0	0	0	
Gonzalez-Gonzalez, A., et al. (2018). "Melatonin: A Molecule for Reducing Breast Cancer Risk." Molecules 23(2).	1	0	0	0	0	
Good, C. H., et al. (2020). "Sleep in the United States Military." Neuropsychopharmacology 45(1): 176-191.	1	0	0	0	0	
Goulet, L. and G. Theriault (1987). "ASSOCIATION BETWEEN SPONTANEOUS-ABORTION AND ERGONOMIC FACTORS - A LITERATURE-REVIEW OF THE EPIDEMIOLOGIC EVIDENCE." Scandinavian Journal of Work Environment & Health 13(5): 399-403.	1	0	0	0	0	
Gozal, D., et al. (2016). "Obstructive sleep apnea and cancer: Epidemiologic links and theoretical biological constructs." Sleep Medicine Reviews 27: 43-55.	1	0	0	0	0	
Guandalini, L. S., et al. (2020). "Analysis of the evidence of related factors, associated conditions and at-risk populations of the NANDA-I nursing diagnosis insomnia." International Journal of Nursing Sciences 7(4): 376-386.	1	0	0	0	0	
Hall, S. J., et al. (2017). "The effect of working on-call on stress physiology and sleep: A systematic review." Sleep Medicine Reviews 33: 79-87.	1	0	0	0	0	
Hanikoglu, A., et al. (2018). "A Review on Melatonin's Effects in Cancer: Potential Mechanisms." Anti-Cancer Agents in Medicinal Chemistry 18(7): 985-992.	1	0	0	0	0	
Hansen, A. M., et al. (2009). "A Review of the Effect of the Psychosocial Working Environment on Physiological Changes in Blood and Urine." Basic & Clinical Pharmacology & Toxicology 105(2): 73-83.	1	0	0	0	0	

Hardeland, R. (2014). "Melatonin, Noncoding RNAs, Messenger RNA Stability and Epigenetics-Evidence, Hints, Gaps and Perspectives." International Journal of Molecular Sciences 15(10): 18221-18252.	1	0	0	0	0	
Harma, M. (2006). "Workhours in relation to work stress, recovery and health." Scandinavian Journal of Work Environment & Health 32(6): 502-514.	1	1	1	1	0	NARRATIVE
Harma, M. and I. Kandolin (2001). "Shiftwork, age and well-being: recent developments and future perspectives." Journal of human ergology 30(1-2): 287-293.	1	1	0	0	0	
Haupt, S., et al. (2021). "Eat, Train, Sleep-Retreat? Hormonal Interactions of Intermittent Fasting, Exercise and Circadian Rhythm." Biomolecules 11(4).	1	0	0	0	0	
Hedstrom, A. K., et al. (2015). "The Role of Environment and Lifestyle in Determining the Risk of Multiple Sclerosis." Current topics in behavioral neurosciences 26: 87-104.	1	0	0	0	0	
Holtzclaw, L., et al. (2021). "The Health of Health Care Professionals." American Journal of Lifestyle Medicine 15(2): 130-132.	1	0	0	0	0	
Honkalampi, K., et al. "Heart rate variability and chronotype - a systematic review." Chronobiology International.	1	0	0	0	0	
Hwang, W. J. and O. Hong (2012). "Work-related cardiovascular disease risk factors using a socioecological approach: implications for practice and research." European Journal of Cardiovascular Nursing 11(1): 114-126.	1	1	0	0	0	
Jensen, M. A., et al. (2016). "The effect of the number of consecutive night shifts on diurnal rhythms in cortisol, melatonin and heart rate variability (HRV): a systematic review of field studies." International Archives of Occupational and Environmental Health 89(4): 531-545.	1	0	0	0	0	
Jepsen, J. R., et al. (2015). "Seafarer fatigue: a review of risk factors, consequences for seafarers' health and safety and options for mitigation." International Maritime Health 66(2): 106-117.	1	0	0	0	0	
Jessiman-Perreault, G., et al. (2021). "Why Are Workplace Social Support Programs Not Improving the Mental Health of Canadian Correctional Officers? An Examination of the Theoretical Concepts Underpinning Support." International Journal of Environmental Research and Public Health 18(5).	1	0	0	0	0	

Johnson, J. V. and J. Lipscomb (2006). "Long working hours, occupational health and the changing nature of work organization." American Journal of Industrial Medicine 49(11): 921-929.	1	0	0	0	0	
Johnston, A., et al. (2016). "Review article: Staff perception of the emergency department working environment: Integrative review of the literature." Emergency Medicine Australasia 28(1): 7-26.	1	0	0	0	0	
Jones, L., et al. (2012). "Pain management for women in labour: an overview of systematic reviews." Cochrane Database of Systematic Reviews(3).	1	0	0	0	0	
Kales, S. N., et al. (2009). "Blood-Pressure in Firefighters, Police Officers, and Other Emergency Responders." American Journal of Hypertension 22(1): 11-20.	1	0	0	0	0	
Kalmbach, D. A., et al. (2018). "The impact of stress on sleep: Pathogenic sleep reactivity as a vulnerability to insomnia and circadian disorders." Journal of Sleep Research 27(6).	1	0	0	0	0	
Kaltenegger, H. C., et al. (2020). "Association of working conditions including digital technology use and systemic inflammation among employees: study protocol for a systematic review." Systematic Reviews 9(1).	1	0	0	0	0	
Kantermann, T., et al. (2012). Noisy and individual, but doable: Shift-work research in humans. Neurobiology of Circadian Timing. A. Kalsbeek, M. Merrow, T. Roenneberg and R. G. Foster. 199: 399-411.	1	0	0	0	0	
Karagozoglu, S. and N. Bingol (2008). "Sleep quality and job satisfaction of Turkish nurses." Nursing Outlook 56(6): 298-307.	1	0	0	0	0	
Karatsoreos, I. N. and B. S. McEwen (2011). "Psychobiological allostasis: resistance, resilience and vulnerability." Trends in Cognitive Sciences 15(12): 576-584.	1	0	0	0	0	
Kawada, T. (2011). "Noise and Health-Sleep Disturbance in Adults." Journal of Occupational Health 53(6): 413-416.	1	0	0	0	0	
Ke, D.-S. (2012). "Overwork, stroke, and karoshi-death from overwork." Acta neurologica Taiwanica 21(2): 54-59.	1	0	0	0	0	
Kecklund, G. and J. Axelsson (2016). "Health consequences of shift work and insufficient sleep." Bmj-British Medical Journal 355.	1	0	0	0	0	

Kennedy, H. D. (1990). "SHIFTWORK AS RELATED TO STRESS CLAIMS UNDER WORKERS COMPENSATION STATUTES." Occupational Medicine-State of the Art Reviews 5(2): 379-390.	1	1	0	0	0	
Kernbach, M. E., et al. (2018). "Dim light at night: physiological effects and ecological consequences for infectious disease." Integrative and Comparative Biology 58(5): 995-1007.	1	0	0	0	0	
Ketchesin, K. D., et al. (2020). "Mood-related central and peripheral clocks." European Journal of Neuroscience 51(1): 326-345.	1	0	0	0	0	
Khaper, N., et al. (2018). "Implications of disturbances in circadian rhythms for cardiovascular health: A new frontier in free radical biology." Free Radical Biology and Medicine 119: 85-92.	1	0	0	0	0	
Khapre, R. V., et al. (2010). "Circadian regulation of cell cycle: Molecular connections between aging and the circadian clock." Annals of Medicine 42(6): 404-415.	1	0	0	0	0	
Killien, M. G. (2004). "Nurses' health: work and family influences." Nursing Clinics of North America 39(1): 19-+.	1	1	0	0	0	
Kim, D.-S. and S.-K. Kang (2010). "Work-related Cerebro-Cardiovascular Diseases in Korea." Journal of Korean Medical Science 25: S105-S111.	1	0	0	0	0	
Kino, T. and G. P. Chrousos (2011). Circadian CLOCK-Mediated Regulation of Target-Tissue Sensitivity to Glucocorticoids: Implications for Cardiometabolic Diseases. Pediatric Adrenal Diseases. L. Ghizzoni, M. Cappa, G. Chrousos, S. Loche and M. Maghnie. 20: 116-+.	1	0	0	0	0	
Kittusamy, N. K. and B. Buchholz (2004). "Whole-body vibration and postural stress among operators of construction equipment: A literature review." Journal of Safety Research 35(3): 255-261.	1	0	0	0	0	
Kloss, J. D., et al. (2015). "Sleep, sleep disturbance, and fertility in women." Sleep Medicine Reviews 22: 78-87.	1	0	0	0	0	
Knardahl, S., et al. (2017). "The contribution from psychological, social, and organizational work factors to risk of disability retirement: a systematic review with meta-analyses." Bmc Public Health 17.	1	1	0	0	0	
Knutsson, A. and H. Boggild (2000). "Shiftwork and cardiovascular disease: review of disease mechanisms." Reviews on environmental health 15(4): 359-372.	1	1	0	0	0	

Knutsson, A. and A. Kempe (2014). "Shift work and diabetes - A systematic review." Chronobiology International 31(10): 1146-1151.	1	0	0	0	0	
Koch, C. E., et al. (2017). "Interaction between circadian rhythms and stress." Neurobiology of Stress 6: 57-67.	1	0	0	0	0	
Koli, T. and M. Filippidou (2017). "Audit on the Quality of Handovers of a Psychiatric Liaison Team in the UK: a Short Report." Psychiatria Danubina 29(Suppl 3): 536-540.	1	0	0	0	0	
Kondratov, R. V. (2007). "A role of the circadian system and circadian proteins in aging." Ageing Research Reviews 6(1): 12-27.	1	0	0	0	0	
Koo, D. L., et al. (2018). "Sleep Disturbances as a Risk Factor for Stroke." Journal of Stroke 20(1): 12-32.	1	0	0	0	0	
Kowalski-McGraw, M., et al. (2017). "Characterizing the Interrelationships of Prescription Opioid and Benzodiazepine Drugs With Worker Health and Workplace Hazards." Journal of Occupational and Environmental Medicine 59(11): 1114-1126.	1	0	0	0	0	
Kozlowska, L., et al. (2019). "HEALTH RISK IN TRANSPORT WORKERS PART II. DIETARY COMPOUNDS AS MODULATORS OF OCCUPATIONAL EXPOSURE TO CHEMICALS." International Journal of Occupational Medicine and Environmental Health 32(4): 441-464.	1	0	0	0	0	
Kristensen, T. S. (1989). "CARDIOVASCULAR-DISEASES AND THE WORK-ENVIRONMENT - A CRITICAL-REVIEW OF THE EPIDEMIOLOGIC LITERATURE ON NONCHEMICAL FACTORS." Scandinavian Journal of Work Environment & Health 15(3): 165-179.	1	1	0	0	0	
Kristensen, T. S. (1989). "Work environment and cardiovascular diseases. A short review of the literature." Journal of UOEH 11 Suppl: 120-133.	1	1	0	0	0	
Kubatka, P., et al. (2018). "Melatonin and breast cancer: Evidences from preclinical and human studies." Critical Reviews in Oncology Hematology 122: 133-143.	1	0	0	0	0	
Kumar, S., et al. (2019). "Environmental & occupational exposure & female reproductive dysfunction." Indian Journal of Medical Research 150(6): 532-545.	1	0	0	0	0	
Kushner, J. and T. Ruffin (2015). "Empowering a Healthy Practice Environment." Nursing Clinics of North America 50(1): 167-+.	1	0	0	0	0	
Kwiatkowski, F., et al. (2005). "Stress, cancer and circadian rhythm of melatonin." Pathologie Biologie 53(5): 269-272.	1	0	0	0	0	

Lawn, S., et al. (2020). "The effects of emergency medical service work on the psychological, physical, and social well-being of ambulance personnel: a systematic review of qualitative research." Bmc Psychiatry 20(1).	1	1	1	1	0	NO EVALUATION OF THE ASSOCIATION BETWEEN SW AND OUTCOME
Leatherwood, W. E. and J. L. Dragoo (2013). "Effect of airline travel on performance: a review of the literature." British Journal of Sports Medicine 47(9): 561-+.	1	0	0	0	0	
Ledda, C., et al. (2020). "Telomere Length as a Biomarker of Biological Aging in Shift Workers." Applied Sciences-Basel 10(8).	1	0	0	0	0	
Lee, J., et al. (2015). "Circadian control of -cell function and stress responses." Diabetes Obesity & Metabolism 17: 123-133.	1	0	0	0	0	
Lee, J., et al. (2018). "Untimely oxidative stress in beta-cells leads to diabetes - Role of circadian clock in beta-cell function." Free Radical Biology and Medicine 119: 69-74.	1	0	0	0	0	
Lees, T., et al. (2019). "A systematic review of the current evidence regarding interventions for anxiety, PTSD, sleepiness and fatigue in the law enforcement workplace." Industrial Health 57(6): 655-667.	1	0	0	0	0	
Leso, V., et al. (2020). "The Impact of Shift-Work and Night Shift-Work on Thyroid: A Systematic Review." International Journal of Environmental Research and Public Health 17(5).	1	0	0	0	0	
Leso, V., et al. (2021). "Shift or night shift work and dementia risk: a systematic review." European Review for Medical and Pharmacological Sciences 25(1): 222-232.	1	0	0	0	0	
Leung, J. M. and M. E. Martinez (2020). "Circadian Rhythms in Environmental Health Sciences." Current Environmental Health Reports 7(3): 272-281.	1	0	0	0	0	
Li, W., et al. (2019). "A meta-analysis of cohort studies including dose-response relationship between shift work and the risk of diabetes mellitus." European Journal of Epidemiology 34(11): 1013-1024.	1	0	0	0	0	
Lindbohm, M. L. (1999). "Women's reproductive health: Some recent developments in occupational epidemiology." American Journal of Industrial Medicine 36(1): 18-24.	1	0	0	0	0	
Linton, S. J., et al. (2015). "The effect of the work environment on future sleep disturbances: a systematic review." Sleep Medicine Reviews 23: 10-19.	1	0	0	0	0	

Lundstrom, T., et al. (2002). "Organizational and environmental factors that affect worker health and safety and patient outcomes." American Journal of Infection Control 30(2): 93-106.	1	1	0	0	0	
Madeira, S. G., et al. (2021). "The Impact of Different Types of Shift Work on Blood Pressure and Hypertension: A Systematic Review and Meta-Analysis." International Journal of Environmental Research and Public Health 18(13).	1	1	0	0	0	
Maiese, K. (2017). "Moving to the Rhythm with Clock (Circadian) Genes, Autophagy, mTOR, and SIRT1 in Degenerative Disease and Cancer." Current Neurovascular Research 14(3): 299-304.	1	0	0	0	0	
Manfredini, D. and F. Lobbezoo (2009). "Role of Psychosocial Factors in the Etiology of Bruxism." Journal of Orofacial Pain 23(2): 153-166.	1	0	0	0	0	
Mansel, J. K. and E. C. Carey (2014). "Nonpharmacologic Approach to Sleep Disorders." Cancer Journal 20(5): 345-351.	1	0	0	0	0	
Mansukhani, M. P., et al. (2012). "Sleep Deprivation in Resident Physicians, Work Hour Limitations, and Related Outcomes: A Systematic Review of the Literature." Postgraduate Medicine 124(4): 241-249.	1	0	0	0	0	
Martino, T. A. and M. E. Young (2015). "Influence of the Cardiomyocyte Circadian Clock on Cardiac Physiology and Pathophysiology." Journal of Biological Rhythms 30(3): 183-205.	1	0	0	0	0	
Matheson, A., et al. (2014). "The impact of shiftwork on health: a literature review." Journal of Clinical Nursing 23(23-24): 3309-3320.	1	1	1	1	0	NARRATIVE
McEwen, B. S. and I. N. Karatsoreos (2015). "Sleep Deprivation and Circadian Disruption Stress, Allostasis, and Allostatic Load." Sleep Medicine Clinics 10(1): 1-+.	1	0	0	0	0	
McVicar, A. (2003). "Workplace stress in nursing: a literature review." Journal of Advanced Nursing 44(6): 633-642.	1	1	1	1	0	NO EVALUATION OF THE ASSOCIATION BETWEEN SW AND OUTCOME
McVicar, A. (2016). "Scoping the common antecedents of job stress and job satisfaction for nurses (2000-2013) using the job demands-resources model of stress." Journal of Nursing Management 24(2): E112-E136.	1	1	0	0	0	
Meiliana, A., et al. (2015). "Chronodisruption and Obesity." Indonesian Biomedical Journal 7(3): 117-128.	1	0	0	0	0	
Metlaine, A., et al. (2005). "Socioeconomic impact of insomnia in working populations." Industrial Health 43(1): 11-19.	1	0	0	0	0	

Mikkelsen, S., et al. (2021). "Are depressive disorders caused by psychosocial stressors at work? A systematic review with metaanalysis." European Journal of Epidemiology 36(5): 479-496.	1	1	0	0	0	
Min, A., et al. (2019). "Work schedule characteristics and fatigue among rotating shift nurses in hospital setting: An integrative review." Journal of Nursing Management 27(5): 884-895.	1	1	0	0	0	
Mion, G. and S. Ricouard (2007). "Rest of safety: which stakes?" Annales Francaises D Anesthesie Et De Reanimation 26(7-8): 638-648.	1	0	0	0	0	
Mirza-Aghazadeh-Attari, M., et al. (2020). "Melatonin: An important anticancer agent in colorectal cancer." Journal of Cellular Physiology 235(2): 804-817.	1	0	0	0	0	
Moradkhani, F., et al. (2020). "Immunoregulatory role of melatonin in cancer." Journal of Cellular Physiology 235(2): 745-757.	1	0	0	0	0	
Munakata, M. (2018). "Clinical significance of stress-related increase in blood pressure: current evidence in office and out-of-office settings." Hypertension Research 41(8): 553-569.	1	0	0	0	0	
Munmun, F. and P. A. Witt-Enderby (2021). "Melatonin effects on bone: Implications for use as a therapy for managing bone loss." Journal of Pineal Research 71(1).	1	0	0	0	0	
Nader, N., et al. (2010). "Interactions of the circadian CLOCK system and the HPA axis." Trends in Endocrinology and Metabolism 21(5): 277-286.	1	0	0	0	0	
Nakata, A., et al. (2010). "Nurses, Smoking, and Immunity: A Review." Rehabilitation Nursing 35(5): 198-205.	1	0	0	0	0	
Narita, K. and E. Amiya "Social and environmental risks as contributors to the clinical course of heart failure." Heart Failure Reviews.	1	0	0	0	0	
Navara, K. J. and R. J. Nelson (2007). "The dark side of light at night: physiological, epidemiological, and ecological consequences." Journal of Pineal Research 43(3): 215-224.	1	0	0	0	0	
Naziroglu, M., et al. (2012). "Role of melatonin on electromagnetic radiation-induced oxidative stress and Ca2+ signaling molecular pathways in breast cancer." Journal of Receptors and Signal Transduction 32(6): 290-297.	1	0	0	0	0	
Nelson, D. (2007). "Prevention and treatment of sleep deprivation among emergency physicians." Pediatric Emergency Care 23(7): 498-503.	1	0	0	0	0	

Netter, P. and J. Hennig (1998). "The fibromyalgia syndrome as a manifestation of neuroticism?" Zeitschrift Fur Rheumatologie 57: 105-108.	1	0	0	0	0	
Nicholls, R., et al. (2017). "Barriers and facilitators to healthy eating for nurses in the workplace: an integrative review." Journal of Advanced Nursing 73(5): 1051-1065.	1	0	0	0	0	
Nicol, A.-M. and J. S. Botterill (2004). "On-call work and health: a review." Environmental health : a global access science source 3(1): 15-15.	1	0	0	0	0	
Nicolaides, N. C., et al. (2014). Circadian endocrine rhythms: the hypothalamic-pituitary-adrenal axis and its actions. Steroids in Neuroendocrine Immunology and Therapy of Rheumatic Diseases Ii. M. Cutolo, R. H. Straub, A. T. Masi and J. W. J. Bijlsma. 1318: 71-80.	1	0	0	0	0	
Nobre, B., et al. (2021). "Insomnia and circadian misalignment: an underexplored interaction towards cardiometabolic risk." Sleep Science 14(1): 55-63.	1	0	0	0	0	
Nurminen, T. (1995). "FEMALE NOISE EXPOSURE, SHIFT WORK, AND REPRODUCTION." Journal of Occupational and Environmental Medicine 37(8): 945-950.	1	0	0	0	0	
Otamas, A., et al. (2020). "Diabetes and atherothrombosis: The circadian rhythm and role of melatonin in vascular protection." Diabetes & Vascular Disease Research 17(3).	1	0	0	0	0	
Pagano, E. S., et al. (2017). "White Adipose Tissue and Circadian Rhythm Dysfunctions in Obesity: Pathogenesis and Available Therapies." Neuroendocrinology 104(4): 347-363.	1	0	0	0	0	
Palmer, K. T., et al. (2013). "Work activities and risk of prematurity, low birth weight and pre-eclampsia: an updated review with meta-analysis." Occupational and Environmental Medicine 70(4): 213-222.	1	0	0	0	0	
Pandalai, S. P., et al. (2013). "Conceptual heuristic models of the interrelationships between obesity and the occupational environment." Scandinavian Journal of Work Environment & Health 39(3): 221-232.	1	0	0	0	0	
Parkes, K. R. (1998). "Psychosocial aspects of stress, health and safety on North Sea installations." Scandinavian Journal of Work Environment & Health 24(5): 321-333.	1	0	0	0	0	

Patterson, P. D., et al. (2018). "Shorter Versus Longer Shift Durations to Mitigate Fatigue and Fatigue-Related Risks in Emergency Medical Services Personnel and Related Shift Workers: A Systematic Review." Prehospital Emergency Care 22: 28-36.	1	1	1	1	0	NO EVALUATION OF THE ASSOCIATION BETWEEN SW AND OUTCOME
Pejtersen, J. H., et al. (2015). "Update on Work-Related Psychosocial Factors and the Development of Ischemic Heart Disease A Systematic Review." Cardiology in Review 23(2): 94-98.	1	1	0	0	0	
Peter, R. and J. Siegrist (2000). "Psychosocial work environment and the risk of coronary heart disease." International Archives of Occupational and Environmental Health 73: S41-S45.	1	1	0	0	0	
Phillips, B., et al. (1991). "SHIFT WORK, SLEEP QUALITY, AND WORKER HEALTH - A STUDY OF POLICE OFFICERS." Southern Medical Journal 84(10): 1176-&.	1	0	0	0	0	
Pitsillou, E., et al. (2021). "The circadian machinery links metabolic disorders and depression: A review of pathways, proteins and potential pharmacological interventions." Life Sciences 265.	1	0	0	0	0	
Pluquet, O., et al. (2014). "Watching the clock: endoplasmic reticulum-mediated control of circadian rhythms in cancer." Annals of Medicine 46(4): 233-243.	1	0	0	0	0	
Poissonnet, C. M. and M. Veron (2000). "Health effects of work schedules in healthcare professions." Journal of Clinical Nursing 9(1): 13-23.	1	1	1	1	0	NO EVALUATION OF THE ASSOCIATION BETWEEN SW AND OUTCOME
Porkka-Heiskanen, T., et al. (2013). "Sleep, its regulation and possible mechanisms of sleep disturbances." Acta Physiologica 208(4): 311-328.	1	0	0	0	0	
Pougnet, R. and L. Pougnet (2021). "ANXIETY DISORDERS AND MOOD DISORDERS IN HOSPITAL DOCTORS: A LITERATURE REVIEW." Medycyna Pracy 72(2): 163-171.	1	0	0	0	0	
Pougnet, R., et al. (2020). "OCCUPATIONAL HEALTH OF MIDWIVES." Medycyna Pracy 71(4): 473-481.	1	0	0	0	0	
Pousa, P. C. P. and S. R. de Lucca (2021). "Psychosocial factors in nursing work and occupational risks: a systematic review." Revista Brasileira De Enfermagem 74.	1	1	0	0	0	
Pryce, C. (2016). "Impact of shift work on critical care nurses." The Canadian journal of critical care nursing 27(4): 17-21.	1	1	1	1	0	NARRATIVE

Puttonen, S., et al. (2010). "Shift work and cardiovascular disease - pathways from circadian stress to morbidity." Scandinavian Journal of Work Environment & Health 36(2): 96-108.	1	1	1	1	0	NARRATIVE
Rakshit, K., et al. (2014). "Does Disruption of Circadian Rhythms Contribute to Beta-Cell Failure in Type 2 Diabetes?" Current Diabetes Reports 14(4).	1	0	0	0	0	
Redeker, N. S., et al. (2019). "Workplace Interventions to Promote Sleep Health and an Alert, Healthy Workforce." Journal of Clinical Sleep Medicine 15(4): 649-657.	1	0	0	0	0	
Reiter, R. J., et al. (2014). "Melatonin and stable circadian rhythms optimize maternal, placental and fetal physiology." Human Reproduction Update 20(2): 293-307.	1	0	0	0	0	
Remi, J. (2015). "Humans Entrain to Sunlight - Impact of Social Jet Lag on Disease and Implications for Critical Illness." Current Pharmaceutical Design 21(24): 3431-3437.	1	0	0	0	0	
Reynolds, A. C., et al. (2017). "The shift work and health research agenda: Considering changes in gut microbiota as a pathway linking shift work, sleep loss and circadian misalignment, and metabolic disease." Sleep Medicine Reviews 34: 3-9.	1	0	0	0	0	
Richter, K., et al. (2021). "Shiftwork and Alcohol Consumption: A Systematic Review of the Literature." European Addiction Research 27(1): 9-15.	1	1	0	0	0	
Rogers, B. (1997). "Health hazards in nursing and health care: An overview." American Journal of Infection Control 25(3): 248-261.	1	0	0	0	0	
Rohatgi, S., et al. (2015). "After-Hours Radiology: Challenges and Strategies for the Radiologist." American Journal of Roentgenology 205(5): 956-961.	1	0	0	0	0	
Rosa, D., et al. (2019). "Systematic review of shift work and nurses' health." Occupational Medicine-Oxford 69(4): 237-243.	1	1	1	1	0	NO EVALUATION OF THE ASSOCIATION BETWEEN SW AND OUTCOME
Roth, T. and T. A. Roehrs (1996). "Etiologies and sequelae of excessive daytime sleepiness." Clinical Therapeutics 18(4): 562-576.	1	0	0	0	0	
Rugulies, R., et al. (2019). "WHO/ILO work-related burden of disease and injury: Protocol for systematic reviews of exposure to long working hours and of the effect of exposure to long working hours on depression." Environment International 125: 515-528.	1	0	0	0	0	

Rutenfranz, J., et al. (1977). "BIOMEDICAL AND PSYCHOSOCIAL-ASPECTS OF SHIFT WORK - REVIEW." Scandinavian Journal of Work Environment & Health 3(4): 165-182.	1	1	0	0	0	
Sakuraya, A., et al. (2017). "Work-related psychosocial factors and onset of metabolic syndrome among workers: a systematic review and meta-analysis protocol." Bmj Open 7(6).	1	1	0	0	0	
Scott, C. and A. M. Johnstone (2012). "Stress and Eating Behaviour: Implications for Obesity." Obesity Facts 5(2): 277-287.	1	0	0	0	0	
Sharma, A., et al. (2021). "Circadian Rhythm Disruption and Alzheimer's Disease: The Dynamics of a Vicious Cycle." Current Neuropharmacology 19(2): 248-264.	1	0	0	0	0	
Shaw, E. and G. H. Tofler (2009). "Circadian rhythm and cardiovascular disease." Current Atherosclerosis Reports 11(4): 289-295.	1	0	0	0	0	
Simko, F., et al. (2013). "Experimental models of melatonin-deficient hypertension." Frontiers in Bioscience-Landmark 18: 616-625.	1	0	0	0	0	
Smith, E. C., et al. (2019). "Exploring the Physical and Mental Health Challenges Associated with Emergency Service Call-Taking and Dispatching: A Review of the Literature." Prehospital and Disaster Medicine 34(6): 619-624.	1	1	0	0	0	
Smith, L., et al. (1998). "Work shift duration: a review comparing eight hour and 12 hour shift systems." Occupational and Environmental Medicine 55(4): 217-229.	1	1	1	1	0	NO EVALUATION OF THE ASSOCIATION BETWEEN SW AND OUTCOME
Sofianopoulos, S., et al. (2012). "Paramedics and the effects of shift work on sleep: a literature review." Emergency Medicine Journal 29(2): 152-155.	1	0	0	0	0	
Soteriades, E. S., et al. (2011). "Cardiovascular Disease in US Firefighters A Systematic Review." Cardiology in Review 19(4): 202-215.	1	0	0	0	0	
Spurgeon, A., et al. (1997). "Health and safety problems associated with long working hours: A review of the current position." Occupational and Environmental Medicine 54(6): 367-375.	1	0	0	0	0	
Srinivasan, V., et al. (2011). "Melatonin, immune function and cancer." Recent patents on endocrine, metabolic & immune drug discovery 5(2): 109-123.	1	0	0	0	0	
Srinivasan, V., et al. (2008). "Melatonin, environmental light, and breast cancer." Breast Cancer Research and Treatment 108(3): 339-350.	1	0	0	0	0	

Staufenbiel, S. M., et al. (2013). "Hair cortisol, stress exposure, and mental health in humans: A systematic review." Psychoneuroendocrinology 38(8): 1220-1235.	1	0	0	0	0	
Stec, N., et al. (2018). "A Systematic Review of Fatigue in Radiology: Is It a Problem?" American Journal of Roentgenology 210(4): 799-806.	1	0	0	0	0	
Steenland, K. (1996). "Epidemiology of occupation and coronary heart disease: Research agenda." American Journal of Industrial Medicine 30(4): 495-499.	1	0	0	0	0	
Storr, C. L., et al. (2000). "Similarities of substance use between medical and nursing specialties." Substance Use & Misuse 35(10): 1443-1469.	1	0	0	0	0	
Strohmaier, S., et al. (2018). "A Review of Data of Findings on Night Shift Work and the Development of DM and CVD Events: a Synthesis of the Proposed Molecular Mechanisms." Current Diabetes Reports 18(12).	1	0	0	0	0	
Tahara, Y. and S. Shibata (2018). "Entrainment of the mouse circadian clock: Effects of stress, exercise, and nutrition." Free Radical Biology and Medicine 119: 129-138.	1	0	0	0	0	
Tamashiro, K. L., et al. (2011). "Chronic stress, metabolism, and metabolic syndrome." Stress-the International Journal on the Biology of Stress 14(5): 468-474.	1	0	0	0	0	
Tan, D.-X., et al. (2015). "Melatonin as a Potent and Inducible Endogenous Antioxidant: Synthesis and Metabolism." Molecules 20(10): 18886-18906.	1	0	0	0	0	
Taouk, Y., et al. (2020). "Psychosocial work stressors and risk of all-cause and coronary heart disease mortality: A systematic review and meta-analysis." Scandinavian Journal of Work Environment & Health 46(1): 19-31.	1	1	0	0	0	
Taylor, E., et al. (1997). "Models of shiftwork and health: An examination of the influence of stress on shiftwork theory." Human Factors 39(1): 67-82.	1	1	0	0	0	
Theorell, T., et al. (2016). "A systematic review of studies in the contributions of the work environment to ischaemic heart disease development." European Journal of Public Health 26(3): 470-477.	1	0	0	0	0	
Thorpy, M. J., et al. (2007). "Patient-management strategies." American Journal of Managed Care 13(6): S140-S147.	1	0	0	0	0	
Togo, F. and M. Takahashi (2009). "Heart Rate Variability in Occupational Health -A Systematic Review." Industrial Health 47(6): 589-602.	1	0	0	0	0	

Torquati, L., et al. (2018). "Shift work and the risk of cardiovascular disease. A systematic review and meta-analysis including dose-response relationship." Scandinavian Journal of Work Environment & Health 44(3): 229-238.	1	1	0	0	0	
Touitou, Y., et al. (2017). "Association between light at night, melatonin secretion, sleep deprivation, and the internal clock: Health impacts and mechanisms of circadian disruption." Life Sciences 173: 94-106.	1	0	0	0	0	
Valenzuela, F. J., et al. (2015). "Circadian System and Melatonin Hormone: Risk Factors for Complications during Pregnancy." Obstetrics and Gynecology International 2015.	1	0	0	0	0	
van Mark, A., et al. (2006). "Shift work and pathological conditions." Journal of Occupational Medicine and Toxicology 1.	1	1	0	0	0	
Van Reeth, O., et al. (2000). "Interactions between stress and sleep: from basic research to clinical situations." Sleep Medicine Reviews 4(2): 201-219.	1	0	0	0	0	
Varoni, E. M., et al. (2016). "The Impact of Melatonin in Research." Molecules 21(2).	1	0	0	0	0	
Virshup, D. M., et al. (2007). "Reversible protein phosphorylation regulates circadian rhythms." Cold Spring Harbor symposia on quantitative biology 72: 413-420.	1	0	0	0	0	
Vogel, M., et al. (2012). "The effects of shift work on physical and mental health." Journal of Neural Transmission 119(10): 1121-1132.	1	1	1	1	0	NARRATIVE
Wajid, F., et al. (2020). "Therapeutic potential of melatonin as a chronobiotic and cytoprotective agent in diabetes mellitus." Journal of Diabetes and Metabolic Disorders 19(2): 1797-1825.	1	0	0	0	0	
Waldman, H. S., et al. (2020). "Time-restricted feeding for the prevention of cardiometabolic diseases in high-stress occupations: a mechanistic review." Nutrition Reviews 78(6): 459-464.	1	1	0	0	0	
Watanabe, K., et al. (2018). "Work-related psychosocial factors and metabolic syndrome onset among workers: a systematic review and meta-analysis." Obesity Reviews 19(11): 1557-1568.	1	1	0	0	0	
Weibel, L., et al. (1999). "Modifications in biological rhythms in night-shift workers." Presse Medicale 28(5): 252-258.	1	0	0	0	0	
Wester, V. L. and E. F. C. van Rossum (2015). "Clinical applications of cortisol measurements in hair." European Journal of Endocrinology 173(4): M1-M10.	1	0	0	0	0	

Williams, S. G., et al. (2014). "The Impact of Sleep on Soldier Performance." Current Psychiatry Reports 16(8).	1	0	0	0	0	
Willis, S. K., et al. (2019). "Sleep and female reproduction." Current Opinion in Obstetrics & Gynecology 31(4): 222-227.	1	0	0	0	0	
Wolk, R. and V. K. Somers (2007). "Sleep and the metabolic syndrome." Experimental Physiology 92(1): 67-78.	1	0	0	0	0	
Wong, M. L. and A. S. Chung (2020). "Strategies for Provider Well-Being in the Emergency Department." Emergency Medicine Clinics of North America 38(3): 729-+.	1	0	0	0	0	
Wosu, A. C., et al. (2013). "Correlates of cortisol in human hair: implications for epidemiologic studies on health effects of chronic stress." Annals of Epidemiology 23(12): 797-811.	1	0	0	0	0	
Yamada, Y., et al. (2002). "Prevention of weight gain and obesity in occupational populations: A new target of health promotion services at worksites." Journal of Occupational Health 44(6): 373-384.	1	0	0	0	0	
Yasutake, K., et al. (2014). "Dietary habits and behaviors associated with nonalcoholic fatty liver disease." World Journal of Gastroenterology 20(7): 1756-1767.	1	0	0	0	0	
Yu, E., et al. (2016). "Diet, Lifestyle, Biomarkers, Genetic Factors, and Risk of Cardiovascular Disease in the Nurses' Health Studies." American Journal of Public Health 106(9): 1616-1623.	1	0	0	0	0	
Zelinski, E. L., et al. (2014). "The trouble with circadian clock dysfunction: Multiple deleterious effects on the brain and body." Neuroscience and Biobehavioral Reviews 40: 80-101.	1	0	0	0	0	
Zhang, T.-W., et al. (2020). "The circadian rhythm in intervertebral disc degeneration: an autophagy connection." Experimental and Molecular Medicine 52(1): 31-40.	1	0	0	0	0	
Zhu, L., et al. (2014). "Research progress on the central mechanism underlying regulation of visceral biological rhythm by per2." Molecular Medicine Reports 10(5): 2241-2248.	1	0	0	0	0	
Zimmerman, F. H. (2012). "Cardiovascular Disease and Risk Factors in Law Enforcement Personnel: A Comprehensive Review." Cardiology in Review 20(4): 159-166.	1	0	0	0	0	

Supplemental figure A. Flowcharts of the selection process using PRISMA guidelines

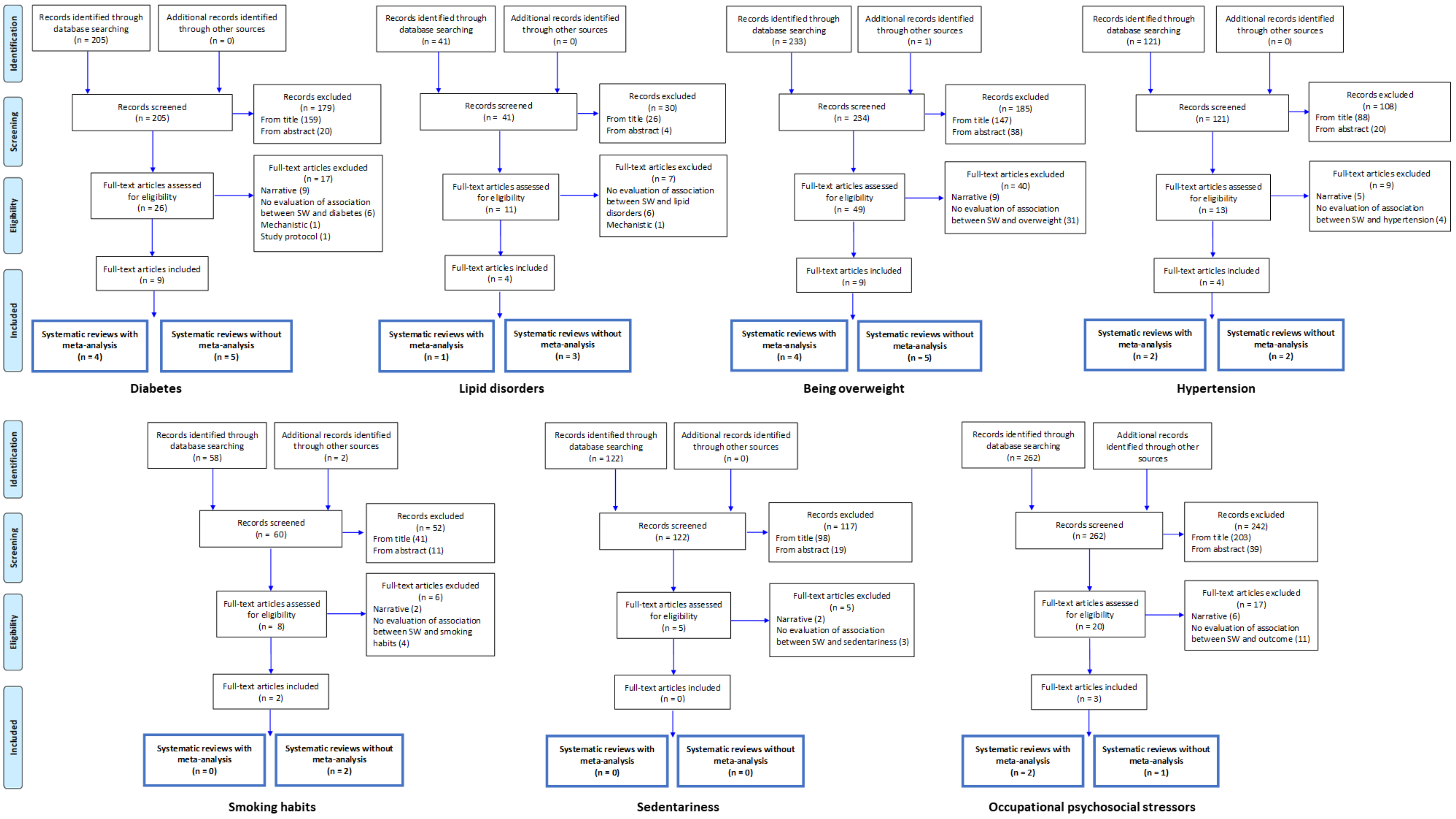

Supplemental table C. Characteristics of systematic reviews focused on the link between shift work and diabetes

First Author Year publication, Country	Characteristics of population		Setting and context	Database searching		Studies included in each review				Quality of the primary studies		Shift work		Assessment of outcomes (n)
	Gender, age [mean, range] (n)	N participants [min-max]		Source	Date range	Publication date range	n	Type (n)	Countries of origin	Instrument used	Rating (n)	Type (n)	Exposure duration (n)	
**Wang<br>2011,UK**	♀, ♂<br>Age MD	77,054<br>[2,167-336,840]	Industry (5)<br>Health care (1)	Pubmed	Until<br>12/2009	1983-2007	6	Prospective (3)<br>Retrospective (1)<br>Cross-sectional (2)	America (1)<br>EU (1)<br>Asia (4)	No	No	Rotating (4)<br>Unspecified (2)	Yes (2)	Type 2 diabetes (1) Diabetes mellitus (5)<br>Diabetes death certificate (1)<br>FPG or patient under treatment (1)<br>MD (4)
**Esquirol<br>2011,France**	♀, ♂ and both<br>Mean age range<br>[28.5-62.3] yo (21)<br>Age range<br>[29-60] yo (2)	146,940<br>[147-62,574]	Industry (13)<br>Health care (4)<br>Heterogeneous (7)	PubMed<br>Cochrane library<br>Embase	2000-2010	2001-2010	24	Prospective (12)<br>Retrospective (1)<br>Cross-sectional (11)	Asia (12)<br>EU (10)<br>America (2)	No	No	Permanent Night (3)<br>Rotating (21)<br>Unspecified (2)<br>Evening (1)	Yes (4)	Type 2 Diabetes (1); diabetes mellitus (23)<br>Glycaemia (11), HbA1c (8), FPG (2)<br>OGTT (2), RPG (1), Self-reported (1)<br>Unknown (2)
**Knutsson<br>2014,Sweden**	♀, ♂<br>Age range<br>[14-60] yo (3)<br>Mean age<br>[34.3-53.7] yo (2)	193,309<br>[2,194-177,184]	Industry (4)<br>Health care (1)	Pubmed<br>Noishtic2<br>Science Direct	Until<br>11/2012	1999-2011	5	Prospective (4)<br>Restrospective (1)	EU (1)<br>America (1)<br>Asia (3)	No	No	Rotating (5)	Yes (2)	Type 2 diabetes (5) Type 1 diabetes (1)<br>OGTT (1), Death certificate (1)<br>Self-reported (1), HbA1c (2)
**Gan<br>2015,China<br>Meta-analysis**	♀, ♂ and both<br>Age range [<15-70] (9)<br>Mean age<br>[34.3-63.6] yo (6)	226,652<br>[475-107,915]	Industry (8)<br>Health care (1)<br>Heterogeneous (2)	PubMed<br>Embase<br>Web of Science<br>ProQuest<br>Dissertation<br>Theses	Until<br>04/2014	1983-2013	11	Prospective (6)<br>Retrospective (1)<br>Cross-sectional (4)	EU (3)<br>America (1)<br>Asia (7)	NOS<br>AHRQ	Cohort studies<br>High (7)<br>Cross-sectional<br>High (1)<br>Moderate (2)<br>Low(1)	Night shift (3)<br>Rotating (3x8) (4)<br>Mixed rotating<br>(2x8/3x8)(2)<br>Unspecified (2)	No	Type 2 diabetes (4); Diabetes mellitus (7)<br>Diabetes death certificate (1)<br>Self-reported or medical/register report (10), FPG (2), HbA1c (2)
**Proper<br>2016,Netherlands**	♂, ♀ and both<br>Age range<br>[19-62] yo (5)<br>Mean age range<br>[32.8-63.6] yo (3)	66,862<br>[387-26,382]	Industry (5)<br>Heterogeneous (4)	Embase<br>Biosis<br>Previews<br>SciSearch	1995-<br>03/2015	2005-2015	9	Retrospective (1)<br>Prospective (8)	EU (3)<br>Asia (6)	Personalized checklist	Low (5)<br>High (4)	Permanent Night (1)<br>Rotating (8)	Yes (2)	Type 2 diabetes (2) Diabetes mellitus (7)<br>Glycaemia (4), OGTT (1), RPG (1), FPG (1), HbA1c (4)
**Anothaisintawee<br>2016,Thailand<br>Meta-analysis**	♀, ♂<br>Mean age<br>[34.3-5.4] yo (10)	262,294<br>[276-107,915]	Industry (5)<br>Health care (1)<br>Heterogeneous (4)	Medline, Scopus	Until<br>11/2013	2005-2013	11	Prospective (11)	EU (2)<br>America (4)<br>Asia (5)	modified NOS<br>(5 criteria of bias risk)	Low bias risk (6)<br>Moderate bias risk (4)<br>High bias risk (1)	Rotating (5)<br>Unspecified (6)	Yes (1)	Type 2 Diabetes (4); Diabetes mellitus (7)<br>FPG (4), HbA1c (3), OGTT (2), Self reported (4)<br>Medical diagnosis or treatment (6)
**Li<br>2019,China<br>Meta-analysis**	♀, ♂ and both<br>Age range<br>[<15-69] yo (6) or<br>Mean age<br>[35.1-42.3] yo (3)	244,266<br>[1,529-88,086]	Industry (6)<br>Health care (3)<br>Heterogeneous (3)	Pubmed, Web of Science	Until<br>08/2019	1999-2018	12	Prospective (11)<br>Retrospective (1)	EU (6)<br>America (2)<br>Asia (4)	NOS	High (12)	Permanent night (4)<br>Evening (4)<br>Rotating (5)<br>Unspecified (3)	Yes (2)	Type 2 diabetes (2); Diabetes mellitus (10)<br>Diabetes death certificate<br>Self-reported or medical/register report, HbA1c
**Rosa<br>2019,Italy**	♀<br>Age MD	28,731	Health care (1)	PubMed, Cinahl<br>Scopus, Embase	2005-2016	2016	1	Prospective (1)	EU (1)	Checklist by Dixon-Woods	High (1)	Night shift (1)	Yes (1)	Type 2 diabetes<br>Register (1)
**Gao<br>2020,China<br>Meta-analysis**	♀, ♂ and both<br>Age range<br>[18-78] yo (17)<br>Mean age<br>[38.1-63.6] yo (5)	639,880<br>[373-1,778,721]	Industry (3)<br>Health care (4)<br>Administration (3)<br>Heterogeneous (11)	Pubmed, Embase,<br>Web of Science	Until<br>06/2019	1999-2018	21	Cross-sectional (9)<br>Prospective (12)	EU (8)<br>America (6)<br>Asia (7)	NOS<br>AHRQ	Prospective studies<br>High (10)/Moderate (2)<br>Cross-sectional<br>High (3)<br>Moderate (6)	Night shift(10)<br>Rotating (4)<br>Evening (2)<br>Unspecified (5)	Yes (6)	Type 2 Diabetes (21)<br>FPG, HbA1c, OGTT, RPG

N: number of participants; (n): number of studies concerned; yo: years old; FPG: Fasting plasma glucose; OGTT: Oral Glucose Tolerance Test; RPG: Random plasma glucose; HbA1c: glycated hemoglobin; MD: missing data

Supplemental table D. Characteristics of systematic reviews focused on the link between shift work and lipid disorders

First Author Year publication, Country	Characteristics of population		Setting and context	Database searching		Studies included in each review				Quality of the primary studies		Shift work		Assessment of outcomes (n)
	Gender, age [mean, range] (n)	N participants [min-max]		Source	Date range	Publication date range	n	Type (n)	Countries of origin	Instrument used	Rating (n)	Type (n)	Exposure duration (n)	
**Boggild 1999, Nordic countries**	MD	12,578	MD	Medline, NIOSH-TIC	MD	1976-1996	16	Cross-sectional (10)<br>Prospective (6)	MD	Principles of Kristensen	Cross-sectional studies: low (5)/moderate (2)/ High (3)<br>Prospective studies: Low (1)/ Moderate (1)/ High (4)	Unspecified (16)	No	TC (16), TG (12) measurements, HDL-C/LDL-C (3)
**Esquirol 2011, France**	♂, ♀ and both<br>Age range [24-60] yo (2)<br>Mean range [28.5-62.3] yo (18)	81,177 [70-9,857]	Industry (13)<br>Health care (2)<br>Administration (1)<br>Heterogeneous (7)	PubMed, Cochrane library, Embase	01/2000 - 12/2010	2001-2010	23	Cross-sectional (11)<br>Prospective (11)<br>Retrospective (1)	EU (11)<br>South America (1)<br>Asia (11)	No	No	Permanent night (5)<br>Rotating (18)<br>Unspecified (2)	Yes (1)	TC (13), TG (6), HDL-C (8), LDL-C (6) measurements, HighTG (6), LowHDL-C (6), HighTC (3), self-reported highTC (1)
**Proper 2016, Netherlands**	♂, ♀ and both<br>Age range [19-62] yo (5)<br>Mean range [26.8-63.6] yo (6)	70,307 [387-26,382]	Industry (8)<br>Health care (1)<br>Heterogeneous (4)	Medline, Embase, Biosis previews, SciSearch	1995 - 03/2015	2007-2015	12	Prospective (11)<br>Retrospective (1)	EU (4)<br>Asia (8)	Personalized checklist	High (6)<br>Low (6)	Permanent night (1)<br>Rotating (8)<br>Unspecified (3)	Yes (1)	TC (7), TG (4), HDL-C (3), LDL-C (1) measurements, HighTG (3), LowHDL-C (3), HighTC (1), LDL-C/HDL-C (1)
**Dutheil 2020, France, Meta-analysis**	♂, ♀ and both<br>Mean range [30-62] yo	67,514 [118-37,014]	Industry (30)<br>Health care (14)<br>Administration (3)<br>Unspecified (19)	PubMed, Embase, Cochrane library, ScienceDirect	Until 02/2020	2001-2019	66	Cross-sectional (41)<br>Prospective & Retrospective (24)<br>Case-control (1)	EU (23)<br>Africa (2)<br>America (8)<br>Asia (33)	NOS<br>STROBE		Permanent night<br>Rotating<br>Unspecified	No	SMD or high level of TC , LDL-C, low level of HDL-C

N: number of participants; (n): number of studies concerned; yo: years old; HDL-C: High-Density Lipoprotein Cholesterol; LDL-C: Low-Density Lipoprotein Cholesterol; TC: Total Cholesterol; TG: Total Triglycerides; SMD: standardized mean difference; MD: missing data.

Supplemental table E. Characteristics of systematic reviews focused on the link between shift work and being overweight

First Author, Year publication, Country	Characteristics of population		Setting and context	Database searching		Studies included in each review				Quality of the primary studies		Shift work		Assessment of outcomes (n)
	Gender, age [mean, range] (n)	N participants [min-max]		Source	Date range	Publication date range	n	Type (n)	Countries of origin	Instrument used	Rating (n)	Type (n)	Exposure duration (n)	
**Antunes 2010, Brazil**	♂ and both<br>Age MD	19,808<br>[299-7,965]	Industry (4)<br>Heterogeneous (3)<br>MD (2)	Medline<br>Cochrane	1960-2008	1997-2008	9	Prospective (1)<br>Cross-sectional (7)<br>Retrospective (1)	EU (5)<br>America (1)<br>Asia (3)	No	No	Night shift (1)<br>Rotating (3)<br>Unspecified (5)	yes (3)	BMI (8); BMI ≥25 kg/m² (1)<br>WHR (5)
**Esquirol 2011, France**	♀, ♂ and both<br>Mean age<br>[29-60] yo (16)	66,394<br>[85-27,485]	Industry (14)<br>Health care (4)<br>Heterogeneous (5)	PubMed,<br>Cochrane library,<br>Embase	2000-2010	2000-2010	22	Prospective (9)<br>Cross-sectional (13)	EU (10)<br>America (3)<br>Asia (9)	No	No	Permanent night (3)<br>Rotating (18)<br>Unspecified (1)	Yes (5)	Weight change (2)<br>BMI (15); BMI ≥ 25 or ≥ 30 kg/m² (4)<br>WC (3); WC ≥ 80 or ≥ 94 cm (2)<br>WHR (6); WHR > 0.9 (1)
**Van Drongelen 2011,The Netherlands**	♀, ♂ and both<br>Age MD (8)	11,537<br>[58-7,254]	Industry (5)<br>Health care (4)<br>Heterogeneous (1)	Medline,<br>Embase,<br>Cochrane library,<br>PsycINFO	Until 06/2010	1986-2008	8	Prospective (6)<br>Retrospective (2)	EU (5)<br>America (1)<br>Asia (2)	Checklists of Hayden and Van der Windt	High quality (5)<br>Low quality (3)	Permanent night (2)<br>Rotating (5)<br>Unspecified (2)	No	BMI change (4)<br>Weight change (4)<br>WC change (2)
**Amani 2013, Iran**	Age range<br>[21-64] yo (4)<br>Mean age<br>[30-42] yo (3)<br>Age MD (2)	33,533<br>[299-27,485]	Industry (5)<br>Health care (2)<br>Heterogeneous (2)	Medline,<br>PubMed,<br>Cinhal	1990-2011	1996-2006	9	Cohort (2)<br>Cross-sectional (7)	EU (6)<br>Asia (3)	No	No	Night shift (1)<br>Rotating (4)<br>Unspecified (4)	No	BMI ≥ 30 or ≥ 25 or ≥ 27 kg/m² (5); Weight change (1); BMI (4)
**Proper 2016, Netherlands**	♀, ♂ and both<br>Age range [18-70] yo (8)<br>Mean age [26-63.6] yo (8)	203,657<br>[85-107,663]	Industry (8)<br>Health care (7)<br>Heterogeneous (6)	Medline,<br>Embase, Biosis previews,<br>SciSearch	1995-03/2015	1999-2015	19	Prospective (17)<br>Retrospective (1)<br>Unspecified (1)	EU (8)<br>America (2)<br>Asia (9)	Personalized checklist	High (4)<br>Low (15)	Night shift (5)<br>Rotating (12)<br>Unspecified (5)	MD	Weight change (3)<br>BMI (10); BMI ≥25 or ≥ 30 kg/m² (5)<br>WC (2); WC ≥ 80 or ≥ 94 cm (2)<br>WHR (2)
**Liu 2018, China Meta-analysis**	♀, ♂ and both<br>Age MD (27)	311,334<br>[150-54,724]	Health care (6)<br>Heterogeneous (16)<br>MD (5)	Pubmed,<br>Embase	Until 12/2017	1999-2017	27	Cohort (7)<br>Cross-sectional (19)<br>Case-control (1)	EU (8)<br>America (9)<br>Asia (8)<br>Multi-countries (2)	NOS	High (24)<br>Moderate (3)	Night shift (5)<br>Rotating shift (18)<br>Unspecified (4)	yes (3)	BMI ≥ 25 or ≥ 23 kg/m² (11); WC ≥ 94 cm (1)<br>BMI ≥ 25 or ≥ 30 kg/m² (23); total fat % (1); ICD-10 (1)
**Saulle 2018, Italy Meta-analysis**	♀ and both<br>Age MD (7)	22,082<br>[353-9,989]	Health care (7)	Medline<br>Scopus	MD	2006-2016	7	Cross-sectional (6)<br>Prospective (1)	EU (3)<br>America (3)<br>Asia (1)	NOS	Cross-sectional<br>Moderate (6)<br>Prospective<br>High (1)	Unspecified (7)	yes (2)	BMI> 25 or > 30 kg/m² (4)<br>BMI (2)<br>WC (1)
**Sun 2018, China Meta-analysis**	♀, ♂ and both<br>Age range [30-55] yo (20)	269,901<br>[57-54,724]	Industry (10)<br>Health care (11)<br>Heterogeneous (7)	PubMed	Until 03/2017	1996-2016	28	Prospective (6)<br>Cross-sectional (22)	EU (9)<br>America (8)<br>Asia (10)<br>Multi-countries (1)	STROBE	HIgh (15)<br>Moderate (13)	Night shift (15)<br>Rotating (16)<br>Unspecified (4)	yes (4)	Weight/BMI gain (2)<br>BMI ≥ 25 or 25-29.9 or ≥30 kg/m² (28)<br>WC or WHR (9)
**Zhang 2020, China Meta-analysis**	♀ and both<br>Mean age<br>[33-55] yo	74,651<br>[200-54,724]	Health care (11)	PubMed,<br>Medline,<br>Cochrane library,<br>Embase, Web of Science	Until 04/2020	2012-2016	11	Cohort (2)<br>Cross-sectional (9)	EU (2)<br>America (4)<br>Asia (5)	ROBINS-I	Low or moderate risk of bias (11)	Night shift (5)<br>Shift work (6)	No	BMI ≥25 or ≥30 or >30 kg/m2 (10)<br>WC ≥80 cm or WC ≥88 cm (2)

N: number of participants; (n): number of studies concerned; yo: years old; BMI: Body Mass Index; WHR: Waist Hip Ratio; WC: Waist circumference; MD: missing data.

Supplemental table F. Characteristics of systematic reviews focused on the link between shift work and hypertension

First Author, Year publication, Country	Characteristics of population		Setting and context	Database searching		Studies included in each review				Quality of the primary studies		Shift work		Assessment of outcomes (n)
	Gender, age [mean, range] (n)	N participants [min-max]		Source	Date range	Publication date range	n	Type (n)	Countries of origin	Instrument used	Rating (n)	Type (n)	Exposure duration (n)	
**Esquirol 2011, France**	♀, ♂ and both<br>Age range [24-60] yo (2)<br>Mean range [25.4-62.3] yo (28)	105,635 [12-27,485]	Industry (17)<br>Health care (7)<br>Heterogeneous (10)	PubMed, Cochrane library, Embase	01/2000-12/2010	2000-2010	34	Cross-sectional (20)<br>Prospective (14)	EU (15)<br>South america (3)<br>Asia (16)	No	No	Permanent night (5)<br>Rotating (33)<br>Evening (2)	Yes (2)	HTN (13)<br>BP measures (14)<br>24-h Ambulatory BP (5)<br>HTN history (2)
**Proper 2016, Netherlands**	♀, ♂ and both<br>Age range [18-62] yo (7)<br>Mean age range [24.1-63.6] yo (11)	201,937 [233-95,652]	Industry (12)<br>Health care (2)<br>Heterogeneous (5)	Medline, Embase, Biosis previews, SciSearch	1995-03/2015	1999-2015	19	Prospective (17)<br>Retrospective (2)	EU (5)<br>America (1)<br>Asie (13)	Personalized checklist	High (11)<br>Low (8)	Permanent night (1)<br>Rotating (13)<br>Unspecified (5)	Yes (2)	HTN (11)<br>BP measures (7)<br>Self-reported (3)<br>Register (1)
**Manohar 2017, USA Meta-analysis**	♀, ♂ and both<br>Age range [18-68] yo (19)	394,793 [98-95,652]	Industry (16)<br>Health care (3)<br>Heterogeneous (8)	Medline, Embase, Cochrane Database of Systematic Reviews, Cochrane Central Register of Controlled Trials	Until 08/2016	1999-2015	27	Cohort (9)<br>Cross-sectional (18)	EU (5)<br>Asia (17)<br>America (5)	NOS	Cross-sectional studies: 7-10 /10<br>Cohort studies : 5-9/9	Rotating (18)<br>Permanent night (4)<br>Irregular (2)<br>Unspecified (4)	No	HTN (18)<br>Self-reported BP (7)<br>BP measures (1)<br>MD (1)
**Gamboa Madeira 2021, Portugal Meta-analysis**	♀, ♂ and both<br>Mean age range [22.3-64.2] yo (37)<br>Age range [20-62] yo (6)<br>MD age (3)	117,252 [47-26,463]	Industry (30)<br>Health care (4)<br>Heterogeneous (11)	Medline, Embase, Cochrane Library electronic database	Until 02/2019	1986-2015	45	Cross-sectional (42)<br>Prospective (1)<br>Retrospective (2)	Asia (21)<br>EU (13)<br>America (9)<br>Africa (2)	NOS	Mean NOS score = 5.6;<br>Median NOS score = 5<br>Low (11)	Permanent night (14)<br>Rotating with nights (30) without nights (4)<br>Unspecified (8)	No	HTN (14)<br>BP measures (41)

N: number of participants; (n): number of studies concerned; yo: years old; HTN: Hypertension; SBP and DBP: Systolic and Diastolic Blood Pressure; MD: Missing Data

Supplemental table G. Characteristics of systematic reviews focused on the link between shift work and smoking habits

First Author, Year publication, Country	Characteristics of population		Setting and context	Database searching		Studies included in each review				Quality of the primary studies		Shift work		Assessment of outcomes (n)
	Gender, age [mean, range] (n)	N participants [min-max]		Source	Date range	Publication date range	n	Type (n)	Countries of origin	Instrument used	Rating (n)	Type (n)	Exposure duration (n)	
**Boggild 1999, Nordic countries**	MD	25,097	MD	Medline<br>NIOSH-TIC	MD	1976-1998	16	Cross-sectional (12)<br>Prospective (4)	MD	Principles of Kristensen	Cross-sectional studies:<br>Low (5)<br>Moderate (4)<br>High (5)<br>Prospective studies:<br>Moderate (1)<br>High (1)	Unspecified (16)	No	Smokers, % (14);<br>Cigarettes/day (2)
**Zhao 2008 Australia**	♀, ♂and both<br>Age range [35-60] yo (1)<br>Mean age [24.1-43.1] yo (5)	7,244<br>[36-5,420]	Industry (1)<br>Health care (3)<br>Heterogeneous (3)	Cinahl<br>Nursing/Academic Edition,<br>Medline, PubMed,<br>PsysInfo,<br>Proquest health<br>Cochrane library	Until 12/ 2006	1997-2004	7	Cross-sectional (5)<br>Prospective (2)	EU (3)<br>Asia (1)<br>Unspecified (3)	Standardised abstraction procedure (Centre for Reviews and Dissemination 2001)	MD	Permanent night (2)<br>Rotating (2)<br>Evening (1)<br>Unspecified (3)	No	Smokers, % (5);<br>Cigarettes/day (2)

N: Number of participants; (n): number of studies concerned; yo: years old; MD: missing data

Supplemental table H. Characteristics of systematic reviews focused on the link between shift work and occupational psychosocial stressors

First Author, Year publication, Country	Characteristics of population		Setting and context	Database searching		Studies included in each review				Quality of the primary studies		Shift work		Assessment of outcomes (n)
	Gender, age [mean, range] (n)	N participants [min-max]		Source	Date range	Publication date range	n	Type (n)	Countries of origin	Instrument used	Rating (n)	Type (n)	Exposure duration (n)	
**Angerer**<br>**2017**<br>**Germany**<br>**Meta-analysis**	♂,♀ and both<br>Age range<br>[17-73] (5)<br>Mean age range<br>[18.9-47.2] (7)	32,825<br>[60-9,765]	Industry (1)<br>Health care (6)<br>Heterogeneous (4)	PubMed<br>Scopus<br>PsycINFO<br>PSYNDEX<br>Medpilot	Until<br>10/2015	1989-2015	11	Prospective (11)	Asia (2)<br>EU (9)	QUIPS checklist	High (11)	Permanent Night (5)<br>Rotating (11)<br>Irregular (2)	No	**Depression:** GHQ-12; HADS; COPSOQ; Prescriptions of antidepressants; Psychiatric interview; ICD
**Tahghighi**<br>**2017**<br>**Australia**	♀ and both<br>Age MD	79,627<br>[13-25,924]	Health care (37)	Cinahl<br>PubMed<br>Medline<br>Embase<br>Google scholar<br>Grey literature	1995-2016	1997-2016	37	Cross sectional (32)<br>Longitudinal (5)	EU (12)<br>America (4)<br>Asia (21)	Mixed Methods Appraisal Tool by Pluye et al.	High (27)<br>Moderate (10)	Shift work (5)<br>Rotating (28)<br>Permanent night (17)	No	**Well-being/Quality of Life**: 1 item measure of well-being; Scale of the negative effects of work time; Conflict between work and family rating scale; Chinese health questionnaire 12-item; WHOQOL-BREF<br>**Job satisfaction:** Job satisfaction scales; Standard shift work index questionnaire; Job, family and life satisfaction scale<br>**Burnout:** MBI; CBI; Job stress questionnaire from the Korean occupational stress scale<br>**Depression, Anxiety and Stress:** NSS; BDI-II; CES-D; PHQ-9; HAD-S; Taiwan nurse stress checklist; STAI-Y; Profile of mood states; GHQ-12<br>**Resilience and Coping**: Coping questionnaire; Hardiness and resilience Scales
**Zhao**<br>**2019**<br>**Australia**<br>**Meta-analysis**	♂,♀ and both<br>Age ≥15-18 yo (7)<br>Age range<br>[17-73] (11)<br>Mean range<br>[37-47] (7)<br>MD (8)	249,196<br>[132->50,000]	Industry (1)<br>Heterogeneous (24)<br>Unspecified (8)	PubMed<br>PsycINFO<br>Web of Science<br>Scopus	Until<br>12/2017	2002-2017	33	Cross sectional (22)<br>Longitudinal (11)	EU (7)<br>America (16)<br>Asia (10)	NOS	Cross sectional quality range [0.5-3]/3<br>Longitudinal quality range [1.5-4]/5	Shift work (12)<br>Rotating (5)<br>Permanent night or evening (12)<br>Irregular (14)	Yes (2)	**General mental health**: Kessler-6 (4); SF-36/SF-12 (7); GHQ-12 (5)<br>ILfeld psychiatric symptoms index (3)<br>**Depression:** CES-D (8); BDI (3); WHO wellbeing scale (3)<br>NHP (1); CIDI-SF (1); PHQ-9 (1); HAD-S (1); STAI-Y (1)

N: Number of participants; (n): number of studies concerned; yo: years old; MD: missing data

Supplemental table I. Characteristics of systematic reviews focused on the link between shift work and sedentariness

First Author, Year publication, Country	Characteristics of population		Setting and context	Database searching		Studies included in each review				Quality of the primary studies		Shift work		Assessment of outcomes (n)
	Gender, age [mean, range] (n)	N participants [min-max]		Source	Date range	Publication date range	n	Type (n)	Countries of origin	Instrument used	Rating (n)	Type (n)	Exposure duration (n)	
**Monnaatsie 2021 Australia Meta-analysis**	♀, ♂and both<br>Mean range<br>[27-49.3] yo (49)	310,710<br>[9-185,958]	Industry (8)<br>Health care (24)<br>Heterogeneous (12)<br>Administration (4)<br>Unspecified (1)	Ebscohost, PubMed, Scopus, Web of Science, Science Direct	Until 04/2021	2001-2021	49	Cross-sectional (45)<br>Prospective (2)<br>Cohort (2)	EU (19)<br>America (14)<br>Asia (16)	STROBE	High(38)<br>Moderate (11)	Shift work (26)<br>Rotating (13)<br>Night (25)	No	IPAQ (9)<br>Other questionnaire (22)<br>Self-report (3)<br>Actigraph (14)<br>Accelerometer (3)<br>Calorie counter (1)
**Crowther 2022 Australia**	♀ and ♂<br>Age classes<br>[≤39-≥50] yo (1)<br>Mean age<br>[26.8-31.5] yo (2)	29,701<br>[159-29,019]	Health care (1)<br>Heterogeneous (2)	Embase, Medline, PsycInfo, Web of Science	Until 03/2021	2004-2020	3	Prospective (3)	EU (3)	CASP Cohort checklist	Undetailed	Permanent night (2)<br>Rotating (3)	No	Questionnaire (3)

N: Number of participants; (n): number of studies concerned; yo: years old; MD: missing data
